# How Brazilian dentists work within a new community care context? A qualitative study

**DOI:** 10.1371/journal.pone.0216640

**Published:** 2019-05-08

**Authors:** Pedro Augusto Thiene Leme, Silvia Amélia Scudeler Vedovello, Rodrigo Almeida Bastos, Egberto Ribeiro Turato, Carlos Botazzo, Marcelo de Castro Meneghim

**Affiliations:** 1 Department of Social Dentistry, Piracicaba Dental School, University of Campinas, Piracicaba, São Paulo, Brazil; 2 Department of Orthodontics, Herminio Ometto Foundation, Uniararas, Araras, São Paulo, Brazil; 3 Department of Obstetrics and Gynecology, School of Medical Sciences, University of Campinas, Campinas, São Paulo, Brazil; 4 Department of Clinical Psychology and Psychiatry, School of Medical Sciences, University of Campinas, Campinas, São Paulo, Brazil; 5 Department of Policy, Management and Health, School of Public Health, University of São Paulo, São Paulo, Brazil; Newcastle University, UNITED KINGDOM

## Abstract

The aim of this study was to analyze the value and meanings that dental surgeons attribute to the Primary Health Care setting, where health promotion is encouraged over a mechanistic performance of procedures. A qualitative study, involving ten Brazilian dental surgeons working in Primary Care in 2016, was designed. In-depth semi-structured interviews were performed, with all interviews recorded, transcribed and subsequently submitted to Qualitative Content Analysis. Despite the Healthcare Promotion model proposed by the Brazilian oral health policy, dental surgeons demonstrated preferences for private and traditional dental practices. These characteristics are counterproductive in public oral health services, which aim to achieve collective health benefits. Traditional practice is based upon a specific and restricted focus, as opposed to overall patient care, hence maintaining the original professional identity, ruled by manual procedures, while demonstrating scientifically fragile understanding of disease processes. Despite the implementation of public service models that aim at change, counterproductive characteristics associated with the deeply rooted traditional management strategies were evidenced.

## Introduction

Despite existing for more than a century, dentistry continues to struggle in proving its effectiveness when facing the health needs of the population [1]. Since its origin, dentistry has prioritized practical and surgical techniques, based on prosthetics reabilitations and dental materials, isolating itself from other healthcare specialties. An antiquated point of view in the face of global socioeconomic problems and higher chronic disease burden within the population [2,3].

In Brazil, due to political and historical motivations, a lobby called “Collective Health”, which favors an angle more closely related to social and structural factors of diseases, was created to counteract traditional public health practices, namely the biologic approach. Such influence led to the formulation and testing of different models of dentistry in Brazil [4,5].

A significant part of Brazilian public oral health services are located in Primary Care, more specifically within "family health teams", who implement individual, family-based and collective health actions that involve promotion, prevention, protection, diagnosis, treatment, rehabilitation, harm reduction and palliative care. These services are offered free of charge to all people living within Brazilian territory, according to their needs. These multidisciplinary teams consist of a medical doctor, nurse, nursing assistant, and community health agents. The oral health teams may also be included [6].

The family health teams are responsible for 2000 to 3500 people within a defined region. Defined populations and continuous provision of care means that bonds and co-responsibility between the teams and the population are favorable. This allows for reliable monitoring of the effects of health interventions, hence reducing the risk of iatrogenic events, which are often associated to the lack of knowledge on an individual’s life history, as well as poor care coordination [6].

The introduction of the National Oral Health Policy of Brazil (from Portuguese, PNSB), in 2004, was one of few global experiences in which public dental care was incorporated into Primary Health Care (PHC), attaining national coverage [7,8]. Countries such as Canada, which have well-established public health systems, are still unable to offer free access to public oral healthcare [9].

The oral health actions provided in the PNSB can be divided into: addition of fluoride to water sources; health education in the form of debates, workshops, videos, theaters, groups, posters and leaflets; supervised dental brushing; topical application of fluoride; diagnosis and treatment of soft and hard tissue lesions of the oral cavity, within the scope of primary care, aided by clinical care provided by the oral health team in the Family Health Care Units, notably the focus of the present study; rehabilitation actions, shared between primary and secondary care, with the support of the Dental Speciality Centers (from Portuguese CEO) that offer services for oral cancer, care for patients with special needs, endodontics, periodontics, and more complex surgery [8].

The new guidelines go beyond traditional dental approaches, as they promote advances in the work process. The concept of health promotion is the theoretical guideline of this model, moving the focus from provision of manual procedures to an approach that promotes support, information, responsibility and autonomy, which should also apply to the dental work provided in community-based multidisciplinary health units, known as Family Health Care Units [6,8].

In order to develop patient autonomy, the healthcare team should endeavor to encourage self-care practices, reducing medicalization and excessive dependence on healthcare professionals or services. For this purpose, teams need to understand the patients under their responsibility, their living conditions, the representations and values that they attribute to health, their habits and the actions required to solve problems related to illness and disease prevention [6].

Innovations aimed at dental practices that plan to overcome traditional management strategies, have been presented. These include the pursuit of biopsychosocial completeness, creating therapeutic bonds and gathering valuable information on the users’ family and work background, alongside multi-professional teamwork [10–13].

One may argue, therefore, that the new Brazilian oral health policy anticipates the implementation of a modern practice that is similar to the proposals of the "La Cascada Declaration" and are debated in current academic circles: control of common oral diseases by a community health care worker, integration of oral health into other policies and other areas of health, revision of the training curriculum, implementation of a practice where maintenance of preserved dentition overlaps financial interests in procedures, hence reducing unnecessary procedures [1–3].

Over a decade after the founding of PNSB, one should analyze in detail the reality of daily practice. One should contemplate if the dental surgeons working within PNSB embody these intentions of the health promotion approach or remain fixed to a surgical approach for one of the most common dental diseases—caries. Considering dental caries as a common theme, this study sought to approach it as the driving force for the interviews. The study aimed to clarify the response to the following question: do dental surgeons working within this new model have a broader outlook on patient care?

To appropriately analyze this at the intended level is a complex task, since clinical activity occurs in a unique way and is interspersed by interests, values as well as private and subjective tensions, hence demanding studies performed in a qualitative perspective in their natural settings [14]. The aim of this study was to understand the meanings and values that dental surgeons attribute to their practice in the Primary Healthcare setting, in order to identify congruences and contradictions to the proposals of the PNSB.

## Methods

### Overview

A qualitative phenomenology based study was designed, detailed by the Clinical-Qualitative Method [14,15] which allows the understanding of personal values and meanings that patients or professionals attach to issues of the health-disease process.

In qualitative research, phenomena are studied in their natural environments, in order to prevent them from being controlled. They have their own characteristics regarding sampling, data analysis, and possible generalization of results [15].

The instrument used to collect data was the semi-structured in-depth interview with open-ended questions [16].

### Sampling and recruitment

The sampling method of exhaustion was used, a technique that aims to include all eligible subjects within an determined population [17]. In qualitative studies, the commonest method to determine when to restrict the inclusion of new participants is the sample saturation criterion [17,18]. In the present study, despite theoretical saturation of information being achieved during the eighth interview, the moment at which the interviews became repetitive, all eligible subjects from the study population were included as initially planned.

Therefore, ten dental surgeons working in family healthcare units of the PHC in the Brazilian Unified Health System (SUS), in a town of approximately 400,000 inhabitants, in the southeastern region of Brazil, were included. The inclusion criteria comprised participants with a minimum of two years experience in their current position, employment achieved via a public selection process and expressed consent to participate in the study. Of the 14 dentists that fulfilled the inclusion criteria, one was excluded from the final sample due to their participation in a preliminary study (pilot), two declined to partake for personal reasons and one did not respond. Data collection took place between January and October 2016.

Initial contact with dental surgeons was achieved via an e-mail containing a brief explanation of the research, as well as an invitation to participate. After acceptance of the invitation, face-to-face interviews were arranged, where informed consent was obtained, allowing the start of an observational stage. The researcher observed the routine of each healthcare service in order to take field notes, adapt to the environment and to gain the confidence of the interviewees, promoting mutual recognition and reiterating the goals and interests of the research, ensuring material validity. These field notes, which were a record of observed context, were subsequently used to improve and clarify the interviewers understanding of experiences recounted by the interviewees during the content analysis.

### Ethics approval

This research project was approved by the Ethics Committee of Piracicaba Dental School, University of Campinas, Piracicaba, SP, (number 49524715.6.0000.5418) [19]. All procedures were in accordance with the ethical standards of this committee and with the most recent revision of the Helsinki Declaration.

### Qualitative data collection

Interviews were carried out in the Family Health Care units between the examiner and interviewee only. These interviews were audio recorded and transcribed by the main researcher, a male dental surgeon with a masters degree in Public Health and PhD student in Public Health. There was no determined time-limit for the interviews. Each interviewee participated once in the study, as repeated interviews with the same participant were unnecessary.

In the preparation stage of the project, guidance and support were obtained, with training and calibration of the interviewer (main researcher) by: 1- presentation of the results obtained during the pilot for the group of the Laboratory of Clinical-Qualitative Research (LPCQ) of the University of Campinas (UNICAMP), formed by a multi-disciplinary team constituted by medical doctors, nurses, psychologists, nutritionists and dental surgeons with experience in interviewing, who evaluated performance and offered suggestions regarding the content of the interview guide and to the posture of the interviewer; 2- a new trip to the research field to run another interview with the orientations suggested by the group; 3- presentation of the results of the second interview to the same group, who, after a new evaluation, confirmed the suitabiliity of the new interview guide, as well as the interviewer posture, which were then maintained until the end of the interview process [14].

The interviews were conducted with the semi-structured interview guide, composed by an open question: *How do you feel about caries and what is your experience regarding its treatment*? During the course of the interview, complementary questions were used in order to revisit the initial objectives, should the interviewee not approach these topics spontaneously:

- Have you come across patients that always present new carious lesions at follow-up appointments? How do you feel about it?
- When applying preventative measures, we use strategies such as oral hygiene or diet, what do you think about these? Tell us your experience on the subject.
- How do patients with caries deal with their diagnosis?
- What are your experiences on caries counseling and patient counseling?
- In our day-to-day clinical lives, we often experience moments that affect us personally. Please tell us about a situation that has had an emotional impact on you in a patient with caries.

The interviews were ended with the open-ended question—*Would you like to comment on something that you have not been asked or is there anything that you would like to add*?

### Data analysis

**Fig 1:**
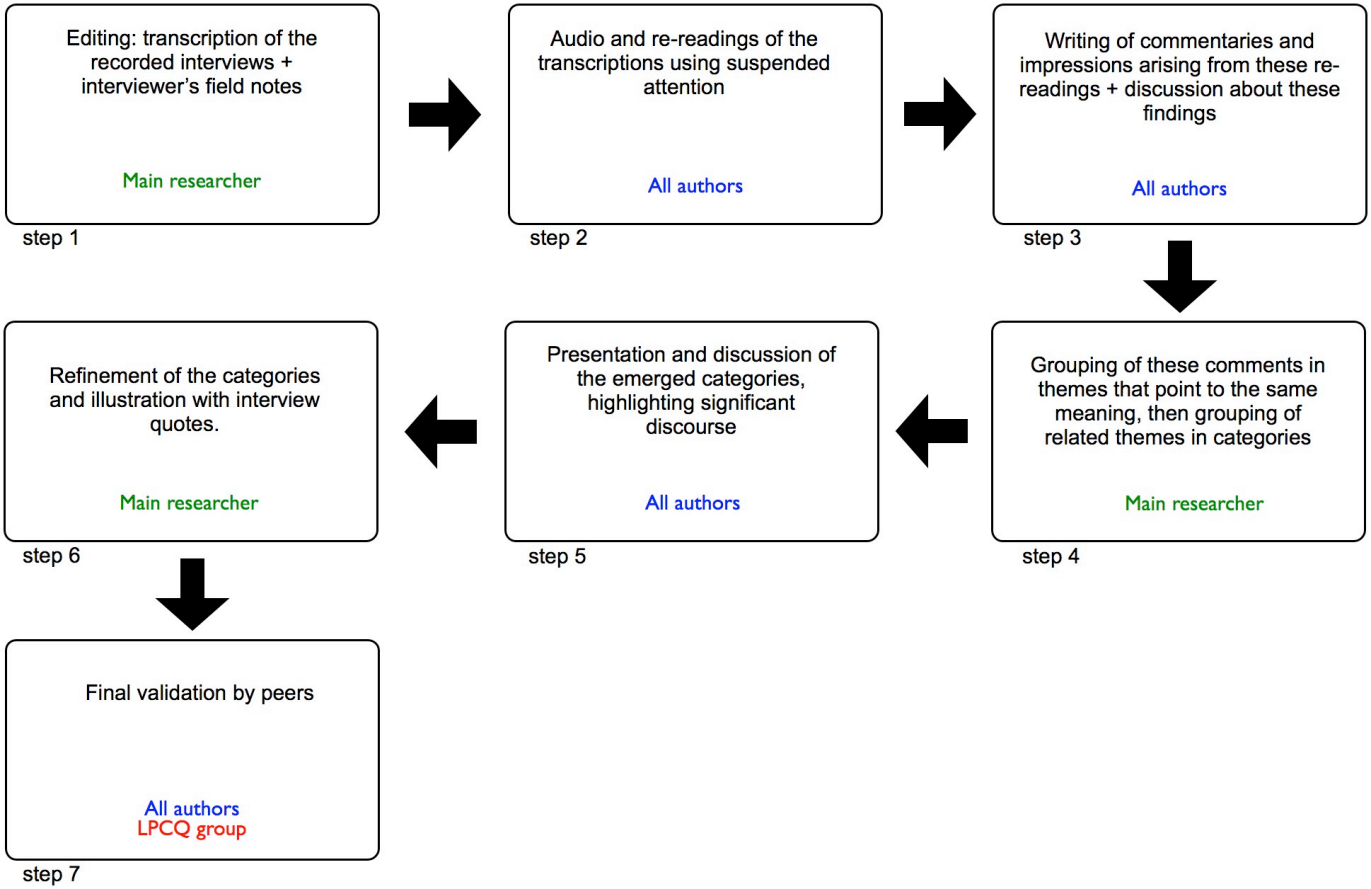
Flow chart of the content analysis. Adapted from Faria-Schutzer et al [20].

Data was explored by inductive content analysis and included complete transcription of the interviews, re-readings using suspended attention, as well as elaboration and classification of comments (Fig 1) [14,20]. This was achieved using a basic text editing software for reading and preparation of comments. Analysis was performed by the main researcher, alongside a group of five invited researchers (co-authors) (Fig 1). Each co-author was trained and calibrated by: 1—presentation of the project by the main researcher, who reported a review of the literature and the objectives of the study; and 2 –review of the technique and steps required for the method presented, with the support of experienced researchers in this area.

The process of division by themes was started using steps 2 and 3, in which all authors evaluated the transcriptions using suspended attention, re-readings and commentaries were made. Each comment was linked to excerpts (quotes) from the interviews. The main researcher then organized the comments and excerpts provided by the authors, subsequently grouping them into themes. Associated themes then gave rise to themes, accompanied by quotes from the interviews.

After organization of the themes, the main researcher presented and discussed the results with all authors (step 5, Fig 1), aiming to meticulous refine the material according to the suggestions, as well as to select appropriate quotes that best represented the principle idea of each theme (step 6, Fig 1). The themes were then validated by the LPCQ group (step 7, Fig 1), by means of a presentation by the main researcher regarding the representative themes and quotes. The group subsequently provided suggestions based on the following criteria: 1—heterogeneity between themes; 2—internal homogeneity of themes; and 3—suitability of the name of each theme. Finally, the required adjustments were applied.

## Results

### Overview of findings

Dental surgeons were invited to talk about their thoughts, feelings and experiences regarding the treatment of dental caries, achieved by interviews lasting 30–50 minutes, according to the individuals willingness to express their opinions. Four main themes emerged from the data analyzed: (1) Therapy centered on the tooth, not the person; (2) Dental caries: an imprecise pretext for dental therapy; (3) Dental appointments as a synonym for manual intervention; and (4) Outmoded concepts of disease. Numbers preceded by the letter “E” at the end of discursive excerpts indicate the participants code. Sociodemographic characteristics of the sample are described in Table 1.

**Table 1:** Sociodemographic characteristics of the dental surgeons from Family Health Care Units, Piracicaba, SP, 2016.

**Sex**	**N**
Female	6
Male	4
**Age**	
30–39	6
40 or >	4
**Year of graduation**	
2000–2009	6
1990–1999	3
1980–1989	1
**Concluded postgraduate courses**	
Continuing education courses	10
Master’s	5
Doctorate	2
**Area of such courses**	
Public or Family Health	8
Oral Surgery	4
Orthodontics	3
Pediatric Dentistry	2
Others	4

### Treatment centered on the tooth, not the person

Although the new model promotes aspects of person-centered therapy, the conventional perspective in which the focus is on the disease, or more specifically, the damaged tooth, as if it were an entity separate from the general physiology of body and society, is what prevails. Therefore, the therapeutic support offered automatically focuses on the problem of patients hygiene habits, and the dentist cannot diagnose the reason for treatment failure:

*And so far*, *I haven’t been able to identify it* (the reason for treatment failure), *unless this patient wasn’t able to establish appropriate oral hygiene habit*…

(E4)

Tension is observed when dental practice is applied to a socioeconomically disadvantaged population. The feeling of discomfort by the dental surgeons is clear, and reinforces the idea that, limited by the traditional strategies acquired in their dental training, they feel unprepared to work in the context of Primary Health Care:

*Sometimes I lose my patience with that person that I’ve already told ten times*, *yet they come back without brushing their teeth*, *full of plaque*. *I start talking about this*: *we won’t fill it anymore while this plaque is here*.

(E2)

The reason behind the patients inability to maintain their own oral hygiene is often not considered in the clinical setting, with emphasis being given to diagnosing the presence or absence of biofilm.

The dental perspective, centered on the management of diseases, still prevails and supports the utopia of satisfying the oral requirements of the community through tooth repair, while ignoring the broader social and ecological causalities. When objectifying the teeth and considering them as a separate entity from the patient, the dental surgeon believes that health issues are due to repressed demand for services, without perceiving the social determination.

The belief in the traditional working values that promote technical skills with little consideration for broader aspects, described in the literature as "drill and fill" *modus operandi*, was repeatedly observed. Furthermore, this is more evident through the frustration shown when the dental surgeon observes worsened oral hygiene after treatment:

*I treated him*, *and then he comes back after a while*, *there’s stuff to do*, *yeah*… *So*, *we feel like*… *Our efforts did not work*.

(E9)

### Dental caries: An imprecise pretext for dental treatment

The question that invited the dental surgeons to talk about caries allowed diverse values to be attributed, with caries being conceived as a disease, cavities, white spots, paralyzed lesions, and even defective restorations.

Understanding caries as a disease process:

*So*, *caries*, *as far as we learn and when taught to us*, *is a multifactorial disease*, *right*?

(E10)

Understanding caries as a lesion:

*I only really learned to remove caries here*, *in this public service*, *right*… *So*, *you get in there*, *quickly remove all caries (*…*) the first time the patient comes with twenty caries*, *the next year he has six*.

(E5)

As a cavity:

*Most patients already present with caries*, *big cavities*, *which makes it easier for me to identify if there’s caries or not*.

(E2)

Experience of treatment of caries was understood as a synonym of drill and fill:

*Experience*… *Hmm*, *yeah*, *we do fillings right*, *amalgam*, *resin*, *ionomer*, *right*. *Most of what we do here includes fillings*.

(E8)

When talking about caries, the dental surgeons referred to restoration replacement as a treatment for caries. Caries and “imperfect” restoration are, to the dental surgeon, synonyms:

*So*, *for me it is very complicated to examine and evaluate each resin to see if it’s good*, *or if I have to replace it*, *because usually it isn’t*. *It’s rare for you to see a perfect mouth* (referring to esthetically pleasing fillings) *where you have nothing to do*.

(E2)

The fact that some dental surgeons mentioned diagnosing many caries and, in the same interview, contradict themselves by saying that they see few caries, reinforces our hypothesis that there is a conceptual imprecision about what is understood by *caries*:

*What we diagnose most are caries*.

(E1)

*Prevalence of caries is very low*.

(E1)

It’s still the most common disease, right, as we all know.

(E6)

*There aren’t many*, *thank God*, *we don’t have many cases like this*, *patients with lots of cavities*.

(E6)

### Dental appointment as synonym of manual intervention

In this study, the idea of dental appointments as a synonym for intervention was a frequent finding during the interviews. Patient-dental surgeon interaction and counseling, with the potential for non-procedural management plans were not considered the type of work that should be fulfilled by the dental surgeon:

*I gave advice about brushing*, *demonstrated it using the mirror*, *and lost a whole session only doing that*.

(E2)

*If I stop everyone and say*, *look*, *let’s learn about tooth brushing*, *and demonstrate it*, *I won’t have time to see everyone*.

(E5)

### Outmoded concepts about diseases

Current scientific understanding of oral diseases, such as caries and periodontal disease, has superceded the concept that these are classic infectious diseases, where a specific pathogen is involved, and are now understood as being caused by "dysbiosis", an imbalance of microbial flora that, in ideal conditions, coexist in a harmonious relationship [21]. However, the dialogues revealed ideas that caries are transmitted, being potentially infectious, providing evidence of the limits between contemporary scientific knowledge and clinical practice:

*(*….*) They* (patients) *don’t understand that caries is a disease process*, *can be passed on (*…*) that they are contagious*

(E9)

As a consequence of this notion regarding the infectious causality of caries, dentistry has failed in replacing mechanistic, invasive and inadequate treatment modalities in daily routine to an approach aimed at controlling causal factors, as the focus on the consumption of free-sugars [22]

*Where there is caries you have to drill it*, *it doesn’t matter if you hit the pulp or the periodontal ligament*, *you have to remove it all*, *because if you leave any there*, *it will grow and cause more problems*, *right*…

(E5)

## Discussion

A recent literature review on oral healthcare in PHC revealed difficulties regarding overcoming conventional dental practices, with persistence of management focused primarily on technique [23]. This was also identified in the present study, indicating that, despite the intention of a broad reformulation by the new model, dental practice is strongly rooted in tradition, even when applied to a PHC. Dental surgeons still value their work through a perspective centered on a conventional point of view, which favors mechanistic interventions. This was described by Warmling et al [24], who reported the *original professional identity*, revealing the influences of dental teaching policies on the shaping of the dental surgeons identity, emphasizing that the original dental curriculum makes no mention of a clinic, which have been restricted to medical courses.

One may consider that this is the likely reason that certain ideas endure despite the creation and application of modern denistry. Therefore, this study corroborates the idea that a restricted dental focus continues to guide principle ideas of prevention, education and rehabilitation. The meaning attributed to treatment and healthcare, thus, rarely exceeded the limits of the tooth, biofilm, sugar, hygiene and restoration.

This phenomenon may be regarded as a barrier to practice improvement, since restricting the treatment plan in this manner disregards the importance of a person centered approach, which is more appropriate than simply performing technical procedures and obsessing over oral hygiene.

The PNSB guidelines involve aspects of the person-centered approach, by encouraging the interrelationships over time, defending the common risk factor approach, valuing health concerns and experiences [8,23]. Clinical practice, however, continues to be centered around manual procedures, while the importance of the holistic approach to both patient and community are ignored. Baldani et al. [25] argued that, in this context, a practice based on humanization is employed. However, their study was based on the evaluation of simpler aspects of the dental surgeons opinions.

The almost exclusive emphasis of proximal etiological factors for dental caries is a complicating factor in the patient-dental surgeon relationship, which may reduce treatment compliance and perpetuate natural disease progression. This patient-healthcare professional bond is a critical therapeutic resource, which assumes an exchange of knowledge between the professionals and peoples concerns, objective and subjective themes [26].

In terms of beliefs regarding bacterial plaque, dental surgeons were observed to disregard wider factors of the disease process, restricting themselves to biological and individual ideations of the disease, rather than concern for social aspects [27].

Uncertainty regarding dental caries concepts resulted in imprecision to define the focus of the dental surgeons work. Botazzo [27] suggested that caries are perceived as a *fetish* for dental surgeons, since they are a a diffuse, polymorphic and indefinite element, which allow for the generic “drill and fill” approach adopted by the profession. Comments arising from restorative and cosmetic dentistry, the industry and common sense, synergistically justify any form of dental interventions.

The multiple meanings given to caries also explains the inconsistency of perception of their prevalence, with contradiction between witnessing several or few cases of caries, in accordance with the statement that “dentists, when talking about caries, don’t know anymore what they’re talking about” [27] and thus dental procedures are performed in an automatic and unreflective manner, preserving mechanical tradition in detriment to the new guidelines.

Regarding the overlap observed between caries and need for fillings, one may refer to the Baders and Shugars [28] model. The authors aimed to explain clinical decision by dental surgeons regarding the treatment for caries, suggesting that they operate via pattern recognition from “mental scripts” in which detection of alteration is automatically related to the interventionist decision [28].

The dental surgeons preference for mechanical treatment is well documented in the literature, with a common recurrence in articles for cariology that focus on the biological-centered perspective for dental caries [2,29–32].

Baelum [2] stated that high-speed dental instruments represent the devotion of most dental surgeons to traditional treatments, as well as to unnecessarily encourage the power of these interventions in oral health promotion, classifying dental surgeons as “dental mechanics”. Renshaw [33] attributed an obsession for all that is technical to dental surgeons. Baelum et al. [11] considered the “drill and fill” paradigm as consolidated in several ways, with concern over the difficulties of breaking such a vicious cycle.

Dental surgeons have been observed to consider dental appointments as synonymous with manual procedures, which is relevant when considering the context in this study. The professional being paid on a production basis is pointed out in literature as being one of the main inducers of technicality [2,34,35]. Tooth, isolated from body and society, is a simpler intervention object; therefore the procedure-centered practice fits well [24,27,36,37].

Finally, outdated scientific ideas regarding disease helps to explain more invasive approaches from a technical point of view. Although, discussion on the technical aspects of disease management is not the focus of our study, it is important to highlight that current evidence demands a minimally invasive approach when surgical interventions are required, allowing preservation of physiological structures and avoiding possible repetitive restoration cycles [22,38–40]. The concept of dental caries being contagious has been scientifically disproven, although it is still prevalent in dental surgeon’ statements [21,22].

The literature reveals examples of experiences of health promotion in different countries that illustrates the limitations of dentistry. Saekel [41] used a thought-provoking analysis of German public oral health policies to aid decisions on the implementation of an appropriate system in China. The author condemned the catastrophic oral health consequences of an invasive and restorative approach that prevailed over a long period of time throught Germany, instead showing the benefits, although partial and insufficient, obtained after its reform in the 1980s. The idea that a *modus operandi* anchored in outdated assumptions about oral diseases, namely the "Drill and Fill" type, when implemented through wide public policies, collaborates the perpetuation of oral disease burden [41].

Fejerskov et al. [3] described a series of studies from countries including as Denmark, Finland and Norway, that have implemented different successful paradigm-shifting experiences in oral health services by incorporating up-to-date diagnostic and therapeutic approaches, and demonstrated the potential of achieving epidemiological benefits from policy shifts.

The PNSB has many differences compared to the aforementioned proposals, including several advances such as its inclusion in a more comprehensive health policy of Primary Health Care, multiprofessional teamwork, its allocation within Family Care Units, continuous monitoring over time, among others. However, advances to the reality of the practice model, moving from a surgical to a health promotion approach, are required.

Potential means to change dental providers’ ideas from surgical to health promotion have been discussed in the literature. The dental curriculum is a growing concern, demonstrated by dental schools around the world seeking to provide students with community-based experiences, increasing the number of interdisciplinary courses, and blending basic with clinical sciences [42,43]. Extramural training in Brazil provides dental students with enriching experiences by placing them in different socioeconomic environments, that are often very different from their own background, allowing improved training and more adequate practice [43]. However, barriers involving difficult placements are often identified, some linked to the values and interests that permeate the practice and training of dentists, as well as academic political ideas, hence limiting the development of new attitudes in dental surgeons [43].

Considering the inability of dentistry to provide effective oral healthcare, Fejeskov et al [3] described the need for a new cadre of dental professionals capable of meeting the current health needs based on scientifically up-to-date knowledge. The authors of the current study believe that while such profound changes are not implemented, some of the recommendations could be adapted and applied in the current dentistry cuuriculum, as well as in continuing education cycles for dentists already trained and employed within oral health policies.

## Study limitations

The qualitative study performed does not allow one to generalize over the results obtained, which refers to a restricted population, studied within a specific context. However, one hopes that the findings are relevant to contribute to a broader discussion, since there is a current global effort to transform dental practice towards the health promotion aproach, and interested parties must consider the similarities and contextual differences compared to those presented in this study.

Another limitations of the present study are linked to the interviewer. Primarily, despite nine years of healthcare experience, the aforementioned had no previous experience with formal interviews, although similarities regarding comprehensive listening should be considered. Secondly, interviews were conducted by a dental surgeon interviewing professionals from the same field, which could reduce the objectivity of the study and the degree of freedom on the part of the participants. On the other hand, facilitation of communication should be considered, as the participants could use the specific language and jargon, thus providing their ideas and arguments spontaneously and naturally.

## Conclusions

The findings from this study revealed counterproductive values and meanings associated with traditional treatment modalities, even in an oral healthcare model that advocates for innovations. To promote oral healthcare in diverse populations, there is a need to assign more than simply traditional dental surgeons to different healthcare models. Internationally, there is also an urgent need to implement a reform in dentistry, or to introduce new modalities.

Our study may be relevant to other countries aiming to implement oral healthcare in public health services. Public policies need to identify the limitations of dentistry and to act to optimize health in the population.

## Supporting information

S1 File COREQ checklist. (PDF)

S2 File Transcriptions in Portuguese. Complete transcript of interviews presented in Portuguese.
(DOCX)

S3 File Interview guide. Script used to conduct the interviews.
(DOCX)

## References

[1] Cohen LC, Dahlen G, Escobar A, Fejerskov O, Jonhson NW, Manji F. Dentistry in crisis: time to change. La Cascada Declaration. Aust Dent J. 2017;62(3):258–260. 10.1111/adj.12546 28793371

[2] Baelum V. Caries management: technical solutions to biological problems or evidence-based care? J Oral Rehabil. 2008;35(2):135–51. 10.1111/j.1365-2842.2007.01784.x 18197847

[3] Fejerskov O, Escobar G, Jøssing M, Baelum V. A functional natural dentition for all--and for life? The oral healthcare system needs revision. J Oral Rehabil. 2013;40(9):707–22. 10.1111/joor.12082 23855597

[4] Vieira-da-Silva LM, Pinelli P. The genesis of collective health in Brazil. Sociol Health Illn. 2014;36(3):432–46. 10.1111/1467-9566.12069 24111568

[5] Narvai P. Collective oral health: ways from sanitary dentistry to buccality. Rev Saude Publica. 2006;40(SN):141–7. 16924314 10.1590/s0034-89102006000400019

[6] Brasil. Ministério da Saúde. Portaria 2.436 de 21 de setembro de 2017. Brasília: Diário Oficial [da] República Federativa do Brasil, 2017.

[7] Pucca GA, Gabriel M, de Araujo ME, de Almeida FC. Ten Years of a National Oral Health Policy in Brazil: Innovation, Boldness, and Numerous Challenges. J Dent Res. 2015;94(10):1333–7. 10.1177/0022034515599979 26316461

[8] Brasil. Ministério da Saúde. Departamento de Atenção Básica. Coordenação Nacional de Saúde Bucal. Diretrizes da Política Nacional de Saúde Bucal. Brasília; 2004.

[9] quebecsolidaire.net [internet]. Il est temps d’avoir une assurance dentaire publique au Québec! [Cited 02 March 2018]. https://appuyez.quebecsolidaire.net/assurance-dentaire.

[10] Baelum V. Dentistry and population approaches for preventing dental diseases. J Dent. 2011;39(2):S9–19. 10.1016/j.jdent.2011.10.015 22079282

[11] Baelum V, van Palenstein Helderman W, Hugoson A, Yee R, Fejerskov O. A global perspective on changes in the burden of caries and periodontitis: implications for dentistry. J Oral Rehabil. 2007;34(12):872–906. 10.1111/j.1365-2842.2007.01799.x 18034671

[12] Usha C, Sathyanarayanan R. Dental caries—A complete changeover (Part I). J Conserv Dent. 2009;12(2):46–54. 10.4103/0972-0707.55617 20617066 PMC2898091

[13] Elderton RJ. Preventive (evidence-based) approach to quality general dental care. Med Princ Pract. 2003;12(1):12–21. 10.1159/000069841 12707497

[14] Turato ER. [Treatise on clinical-qualitative research methodology: Theoretical-epistemological constrution, comparative discussion and application in the health and humanities areas.] 5th ed Petropolis: Vozes; 2003.

[15] Turato ER. Qualitative and quantitative methods in health: definitions, differences and research subjects. Rev Saude Publica. 2005;39(3):507–14. 15997330 10.1590/s0034-89102005000300025

[16] Fontanella BJB, Campos CJ, Turato ER. Data collection in clinical-qualitative research: use of non-directed interviews with open-ended questions by health professionals. Rev Lat Am Enfermagem. 2006;14(5):812–20. 10.1590/S0104-11692006000500025 17117269

[17] Fontanella BJB, Luchesi BM, Saidel MGB, Ricas J, Turato ER, Melo DG. Sampling in qualitative research: a proposal for procedures to detect theoretical saturation. Cad Saude Publica. 2008;27(2):388–394. 10.1590/S0102-311X2011000200020 21359475

[18] Glaser BG, Strauss AL. The Discovery of Grounded Theory: Strategy for Qualitative Research. New Brunswick: Aldine Transaction, 1999.

[19] Brasil. Ministério da Saúde. Plataforma Brasil. [Cited 03 Mar 2018]. [Internet] http://plataformabrasil.saude.gov.br/login.jsf.

[20] Faria-Schützer DB, Surita FG, Alves VL, Vieira CM, Turato ER. Emotional Experiences of Obese Women with Adequate Gestational Weight Variation: A Qualitative Study. PLoS One. 2015;10(11):e0141879 10.1371/journal.pone.0141879 26529600 PMC4631528

[21] Kilian M, Chapple ILC, Hannig M, Marsh PD, Meuric V, Pedersen AML, et al The oral microbiome—an update for oral healthcare professionals. BDJ Open. 2016;221:657–666. 10.1038/sj.bdj.2016.865 27857087

[22] Sheiham A, James WP. Diet and dental caries: the pivotal role of free sugars reemphasized. J Dent Res. 2015;94:1341–1347. 10.1177/0022034515590377 26261186

[23] Scherer CI, Scherer MD. Advances and challenges in oral health after a decade of the "Smiling Brazil" Program. Rev Saude Publica. 2015;49:98 10.1590/S0034-8910.2015049005961 26815162 PMC4760711

[24] Warmling CM, Marzola NR, Botazzo C. On the autonomy of the mouth: curricular practices, professional identity, and the emergence of dental teaching in Brazil. Hist Cienc Saude Manguinhos. 2012;19(1):181–95. 10.1590/S0104-59702012000100010 22488381

[25] Baldani MH, Fadel CB, Possamai T, Queiroz MG. Inclusion of oral health services in the Family Health Program in the State of Paraná, Brazil. Cad Saude Publica. 2005;21(4):1026–35. 16021240 10.1590/s0102-311x2005000400005

[26] Santos AM, Assis MM, Nascimento MA, Jorge MS. Bond and autonomy of the oral health practice in the Family Health Program. Rev Saude Publica. 2008;42(3):464–70. 10.1590/S0034-89102008005000025 18461254

[27] Botazzo C. Diálogos sobre a boca [Speeches about the mouth]. São Paulo: Hucitec; 2013.

[28] Bader JD, Shugars DA. What do we know about how dentists make caries-related treatment decisions? Community Dent Oral Epidemiol. 1997;25(1):97–103. 10.1111/j.1600-0528.1997.tb00905.x 9088698

[29] Baelum V, Heidmann J, Nyvad B. Dental caries paradigms in diagnosis and diagnostic research. Eur J Oral Sci. 2006;114(4):263–77. 10.1111/j.1600-0722.2006.00383.x 16911097

[30] Kidd E, Fejerskov O. Caries control in health service practice. Prim Dent J. 2013;2(3):4 10.1308/205016813807440010 24340489

[31] Kidd E, Fejerskov O. Changing concepts in cariology: forty years on. Dent Update. 2013;40(4):277–8, 280–2, 285–6. 10.12968/denu.2013.40.4.277 23829008

[32] Fejerskov O. Changing paradigms in concepts on dental caries: consequences for oral health care. Caries Res. 2004;38(3):182–91. 10.1159/000077753 15153687

[33] Renshaw J. After the first 125 years of the BDJ where might clinical dentistry be heading? Br Dent J. 2005;199(6):331–7. 10.1038/sj.bdj.4812692 16184104

[34] Bottenberg P, Ricketts DN, Van Loveren C, Rahiotis C, Schulte AG. Decision-making and preventive non-surgical therapy in the context of a European Core Curriculum in Cariology. Eur J Dent Educ. 2011;15(1):32–9. 10.1111/j.1600-0579.2011.00712.x 22023544

[35] Clarkson JE, Turner S, Grimshaw JM, Ramsay CR, Johnston M, Scott A, et al Changing clinicians’ behavior: a randomized controlled trial of fees and education. J Dent Res. 2008;87(7):640–4. 10.1177/154405910808700701 18573983

[36] Pezzato LM, L’Abbate S, Botazzo C. The production of micro-policies in the work process in oral health: a socio-analytical approach. Cien Saude Colet. 2013;18(7):2095–104. 10.1590/S1413-81232013000700025 23827914

[37] Barros RS, Botazzo C. Subjectivity and a clinical approach in primary healthcare: narratives, life histories and social reality. Cien Saude Colet. 2011;16(11):4337–48. 10.1590/S1413-81232011001200006 22124814

[38] Carvalho JC, Dige I, Machiulskiene V, Qvist V, Bakhshandeh A, Fatturi-Parolo C, et al Occlusal Caries: Biological Approach for Its Diagnosis and Management. Caries Res. 2016;50(6):527–42. 10.1159/000448662 27658123

[39] Maltz M, Jardim JJ, Mestrinho HD, Yamaguti PM, Podestá K, Moura MS, et al Partial removal of carious dentine: a multicenter randomized controlled trial and 18-month follow-up results. Caries Res. 2013;47(2):103–9. 10.1159/000344013 23207420

[40] Schwendicke F, Frencken JE, Bjørndal L, Maltz M, Manton DJ, Ricketts D, et al Managing Carious Lesions: Consensus Recommendations on Carious Tissue Removal. Adv Dent Res. 2016;28(2):58–67. 10.1177/0022034516639271 27099358

[41] Saekel R. China’s Oral Care System in Transition: Lessons to be Learned from Germany. Int J Oral Sci. 2010;2(3):158–176. 10.4248/IJOS10054 21125794 PMC3475601

[42] Haden NK, Hendricson WD, Kassebaum DK, Ranney RR, Weinstein G, Anderson EL, et al Curriculum change in dental education, 2003–09. J Dent Educ. 2010;74(5):539–57. 20446373

[43] Leme PA, Pereira AC, Meneghim MC, Mialhe FL. Undergraduate dental students’ perspectives about experiences in primary care for their education in the field of health. Cien Saude Colet. 2015;20(4):1255–65. 10.1590/1413-81232015204.00812014 25923636

