# Supplementary material for: How Brazilian dentists work within a new community care context? A qualitative study
Source: PLoS One. 2019 May 8;14(5):e0216640. doi: 10.1371/journal.pone.0216640 (PMC6505932; doi:10.1371/journal.pone.0216640)
Supplement: S2 File — Complete transcript of interviews presented in Portuguese. (DOCX) [file pone.0216640.s002.docx]

**Interviews transcriptions in Portuguese**

**ENTREVISTA 1**

P: Bom, a gente trabalha com clínica todo dia, a gente trabalha necessariamente com cárie. Essa entrevista é justamente sobre isso, como você se sente, o que você pensa, como que é sua experiência aqui no (hidden) com o tratamento de cárie?

E: Realmente assim, é o que a gente mais encontra é cárie, doença periodontal a gente não vê muito, problemas oclusais, também... Também tem crescido mas não é tanto, tenho visto bastante também... É... O que eu percebo é que eu não con... Tenho dificuldade que as pess... Os pacientes é, percebam que eles tem que mudar alguma coisa na rotina deles pra não ficarem mais doentes. Eles acham que... A cárie é aquele buraco, e eu venho aqui e... A dentista vai tampar o meu buraco. E sarou... Eles não entendem a cárie como uma doença crônica, multifatorial, não consigo fazê-los entender isso, às vezes é o que eu passo aqui...

P: Aqueles pacientes, a gente sempre tem aqueles pacientes que... Eles retornam, eles sempre retornam com cáries novas, com relação a esses pacientes, como que você pensa sobre isso, como você se sente sobre esses casos?

E: Isso é uma frustração né, você se sente frustrado, que que eu vou fazer pra esse paciente entender, vou desenhar que que ele tem que fazer? Não adianta. Ele assim, tenho sim, e assim, isso é a sensação de fru- sentimento de frustração. Você não conseguiu que ele entendesse o que ele precisa fazer. Nós temos uns 4 assim aqui. (risos) Difícil.

P: E como é que são esses 4, como é que são esses casos?

E:Eles são, é, eu acho, por mim é uma coisa até interessante, porque assim, o paciente ele vem sempre, eles não falt- normalmente assim, não é, aqueles que faltam muito em consulta eles vêm... Depois na época de retorno eles vêêêm de novo na época certinha, eles vêm na época certa, só que você percebe que chega aqui sem escovar os dentes... Muitas vezes você vê que tá aquela placa madura, meio amarelada, então quer dizer, também não tá alterando a dieta... Daí eu expliiico, eu faaaalo, tem alguns que até admitem que não mudaram, mas tem outros que juram de pé junto pra mim que mudou, que não gosta de doce, que escova os dentes, com uma tonelaaaaada de placa... Eu nem evidencio mais, porque não adianta né... Não tá dando certo né, é... Depois de 6 meses eu até fico pensando, não, não é possível, não é uma cárie nova, é a mesma, caiu a restauração... Aí eu olho no odontograma e não, é cárie nova mesmo. Apareceu em muito pouco tempo, mas também ele não muda nada... Não sei, isso daí é um problema.

P: Difícil, a gente às vezes não entende direito o que acontece com eles né...

E:Ah eu acho assim que eu até entendo o que acontece com eles. Assim, a gente precisa tentar conscientizá-los, eu acho que eles vivem numa comunidade que eles não tem perspectiva de melhora. Onde a boca, ela não é a prioridade deles. Eles tem outras prioridades. E acabam não priorizando isso. Então acho que é questão de prioridades deles mesmo. Muitos eu sei que não tem escova, né, toda vez que vem eu dou escova, que a criança, uma criança da mesma família fala que a mãe joga a escova dele fora, eu dou outra... Daí vem o irmão aqui maior que é quem tá com esse problema, a gente sabe que tem muitas... Dificuldades, mas assim, eu não sei como... Vencer essa barreira, fazer com que eles entendam isso né, conscientizá-los da importância.

P: E você comentou um pouco sobre essa aplicação de medida preventivas que a gente usa né, como orientação de higiene, de dieta, né... O que você pensa sobre elas, como é sua experiência com isso em específico?

E: É... Eu já trabalhei isso em grupo, já trabalhei, é, individualmente... Eu prefiro individualmente. O paciente presta mais atenção. Quando você fala em grupo ele, é, como se tivesse falando com ele, ele olha, disfarça, "nossa, mas tem gente que faz isso?" e daí fala assim, nossa, mas esse é ele (risos), mas tudo bem. E, não consigo muito prender muito a atenção deles nem fazer que eles entendam o que a gente tá falando. E, até na escola às vezes é um pouco difícil também isso. Agora, individualmente, no consultório, eu gosto de fazer isso normalmente na primeira consulta, eu converso mais com eles. Eu percebo assim que, uma parte, até... É... Presta atenção, pergunta, tem alguns que já vem com dúvida, tipo de escooova, enxaguatóóório, quer saber tipo de pasta de deeeente, tem alguns que são bem interessados e você vê que prestam uma atenção mesmo e que realmente... Incorporam muito que você falou, que eu falo, porque você percebe que depois, nos ret-nos retornos a pessoa mudou mesmo, você percebe que ajudou. Uma parte. A outra parte... Mais ou menos, que fica naquele meião, e tem essa parte que não é a maioria, que realmente parece que não absorveu nada, mesmo individualmente.

P: Tem uma faixa do meio que você falou que...

E: É, nã-não sei o quanto é essa porcentagem, mas tem aquele meião que absorve um pouco, que... Tá dentro da normalidade porque eu acho que até em consultório particular eu acho que... Não acabam absorvendo muito. Mas assim, tem uma minoria que assimila muito e que realmente você percebe mudança e a outra minoria que realmente não... Não muda nada, parece que você não falou nada e o que você tá falando às vezes até minha auxiliar, depois comenta comigo que ela fica nervosa, porque às vezes, eu estou conversando com o paciente sentado aqui e ele tá olhando pra lá... Tá olhando pro ventiladooor, pra luz, então ele ele não tá escutando naaaada. Não tem como você falar (estalando os dedos) olha aqui (risos), daí assim, vou fazer o quê?

P: E a auxilar acha isso curioso?

E: Não, ela fica com muita raiva! (risos). É, porque eles não entendem realmente a doença, eles acham que é fechar um buraco, então aquilo tudo, pra eles é blablablá, que eu fico falando, não... Não vão mudar, é essa sensação que eu tenho.

P: E como é que você vê eles lidarem com a própria doença? A própria doença de cárie, como você vê eles lidando?

E: (silêncio, suspiro) Olha... Eu acho que é com negligência. Que a partir do momento que, como qualquer outra doença, por exemplo uma doença crônica, diabetes, eu sei que que eu preciso fazer, preciso tomar medicação, preciso mudar minha dieta, preciso emagrecer, preciso fazer atividade física... Eu vou tentar fazer tudo isso que o médico, segundo o médico falou, direitinho. Agora, é, eu percebo que... Tudo que você pede pra eles mudarem, pra que eles tenham uma saúde melhor, eles não, não fazem, então eu vejo isso como negligência. Eles negligenciam a saúde bucal, talvez até pela questão que isso não é uma prioridade pra eles.

P: O que você tava falado no começo, né... E, sobre o trabalho de você aconselhar e orientar os pacientes, explicação mesmo, relativos à cárie né, como que é sua experiência com isso aqui?

E: Hummm... Não foi isso que eu acabei de falar?

P: Você comentou um pouquinho no começo, mas eu queria aprofundar um pouco nesse aspecto de...

E: O que você queria saber mais específico?

P: Você falou que alguns pacientes, hum, tem uma faixa que absorve tudo, uma faixa maior que é um pouco mais ou menos, e uma outra que é nada assim né, quase nada. É, como que você se sente com relação à isso, é tranquilo pra você hoje no momento, isso te incomoda, isso mexe alguma coisa com você ou é tranquilo?

E: Não, tranquilo nunca é né... O profissional que sabe que que tá acontecendo, o profissional que sabe as consequências disso, que é comprometido, claro que você nunca vai ficar é, é... Tranquilo em relação à isso, é uma coisa que sempre gera uma angústia, sempre uma necessidade de mudança, e sempre tento mudar, ver o que que eu posso fazer de diferente, o que que eu tenho que... Como, é... Eu sempre penso como eu mudar de forma que o que eu falo... Seja compreensível pra eles, que eles entendem, que eles captem aquilo, que aquilo... Inserir aquilo na rotina. Mas ainda não achei essa fórmula mágica. Mas sempre vou pensando, já teve épocas que quem fazia essa orientação era minha auxiliar, teve épocas que era eu, forma que eu vi que fica um pouquinho melhor é quando eu mesma falo com eles. Em grupo, coletivo, não percebi, individual, desculpa, percebi que individualmente parece que é melhor... Agora, é... É um desafio né, (inint) não sei, ainda não descobri como fazer... Como falei brincando, no começo, assim, tem uns que você tá quase desenhando já pra eles, pra ver se eles entendem, claro que não é assim né, só a gente está exagerando... Mas, é... Isso gera incômodo, isso gera uma angústia, uma insatisfação, uma... Às vezes dúvidas, por que que eu não consigo... É... Que eles entendam o que eu falo, que isso não é importante pra eles... Mas tento conviver com isso mas sempre assim, a gente sempre se questiona, sempre tento fazer uma autorreflexão pra, tentar melhorar isso.

P: Tiveram alguns colegas seus, e muito por isso a gente entra nessa questão, que eles comentam que... Que assim, no passado era diferente, no presente eu já lido de um jeito diferente com isso, não me mexe tanto assim, mexia mais comigo no passado e hoje não mexe tanto, com relação à isso você percebeu alguma diferença? Quando se formou pro presente?

E: Olha... Tô demorando porque faz tempo (rsrs)... É... Eu acho que... Não, eu acho que não. O que que mudou foi minha expectativa com relação ao comportamento do paciente. Eu sei que não adianta eu ter grandes expectativas, eu vou falar pra todos, de uma forma parecida, de acordo com a idade, de acordo com a cultura, mas assim, a minha expectativa de tudo que eles vão absorver e do que eles vão fazer não é mais a mesma de antes. Porque antes eu tinha a expectativa de que tudo que eu falava ia ser absorvido, eles iam mudar tudo eles iam inserir todos aqueles hábitos, ia ser as mil maravilhas, a expectativa, agora, é... Se isso me incomo... Incomoda, igual. Por que, porque eu quero que realmente, é uma coisa, é, que... Se você for pensar bem, é uma doença que não é uma coisa tão difícil de se controlar. Em países de primeiro mundo, se a gente ver a expectativa, é, a prevalência de cárie, é super baixa. Mesmo no Brasil é muito baixa... Então não é uma coisa de outro mundo hoje em dia, tão difícil de se conseguir controlar. Então assim, a minha, o meu, mexe comigo do mesmo jeito de quando eu me formei. A expectativa mudou, mas é... Me incomoda. O dia que deixar de incomodar eu não sei se eu seria uma boa dentista. Porque, você concorda, que se o dentista é comprometido com o trabalho dele, e o maior problema que ele encontra no trabalho dele não incomoda mais... O que que ele vai fazer pra melhorar isso, pra mudar? Mesmo porque, a gente tem, a gente não pode esquecer que a gente trabalha com seres humanos, e se a gente, e se as coisas não incomodarem a gente... Seria como trabalhar com máquinas, não é assim também, não sei se eu entendi sua pergunta, então...

P: No dia a dia também aparecem alguns casos que... Casos de pessoas que marcam a gente, com relação ao tratamento de cárie, casos de cárie mesmo, eu gostaria que você me contasse de algum caso que te marcou bastante com relação à cárie?

E: Ah... Duas coisas assim que eu sempre marco... A primeira nem é tanto assim, claro, foi um tratamento de cárie, mas foi uma menina que... Tinha uma cárie grande no dente 36, precisou fazer o canal, ela foi, fez o canal, linda e maravilhosa... Quando ela voltou pra fazer a restauração definitivo o dente tinha tido uma fratura vertical que não tinha como salvar o dente, e quando eu falei pra essa menina, pra mãe dela que veio junto que, infelizmente ela ia perder o dente, porque, com aquela fratura vertical não dava pra salvar mais, o dente... Essa menina chorou. E eu... É... Foi o único caso que eu vi, trabalhan... Faz 6 anos que eu trabalho aqui, de um paciente que se incomodou tanto com a perda de um dente, com a saúde dele, com a saúde bucal, a maioria das pessoas não tem essa sensaç... Isso me incomoda também, porque eu, eu, se fosse eu, eu teria chorado também, ainda mais também que não foi que ela negligenciou, ela foi, ela fez o canal, ela voltou pra restaurar, é que o dente tava muito friável realmente, mas normalmente aqui, acontecem as coisas, eles perdem um dente como quem... Perde a hora pra ir em algum lugar, pra ir à festa, é uma coisa assim, "ah, perdi". E muitas vezes eles ainda, que tem muuito que eles, é, querem tirar todos os dentes pra por dentadura. Nisso... Diminuiu bastante. Mas assim, ainda tem alguns que aparecem com... Porque, pra eles eu não... E quando falam que querem por dentadura é porque não querem mais ter que escovar o dente, então assim, essa-essa menina foi um extremo oposto assim, que não vi isso aqui mais. Então essa marcou bastante... Uma outra coisa que me marcou... Vamos ver o que é importante, não sei se essa aqui está envolvido porque foi uma ocorrência durante o tratamento, vamos ver se teve mais alguma coisa... (silêncio) Uma outra coisa que me marcou foi uma menininha, quando ela veio aqui, de 13, não, desculpa, 3, de 3 anos, ela tinha acabado de vir, não lembro o lugar mas era de uma outra cidade que não sei se era de minas ou do Nordeste, e que quando ela chegou aqui eu quase chorei porque a menina tinha vi- os viiinte dentes decíduos cárie de raíz residual, no toco, e a mãe veio reclamando que a criança tava com muita dor. Eu imagino, porque ela tinha 3 anos... E aquela situação, eu fiquei um pouco, é... Me senti assim de mãos atadas, o que eu pude fazer eu falei, o que eu acho que vai ser de melhor pra essa criança vai ser eu encaminhar ela pra odontopediatria na (HIDDEN), que eu acho que vão conseguir uma reabilitação pra ela e um tratamento menos traumático, porque eram 20 dentes, e isso, até hoje, quando eu vou fazer palestra de gestantes, que eu vou conversar com elas sobre a importância da, da, de não inserir hábitos, por exemplo, é, açúcar na mamadeira, é, evitar alimentos açucarados muito ceeeedo, o hábito da higiene que é boni- que é, importante, da higiene bucaaaal, desde cedo, eu reforço esse caso. Eu... A única pena que eu tenho é de não ter tirado uma foto, pra ver, mas eu acho que até deve existir alguma coisa na literatura assim, mas é... Foi bem impactante isso pra mim, e veio, ela não era de (hidden), veio do nordeste então assim, não sei como que eram as condições dela... Mas foi, mexeu bastante.

P: Você chegou a ver depois?

E: Não cheguei a ver depois porque ela, é... Não era do meu posto. Ela era do posto que é a unidade espelho, que é o posto Serra Verde, então com os pacientes de lá eu tenho menos esse contato como a gente tem com esses daqui. Posso até procurar saber com a agente de saúde, porque foi através dele que eu atendi essa criança, que me assutou, ele falou pra mim que tinha acabado de chegar, ele foi visitar, fazer uma visita na casa, chegou e viu essa criança. Daí ele trouxe pra mim, então posso até ver com ele, ele pode lembrar.

P: Eles mudam muito também né, às vezes pode não estar mais morando lá

E: É, pode ser, porque já, foi bem no começo, já tem uns 5 anos essa história...

P: É, aqueles casos de livro né, que você vê de cárie... Nesse caso ela veio com a mãe?

E: Foi a mãe. Se não me engano era a mãe e a avó.

P: E como que eram elas nesse contexto aí, elas tavam muito preocupadas ou, foi a dor que trouxe, como que foi isso?

E: Na verdade, elas assim... Eu não sei se era tudo novo aqui né, diferente do lugar onde elas moravam, mas na verdade quem percebeu a necessidade da criança vir no dentista foi o agente de saúde. Não foram elas. Não foram elas que falaram alguma coisa, então... Não sei, inclusive ele também veio com ela nesse dia da consulta, não só as duas, o agente comunitário. É... E elas só se queixaram que a criança sentia muita dor. E eu expliquei tudo a importância, porque fiquei com medo de não levarem, como era recente no município, de não levarem na (HIDDEN) pq é um pouco fora de mão pra eles realmente. E... Mas eu acho que acabaram levando, elas entenderam, daí d-deu pra conversar, elas entenderam direito a importância, é, mas... Eu não achei, a princípio, que estavam tão preocupadas assim com a criança não. Com essas, c-com a condição da criança. Não que fosse um caso de, descuido, de descaso, mas eu acho que pra eles aquilo era... Comum. Acontecia e era dente de leite...

P: E o aspecto geral da criança, tava bem vestida, em termos de saúde geral tinha mais alguma coisa que marcava mais?

E: Não... Não, nada de diferente, c-como eu falei pra eles, né, não valorizam muito a saúd...É, que eu imagino que não valorizam, a criança não era uma criança que veio suuuuja, não era uma criança que tava muito maaaagra... Assim, você via que não era uma família que, com, bastante dinheeeeiro, que ia tá com uma roupinha nova, não, mas era assim, dentro do contexto tava assim, razoável...

P: Tem algo mais a comentar sobre o tema?

E: Não, eu acho que é isso mesmo, porque assim, realmente é um desafio, acho que esse desafio não é só meu, deve ter sido desde os primeiros profissionais que pensaram sobre isso e como melhorar isso, e que é uma batalha que, a gente apesar de ficar desanimado, apesar de ficar frustrado, a gente tem que que sempre lembrar que não, a gente tem que continuar, porque se tem aquela minoria também que não escuta e que não muda, tem aquela parcela que vai escutar e que vai mudar e que aquilo que você falou serviu pra ela, mudou a... A, o comportamento e melhorou a vida dela, então você nunca sabe quem que é o paciente que vai escutar e quem que é o paciente que vai ignorar. Então assim, é a gente, mesmo dando esse sentimento de frustração a gente não pode desistir nunca de batalhar, de lutar, de incentivar... De insistir e de orientar. E de sempre tentar formas diferentes até a form- a hora que o paciente consiga entender o que o profissional não ficava tão distante do paciente, que o que a gente fala seja uma coisa... É... Compreensível pra eles, acho que é o desafio de todo profissional comprometido, se tá comprometido com o seu trabalho você vai continuar insistindo nisso. Mas eu acho que é isso, um desafio de sempre, sempre, sempre que existir a doença cárie vai ter isso.

P: São tão legais aqueles casos que valem a pena né...

E: Mas mesmo os que parece que não valem a pena, às vezes eu continuo. Tem uma desses, mais ou menos, desses 4 casos, que toda vez que ela vem, eu falo, fa-daí eu já falo assim pra ela "olha, você já sabe o que que eu vou te falar, não sabe?" Então, mas eu preci-não fica, eu já falo, "não fica brava comigo, mas eu preciiiso falar" (risos), de tantas vezes que eu já falei. É... Nem desse eu desisto (risos) fica aquela situação né, a gente pensa um pouquinho, se, ai gente, é minha obrigação falar, mas eu tenho certeza que ela não vai escutar nada (risos)... Infelizmente isso a gente pensa.

**ENTREVISTA 2**

P: Queria que você me contasse, suas idéias pessoais a respeito do trabalho com os pacientes que tem cárie no dia a dia, como você se sente, o que você pensa, como é sua experiência aqui?

E: Como, (hidden)?

P: Como que é pra você trabalhar sobre a cárie, com seus pacientes aqui?

E: Ah eu não sei se eu entendi muito bem a pergunta, mas pra mim... Pra mim é um trabalho sem fim. Pros pacientes daqui. Entendeu? Percebo que pros pacientes deste lugar... Pra mim no consultório era diferente. Pra mim, pros pacientes daqui é um trabalho sem fim. E também não muito específico de padrão de distribuição, tem gente que chega com muita cárie, tem gente que tem uma condição péssima de higiene e não tem nenhuma cárie, então... Mais por causa do que a gente já sabe de como a cárie acontece mesmo. Mas pra mim eu não vejo fim.

P: E como é essa história do sem fim... Como que é isso?

E: Eu acho que... A população daqui tem um perfil que me leva a acreditar que eu sempre vou ter... Tem dia que eu atendo 14 pacientes e nos 14 eu faço restauração... Eu acho que nunca vai acabar de ter cárie... Assim, por mais que a odontologia esteja (risos), com todo conhecimento que tem, que a gente sabe, e que as vezes até aqui em (hidden) mesmo, comparando uma região e a outra, aqui a gente chega a fazer um monte de extração dentária por causa de cárie... Coisa que as vezes não chega nem na (hidden) pra fazer... Né, então dentro da mesma cidade, região diferente, no consultório mal chegava uma coisa dessa pra mim, assim, era muito atípico chegar um dente pra extrair porque perdeu por cárie, aqui...

P: Era diferente então no consultório e... O contexto atual aqui?

E: É, muito. As coisas que aparecem aqui são muito diferentes. E eu nunca vi tanta cárie e tanta perda de dente por causa de cárie também... As vezes em paciente jovem até... E hoje em dia tem muito recurso, né... A escola tem atendimento desde criança, todos os projetos que tem aqui, tanto em parceria com a (hidden), todo mundo tem informação e mesmo assim o negócio continua acontecendo, sabe... Então, pode ser por uma questão de hábito, de dificuldade de mudar os hábitos, alimentação né, condição de higiene, mas... Eu não vejo fim. Eu percebo diferença por exemplo, em tratamentos... É, pode ser pelo perfil socioeconômico mesmo, porque eu já cheguei a cobrir um outro bairro, férias do outro dentista, as pessoas que chegavam pra mim lá, tinham um perfil socioeconômico um pouco melhor, um outro padrão, então eles já chegavam no consultório pra mim com todos os dentes restaurados com resina... Então pra mim era muito complicado tentar fazer um exame clínico e tentar avaliar cada resina pra ver se tá boa ou se não tá, se tem que trocar, se tem que recontornar porque nooormalmente não tá. Né... Difícil você ver uma boca que todas tão perfeitas e você não precisa fazer nada... Então, tecnicamente assim, de execução, como foi feito... Então lá era muuito complicado. Aqui é mais fácil pra mim porque são poucos os que chegam assim. A maioria já vem com a cárie, panela mesmo, e pra mim é mais fácil então (risos) identificar se tem cárie ou se não tem. Mas é muita... Ainda mais agora com a agenda aberta, que a gente recebe muito paciente, a gente atende muita gente, então você vê que o... O padrão perpetua assim, todos eles assim, sabe, todos eles tem... Assim, eu não vejo que vai... Eu não sinto que vai chegar um dia que eu vou atender paciente que vai chegar aqui com os dentes com pouca cárie.

P: E essa agenda aberta você achou que mudou alguma coisa no sentido de interferir no... No que acontece no dia a dia em termos de terapêutica, como é que foi essa mudança?

E: Pra gente... Pra mim particularmente tá mais corrido né... Porque... Na verdade a gente continua atendendo o mesmo número de paciente que a gente já atendia antes, só que antes a gente continuava o mesmo paciente até ele acabar antes de entrar um novo, hoje em dia tem novo entrando toda hora, então você não consegue acabar os que você já começou antes de começar os novos, então é muuita gente, tudo misturado, quando o paciente chega aqui, como ele vem depois de muito tempo no retorno, você acaba nem lembrando mais dele, do que é, tudo só olhando no prontuário que eu lembro do paciente... E... O volume em termos de cárie é muito maior, entendeu? A gente tá falando de cárie, a gente só vê cárie. Cada vez mais.

P: Então você achou que essa mudada na demanda deu uma interferida nesse sentido?

E: Deu. Deu porque apareceu mais né... Agora a gente andou mudando algumas regras em relação às faltas, a agenda tá começando a tomar um rumo melhor e os retornos tão ficando pra dentro do mesmo mês, porque antes isso não acontecia, ficava um mês e meio, dois, para retorno. (9:15) Agora tá começando a ficar com uns 20 dias, 15 dias, então agora eu tô vendo o paciente mais rápido e tô ten... Tô conseguindo a ter um controle melhor daquele paciente pra terminar. Então tá ficando melhor... Mas o que acontece em termos de cárie, de perda de dente por causa disso, no mundo de hoje isso não deveria mais acontecer, e aqui acontece muito. Uma coisa que você acha que é só aquele pessoal que morava no sítio que não tinha informação nem escova de dente que ia acontecer.

P: Não é bem assim, né?

E: Não é....

P: E você acha que em termos de terapêutica preventiva, isso mudou também um pouco?

E: Tô aqui a 8 anos... Você tá perguntando se mudou a terapêutica preventiva dentro do consultório, ou no geral?

P: Ah no geral, no geral...

E: Acho que em 8 anos a condição... Pra mim, quando eu cheguei aqui há 8 anos eu extrairia muito mais dente por causa de cárie do que eu ex... traio hoje. Mesmo ainda tendo muito pra fazer. Então há 8 anos no bairro, eu percebo um pooouco de melhora, mas sem dado científico e de registrado, só de observação. Eu percebo um pouco de melhora, mas a dificuldade que eu tenho aqui em comparar duas datas é porque a população não é a mesma. A população aqui é muito flutuante e toda hora eu recebo paciente novo de outros estados, principalmente do nordeste... Então quando volta o meu pacientinho que eu já tratei, normalmente é só uma manutenção mesmo e raramente ele tem que fazer outra restauração, agora quando eu recebo esse monte de paciente novo, é tudo gente de fora, que veio de outros estados, tão morando aqui há pouco tempo e nunca passaram por dentista aqui, então daí por isso que acho que o trabalho não (risos) acaba também, porque... A população que a gente atende de 8 anos de agora, ela só cresce e cresce com gente nova e... Então a gente não tem como...

P: Parece que não tem fim?

E: Não tem fim, porque vem gente de todo lugar então não dá pra saber se o trabalho que a gente tá fazendo aqui tá tendo resultado, porque a gente acaba pegando pessoas que não tiveram acesso a essa parte preventiva na escola. Você pensa, uma criança de 11 anos que recebia palestra, brincadeira, flúor na escola, hoje tá com, sei lá, 18, 19... Só que como eles mudam toda hora, você não consegue ver se isso teve impacto, até mesmo pra levar pra dentro das casas, então eu sinto assim, acho que não tem como traçar um... Como fala... Não tem como a gente enxergar muito, como aconteceu por conta do tanto de gente nova que chega toda hora, inclusive de outros bairros e de outras cidades também, outros estados, então... Não dá... (12:15)

P: P, e naqueles casos em que o paciente o mesmo paciente tem sempre muitas lesões de cárie em retornos, como você se sente com isso? O mesmo paciente que você já viu.

E: Antigamente (risos), quando eu entrei pra trabalhar aqui eu ficava um pouco incoformada né, de ver como o paciente tinha acesso à informação de qualidade, porque não era de qualquer jeito nem qualquer informação, a gente sempre deu muita atenção, sabe? Eu sempre fui muito atenciosa com os pacientes, sempre pra todos, cada um que chegava aqui, eu procurava fazer orientação de escovação, mostrava no espelho, perdia uma sessão só fazendo isso, conversava com eles sobre cárie, sobre doença periodontal... Aquela lavagem cerebral que a gente tenta fazer no paciente mostrando na própria boca dele, pra ver se ele toma jeito e ajuda no tratamento pra ele mesmo. Mas... Com o passar do tempo, e entendendo que esse não é o melhor jeito de mudar... Muda hábito de paciente, de um, de dois, mas não (risos) muda da maioria. Então... Entendendo isso eu fui ficando mais conformada... Em aceitar que eu posso fazer minha parte, mas se ele não quiser fazer a dele, não adianta me descabelar... Então assim, o que eu... Sinto é que eu não fico mais... Mais estressada (risos), se eu vejo que o paciente não tá colaborando com o tratamento. Não é fácil e pelo pouco tempo que a gente tem de consulta com o paciente aqui, eu não tenho com... Eu não tenho tempo mesmo, eu tenho que atender 14 pacientes no dia, eu tenho meia hora pra cada um, se não chegar nenhum encaixe, então em meia hora eu tenho que receber, conversar um pouquinho, colocar na cadeira... Se tiver que fazer o procedimento, desde a anestesia até a finalização tirar pra fora pra limpar a sala, pra poder receber o próximo... Então é muito pouco tempo, eu não tenho como, com todos os 14 que eu atendo no dia, agendados, ficar conversando com eles sobre mudança de hábito, né, então o que eu faço às vezes é que eu perco a paciência com aquele paciente que eu já falei 10 vezes e ele só volta pra minha consulta sem escovar os dentes, cheio de placa, eu começo a falar o seguinte: a gente não vai mais restaurar enquanto essa placa tiver aqui... (risos) Aí enquanto a placa tá lá ele começa a ver que se ele vem na consulta com o dente sem escovar, ele não vai ter a restauração que ele precisa fazer, então aí às vezes eles começam a escovar. Às vezes eu abro mão e faço, pra não perder consulta, mas eu tento fazer desse jeito com eles, mas não todos também, aqueles mais específicos mesmo né, que não... Que vem com aquele moonte mesmo mas... Não ajuda... Mas é difícil...

P: E como você percebe que eles recebem informação?

E: Como eles recebem? Eles não gostam. Eles não gostam mas acabam ficando, às vezes, um pouco... É, constrangidos, e não rebatem muito porque eles sabem... Ah, que eu sou a dentista, ele é o paciente, e eu to tentando dar um tratamento pra ele... E... Eu apenas... Digo que ele tem que ajudar. Que não tem como colocar resina em cima da placa. A gente até criou um projeto aqui, de começar a escovar os dentes do paciente como uma atividade coletiva mesmo. A gente até já instalou espelho no banheiro, alí, pra essa população adulta ter acesso à escovação supervisionada que é só as crianças que tem sempre né...

P: Aí seria caso a caso aqui?

E: É, é que daí todo paciente antes de entrar na consulta, a N (Auxiliar da unidade)... Orientava... Mas é mais uma coisa pra ter acesso ao método... Né... Insistentemente toda consulta... Mas não pra... Garantir que ele tá fazendo em casa do mesmo jeito né.

P: E isso já começou, deu certo, foi pra frente?

E: Tem paciente por exemplo, XXXXX, que eu não dou alta, eu faço voltar todo mês pra escovar. Porque ele não escova...

P: Nesse esquema aí?

E: Na cadeira, pra fazer profilaxia mesmo. Mas aí são alguns pacientes que tem, por exemplo, alguns problemas de saúde grave que chegaram aqui numa situação ruim, e a gente resolveu e eu acompanho eles pra isso não acontecer de novo... Porque senão vai ficar ruim pra eles e pra mim também (risos), porque a dificuldade é que eu tenho pouco recurso aqui pra tratar, e às vezes depende de encaminhamento, então pra evitar que eles tenham um problema eu acabo chamando todo mês. Paciente com deficiencia cognitiva, sabe... Então tem uns que fazem parte de uma listinha que todo mês eles vêm, normalmente com problemas graves de saúde ou uma deficiência de cognição, mas... É.. No contrário, entra como normal, retornos como os outros... Mas esse projeto não tá indo muito pra frente... Por conta de organização nossa, por enquanto. Ele começou, agora tá indo mais ou menos, mas... É correria né? Falta de organização nossa porque às vezes a gente tá com paciente na cadeira tem que lembrar que a Natalia tem que sair, chamar o outro pra colocar lá, pra vim, passar enquanto eu dispenso, então nessa correria às vezes eles se perdem. (17:40)

P: Isso aí tava rolando em paralelo, não interferia na sua agenda, uma coisa a parte?

E: Não, são os pacientes DA agenda. A Natália ia lá, chamava, punha no banheiro pra escovar... Orientava, vinha aqui, terminava o atendimento comigo, daí a gente dispensar, colocar outro, sabe? A gente pensava em extender isso depois pros grupos, pra recepção, mas a gente não consegue se organizar nem com (risos)... Com os que tão na agenda (risos), a gente precisa melhorar como que a gente vai fazer... Porque é muito corrido, sabe? A unidade é muito demandante, então a gente não tem esse tempo pra ficar ahh, pegando paciente, a gente tem que se organizar melhor pra ver como (risos) que vai fazer.

P: Ah mas legal que foi uma idéia de vocês né?

E: É, não, vai funcionar, tem que funcionar, eu trouxe um espelho pra por nesse banheiro aí pra isso, mas... Mas também não vai resolver, né, XXXXX? Pra mim continua sendo um problema de comportamento... E pra mudar... É muito difícil.

P: Como que você vê seus pacientes lidarem com o próprio problema de cárie?

E: Como eu vejo? (silêncio) Acho que eles não tem a dimensão que a gente tem, como profissional, do que é o problema. Eles vêm de uma cultura, de uma criação que... Não valoriza a permanência dos dentes (risos) na boca, e não tô generalizando, é claro que tem as exceções, tem as pessoas que são muito preocupadas, né e tem esse cuidado, mas no geralzão... Eles já tão acostumados que dente dói, tira. Que se perder dente depois põe prótese, então eles não tem aquela coisa de valorizar... Como assim: ah mas seu perder esse dedo, vai me fazer falta? Vai... Porque é um dedo da minha mão, mas o dente da boca não tem problema, não entendo o porquê dessa diferença. Mas... Eles, eles tem já essa cultura de que é assim mesmo, então eles não se, eles não ficam tristes... Se eles perderem um dente. Né, eu acho que, quando eu sonho que eu perco um dente eu quase morro hora que eu acordo de desepero!

P: Você costuma sonhar muito com isso?

E: Eu sonho às vezes, já sonhei que eu perdi todos os dentes várias vezes (risos)... Então pra mim é uma angústia, um desespero sabe, imagina se acontecer uma mancha branca no meu dente eu não vou me conformar... Mas eu sei porquê né, porque eu não vou me conformar, agora eles parecem que mesmo sabendo porquê não incorporam aquilo assim né, de uma forma que... Traga neles esse sentimento de medo de perder e de ter cuidado portanto com aquilo né, então, pra eles... Não toca... Não tocou os avós, não tocou os pais e não toca os filhos também, tanto é que pra eles... Muita gente chega aqui assim, a criança tem cárie em dente de leite não tá preocupado se a criança vai ter dor, se vai inchar, se vai ter uma infecção. Tá preocupada assim "ah mas precisa fazer, vai cair", né? Eles não tem noção de que o dente de leite é igual um dente permanente, assim né, em termos de ter polpa, da dor e abscesso, então...

P: É, eu percebo isso também...

E: Não é fácil (risos)...

P: Pa, como você vê, como você pensa ser a melhor forma da odontologia atuar sobre a cárie, o ideal?

E: (silêncio) Ai, como, por exemplo? Por que você acha como, hoje a gente atua sobre a cárie aqui no bairro fazendo restauração em quem tem cárie e tentando na escola fazendo escovação supervisionada e flúor.

P: É, como que você vê assim, a forma ideal, como se fosse o padrão ouro de intervenção da odontologia sobre o problema?

E: Acho que agora você me pegou porque eu nunca pensei nisso né... Eu precisaria pensar um pouquinho mais pra te responder mas de imediato, sem pensar muito, eu acho que tinha que ter mais propaganda. Eu acho que falta proganda, falta divulgar mais na mídia mesmo, porque é isso que eles tem acesso né. Então o que a gente faz só no consultório, nos trabalhos e tal acho que não causa tanto impacto, agora eu acho que pra tentar mudar um pouco acho que a gente tinha que ter mais propaganda e mais propaganda, é... Mais agressivas, não sei se é bem essa a palavra que eu quero usar mas assim, de maior impacto mesmo, que deixasse o paciente... Com medo. Com medo de ter o problema e portanto ele, ele se cuidaria melhor. Eu acho que falta mais. Porque a gente tem propaganda de pasta de dente mas a gente não tem propaganda... Né, claro que é uma doença como as outras e ninguém fica fazendo propaganda disso, mas com relação à cárie acho que falta mais, no geral, a turma saber o que é, e o que acontece, quais as consequências disso, porque eles não tem noção de que perder um dente... O que que vai acontecer na oclusão, quais são os problemas musculares e de dor que eles podem, eles acham que é simplesmente um dente a menos e ponto final.

P: Você falou da pasta, a impressão é que, você acha que esse tipo de propaganda existe, isso daí atinge mais, né a... Talvez a população?

E: É, porque os pacientes chegam pra mim perguntando que pasta eles usam. E hora que eles me ouvem responder que eles não precisam nem da pasta (risos)... Né, não tô dizendo do flúor com relação à cárie, que é o objetivo da entrevista, mas to dizendo assim, hora que você explica o... O... Como a cárie acontece e qual o papel da pasta de dente, da escova e do fio dental, hora que você fala que se você fizer isso direito nem da pasta você precisa, eles não se conformam assim... Na verdade eles querem remédio, né, querem medicamento, querem cosmético, então eles vêm aqui e me perguntam "mas doutora, qual pasta eu uso?"... Porque eles vêm aqui falando "nossa, meus dentes tão todos estragados, nossa mas eu não sei, qual pasta é melhor pra eu usar (entonação de "descrédito"?) ?" Entendeu? Eles acham que a pasta que eles usam... Que vai fazer eles terem mais ou menos cárie, assim, que vai ajudar a prevenir que eles tenham o problema, e eles não entendem que é o jeito que eles vão... Cuidar que vai prevenir, então eles acham que é a pasta, que é o... Enxaguante bucal... Usa fio dental? Não, mas uso Listerine. Porque a propaganda mostra que o Listerine passa no meio dos dentes levando as bactérias embora (risos). Então eles acham que é isso mesmo (risos), é... Entendeu... E eles vêm aqui e acham que é aquilo, eles esquecem do fio dental, eles vão tudo atrás do enxaguante bucal que é mais fácil.

P: E quando você orienta isso pra eles como que é a reação? Como eles reagem a essa sua informação?

E: É, eles não se... Não é que não se conformam, eles ficam surpresos né, porque eles "nossa, é mesmo, sempre usei o negócio, a televisão me fala que é daquele jeito... Como que não é?" Né? Aí você tem que dar exemplo, tentar mostrar como funciona... Como é que você fala que a televisão tá enganando? Que não é daquele jeito? Eles não acreditam. Eles não acreditam, tenho certeza que eles não acreditam (risos)...

P: Interessante, teve uma outra unidade que eu fui que comentaram essa mesma questão do... Da pasta tal, é bem marcante isso né...

E: É, porque é o que eles vêem na televisão né... Não tem muita propaganda de fio dental, mas da pasta e do enxaguante tem...

P: (comento sobre minha experiência com orientação de enxaguantes)

E: É, quando você fala que não é nem pra usar... Aí eles não se conforma também né, como não é nem pra usar? Nossa, tão bom... Eles não entendem... (26:37)

P: P, em termos mais genéricos, não sei se você pode me falar alguma coisa sobre a relação, não a sua especificamente, mas como que é a relação dentista-paciente, como você senti isso, você já deu alguns pitacos sobre isso...

E: Como assim, XXXXX? Como é minha relação em que sentido?

P: Relação dentista-paciente... Tem alguma coisa que te chama a atenção que seja difícil, ou que te dê mais trabalho...

E: Ah não sei, acho que... A minha relação com o paciente aqui é diferente... Não com alguns né, tem alguns pacientes que são muito do posto. Então você acaba criando um vínculo maior. Tem uns pacientes que só vem pro dentista, e quando vêm entram mudos e saem calados, mesmo você tentando... Nos momentinhos que dá, puxar alguma conversa. Então... Não tem muita proximidade no geral assim... Não tem muito vínculo no geral.

P: E tem uns que você sente que tão sempre por aqui...

E: É, tem uns que são mais amigos do posto. Já acabam conhecendo, conversando, às vezes participou de comissão local de saúde, então já é mais familiarizado com o posto, ou então é gente que traz criança, nenê todo mês, você encontra todo mês, tem gente que você já cria um vínculo maior. Ah, tem gente que não... Mas, também, não pela gente, mas as vezes eles não dão abertura, né, o perfil deles assim de relação interpessoal às vezes dificulta isso, eu tenho gente que entra no meu consultório, calado, não me fala nem bom dia, mas eu acho que é de timidez, não é... Claro que existe uma falta de, de jeito de educar também, da família, mas eu acho que também por eles serem muito timidos, e de repente tarem entrando "aí, é o dentista né", então assim eles entram às vezes sem falar, na hora que eu acabo eles levantam da cadeira antes de eu levantar a cadeira, às vezes eles até me batem aqui porque eles puxam aqui pra levantar, eu levo, tenho sempre que tirar a broca daqui porque senão eu to fazendo alguma coisa eles puxam pra apoiar, o negócio entra em mim! Quase que eu tomei várias vezes uma brocada... Então aí eles já vão pegando o cartão e vão saindo da sala sem eu ter dado as orientações pós procedimento e falado o que ele vai ter que fazer na recepção, de tanta que é a vontade dele de sair daqui, e muitas vezes não é porque é a sala do dentista, é por timidez assim, mas não é a maioria, é um ou outro, só um exemplo...

P: (pergunto se esses pacientes são assim também fora do consultório do dentista)

E: É, mas a maioria não é assim, eu só dei um exemplo de como tem gente assim, tem outros que vêm, sentam, ficam falando da filha, do neto, nãnãnã, contam a vida inteira... E você não tem como dispensar pra não ser mal educada e... Né, tem outro me esperando lá atrasado lá pra ser chamado.

P: Sei, esses que trazem muita coisa acabam atrapalhando o atendimento ou não?

E: É que não são todos, a toda hora. Então não, não atrapalha. Porque a gente também como dentista já vai dando um jeito, já sabe conduzir a situação né... Tem uma hora que você continua falando e ele para (risos)... Tem uma hora que você tem que fazer ele parar e você começa a só você falar né e ahã (risos). Tem uma hora que tem que parar (risos).

P: P, tem alguma experiência que marcou bastante você, casos de pacientes que tem cárie... Marcou bastante assim, que você lembra quando pensa nisso... (30:39)

E: Ah... Especificamente por causa da cárie não, tem experiências que me marcaram por causa das situações que passei aqui com eles né... Em decorrência da cárie né, procedimento, de técnica, de ter passado mal... Mas de cárie? Tem uma, uma menina que ela tinha, acho que uns, nem 20 anos, tinha uns 16, 17... Ela tinha muita placa, uma placa espumosa, assim, que subia, cubria o dente inteiro, você não enxergava dente hora que ela chegava, e ela já tinha perdido vários dentes por conta de cárie, ela era muito nova, não tinha nem 17 anos, tinha essa idade assi, 16, 17... Ela não tinha, você nem enxergava o dente dela, e ela tinha perdido e ela ia perder os que ela tinha também e ela sempre vinha no encaixe comigo, ela não marcava consulta, ela sempre vinha no encaixe querendo atendimento e eu sempre... Resolvia o dente dela num dia, no outro, no terceiro eu falei "não vou mais te atender no encaixe, você vai agendar consulta, porquê... Desde a primeira vez já era orientação e ela só esperava doer pra vim. E aí ela começou a fazer o tratamento mas não tinha o que fizesse aquela menina escovar o dente dela, porque ela falava que escovava pra mim né, mas tava na cara que ela não escovava, tentava escovar, porque... A placa continuava lá e ela, foi passando os anos e ela foi perdendo um, perdendo outro... Hoje em dia, faz tempo que não a vejo porque ela deve ter começado a trabalhar, mas eu imagino que ela deva ter só os dentes da frente na boca dela, se ela tiver. Porque (risos), de perder por placa, por cárie, ela sofrendo... O que a cárie dava pra ela, porque ela tinha dor... Ela sofrendo pra ter que tirar dente, mesmo assim ele continuava lá, sabendo, porque ela chegou a fazer tratamento, a gente começou a conversar, mostramos pra ela, mesmo assim... Ela continuava com o mesmo hábito, porque se a placa continuava lá ela não tinha mudado hábito né... Então ela foi perdendo os dentes, eu tenho certeza que hoje ela tem muito menos dentes do que tinha quando vinha aqui (risos), porque tava acontecendo aqui na nossa frente, ela perdeu um... Perdeu outro, perdeu outro e aí...

P: E dentro do que você conversava com ela, o que pegava mais assim, você sentiu alguma pista do problema?

E: Não... Não, não tinha nada de diferente, porque ela alegava que ela escovava, que ela cuidava, mas acho que era só pra falar mesmo porque tava aqui. Né? A questão da alimentação, chiclete, bala também, eu acho que ela (risos) chupava bastante, também... Porque adolescente, é o perfil, assim... Fica o dia inteiro com o chiclete na boca, né, bala, pirulito... Mas ela aqui não falava, também era daquelas pessoas queitas, que não se abriam muito, então não dava pra ter muito...

P: Não falava muito aqui?

E: Não... Começou o tratamento depois começou faltar, né, daí só vinha em encaixe de novo... Então essa, com relação específica de cárie assim... Essa me lembro bem. (33:46)

Ah e uma outra que eu me lembro também, que eu inclusive atendi hoje, que foi bem chata pra mim foi que, é um bebê... A mãe, grávida, passava com a gente, tinha alguns problemas de cárie na boca, aí ela teve um bebê, ela tinha outros filhos, os filhos com cárie também, e o filho dela eu atendia era só endo que tinha, sabe? Todo dente decíduo dele que eu mexia... Endo. Aí eu já tinha tratado ele, ela teve a menina, a bebê. Aí ela começou a trazer a menina na puericultura e na puericultura a gente atende desde que nasce, todo mês, até fazer dois anos, na verdade até fazer 1 ano, e de 3 em 3 meses até fazer 2. E eu que já encontrava com essa mãe o tempo inteiro no tratamento dela e no tratamento do filho, comecei a encontrar no tratamento da nenê, que era acompanhamento, a nenê nem tinha dente. E eu fazendo as orientações como pra todo mundo, a gente acompanhava, ensinava a limpar a boquinha, falava de chupeta e mamadeira, falava do açúcar e tal, a hora que começou a nascer o dentinha na criança a gente começou a orientar a escovação com a escovinha, porque as mães elas tem a preocupação "e agora, a gente tem que usar a escova, como que é?", normalmente... Ela um belo dia chegou pra mim numa consulta dessas... Porque eu já sempre argumentava muito com ela, porque ela não escovava o dente da criança e ela já tinha aquela história da família inteira com cárie. E ela não escovava direito nenhum dente da criança e eu insistindo com ela que ela tinha que escovar justamente pra não ter dor e abscesso igual o outro filho tinha... Um dia ela chegou na consulta, e a consulta era lá na salinha da pesagem, porque eu fico lá com os meninos e em paralelo ao serviço deles eu fazia o meu ali porque eu não precisava da cadeira... Ela chegou na consulta, eu tava sentada na cadeira, na sala, hora que ela entrou na porta e olhou pra minha cara ela falou assim: "já sei. Não adianta você me falar que eu tenho que escovar o dente dela, que eu não vou escovar. Não adianta você ficar me falando, deixa assim mesmo que quando tiver cárie trata.". A partir daquele dia... Eu não abri mais a boca pra falar nada pra ela... Porque ela não queria! Ela já sabia! Ela mesmo falou "eu já sei"! Eu sei que eu tenho que escovar mas eu não vou... Então hora que tiver cárie, eu vou tratar... E ela nem se preocupou que a filha ia ter dor... Hoje ela traz a menina aqui com cárie pra mim... A menina tá com 4 anos... Os dentes decíduos molares com cárie... Ela às vezes tem dor, e ela já me procuorou um dia desesperada no encaixe dizendo que ela tava com uma "panela", e o que que eu tive que falar pra ela? Você tá escovando? Né? E encaminhei pra tratar lá no ceozinho, porque, vai adiantar vim tratar comigo, não vai! Porque se desde quando era bebezinha, ela não me ouvia pra prevenir a cárie, agora que tem a cárie... Eu vou fechar lá com a "massinha", do jeito que eles falam... E vai continuar vindo pros outros, então minha conduta foi encaminhar pra tratar com outro profissional, pra ver se com outro ela abre os olhos... Mas o outro não chamou, e ela trouxe de novo pra mim... Então eu tenho que ficar atendendo a menina que ela não quis prevenir, que acontecesse a cárie, agora ela tem que vim e ficar pedindo, e toda consulta eu peço pra ela escovar e ela fica quieta... Não fala mais que não vai escovar, porque agora a criança tá com uma panela enorme no dente e acho que ela tá com medo de que perca o resto do dente né... Mas ela também não escova. Mas ela... Não fica um clima chato porque é o perfil dela, não sei se ela com ela, ela não cria esse clima chato com a gente... Mas eu também não fico lembrando ela da história (risos)... Mas eu não esqueço, que a menina hoje vem aqui com cárie, correndo o risco... É que a menina tá com um padrão que tá paralizando a cárie, então acho que ela não tem muita dor... Mas eu não esqueço da história e também não fico jogando na cara da mãe que ela tá assim porque ela não limpou. Eu só peço pra que limpe pra que não aumente (risos), a cárie.

P: E nessas consultas você tem... Embora tenha dado esse problema, você volta no assunto?

E: Tem que voltar, né? Inclusive eu tava até receosa, eu não queria fazer a restauração na menina, porque como a cárie tava paralizada, apesar de grandinha tava paralizada, a minha insistência com ela era assim, olha, pra mãe da menina: "se você escovar o dente dela, esse buraco vai ficar assim, não vai sair disso, só vai sair disso se você não escovar", então pra ela também ter um... Um, compromisso de ir lá, escovar o dente da menina pra aquela cárie não aumentar. Mas, sabendo do perfil dela, eu resolvi pelo menos selar esses dentes até que o CEO chame pra tratar, então ela traz na consulta, faço, oriento de novo, mas esse foi um caso marcante porque ela me falou, eu não vou, a hora que ela tiver cárie, trata. E a menina tá tendo cárie. Entendeu, o que que leva uma mãe a preferir que a criança tenha uma panela no dente, e passe por dor, pelo atendimento do dentista que não é confortável, ficar com a boca aberta tomando anestesia se precisar, numa criança de 4 anos, do que escovar quando ela era bebezinha... Né, então...

P: E pra você é tranquilo lidar com a mãe né, senti que você...

E: Hoje é, uma coisa que eu tive que entender, é que as pessoas elas tem a escolha delas. Que pode ser que não seja a minha. Entõa as vezes por mais que eu escolha, é autonomia que ela tem também, entendeu? Então por mais que eu escolha cuidar dela, ela tem que escolher se ela quer ser cuidada. E por mais que seja triste, a escolha dela, é a dela. Entendeu? Então tem hora que não tem como se descabelar, a escolha é dela, ela quis assim, o que eu puder fazer pra ajudar eu vou fazer, mas quem vai determinar se o negócio vai dar certo ou não é ela. Então hoje eu entendo isso, no começo eu não entendia muito não (risos), eu achava um absurdo, eu ficava "nossa, como que pode", eu não me conformava, hoje em dia eu sou mais conformada com essa autonomia que o outro tem com relação ao tratamento dele, né? Então a gente tá falando de um dente que as vezes não vai matar ninguém por causa disso, apesar de ser muito importante, mas tem paciente de câncer que resolve que não quer tratar porque, vai fazer o que?

P: Você falou que você, no começo, pensava um pouco diferente... Isso mexia mais com você?

E: Mexia, eu me desgastava muito mais. Eu me desgastava muito mais tentando, tentando tratar o paciente. Hoje eu já não tenho mais esse perfil. Sabe, não tenho mais, eu tô mais sossegada, (risos), com relação à isso.

P:Pergunto se tem mais algo

E: Hoje eu penso que o jeito que a gente trata paciente não é o melhor. Não sei se eu tenho a fórmula né, pra responder a outra pergunta... Eu acho que não tem uma fórmula, tem que ser muito bem pensado né, pra propor uma coisa dessa, eu não pensei muito sobre o assunto, mas eu acho que o jeito que a gente faz hoje, de, de... Fluoretar a água, tentar levar o flúor de maneira tópica nas escolas depois, né, aqui em (hidden) mais específico né, porque nas outras cidades não sei se tem tanto isso do projeto nas escolas, da fluorterapia em grupo, essas coisas né... Mas eu também, os gestores podem falar né, a própria (HIDDEN) que sempre conduz essas pesquisas pode falar melhor que eu se tá dando certo ou não porque eles tem dados pra isso, eu não tenho na minha mão agora. E apesar, às vezes, dos trabalhos mostrarem as evidências que ao longo dos anos isso tá melhorando né, e tá mesmo... Mas aqui, particularmente, lidando todo dia com essa situação desse território em específico, eu não acho que funciona. A gente não vê melhora. Pode ter melhora em alguma idade, né, por conta dos trabalhos... É, por causa dos trabalhos preventivos, aplicação do flúor, tal, nas sociedades específicas que normalmente se fazem as pesquisas né, idade índice... Mas eu não consigo ver que tá... Que essa forma de trabalhar vai criar um sentimento na pessoa e fazer ela entender que é importante escovar o dente, e que quando adulto ela não vai mais ter problema. Vai incorporar nela o jeito de cuidar e portanto quando adulto ele vai ter menos problemas. Eu acho que vai ser a mesma coisa. Mas eu to no achismo né...

P: Mas é o que você sente né, sua percepção é essa...

E: É, eu acho que fazer o que a gente faz hoje, tanto do tratamento da cadeira como... O da escola né, porque as crianças é que vão levar, né, pra depois (43:41), mas é uma coisa pontual, que não tem sequência, então eu acho que vai ficar nisso e eu não sei se as vezes a gente tá evitando cárie numa idade, qual o impacto econômico que isso tem também e do bem estar da pessoa, se naquela idade ele tá mas mais pra frente ele vai ter, porque mais pra frente não vai estar sobre nenhuma dessas atividades, e o comportamento vai tá o mesmo e essas atividades não vão ter mudado o comportamento da pessoa, e aí ela vai ter depois então, não vai ter criança mas vai ter adulta... Entendeu? Então o que eu sinto é isso...

P: Aí o adulto chega no consultório e também não tem muito o que mudar...

E: Não tem... Dessa maneira, com esse tempo que a gente tem, e com essas técnicas que a gente usa, não. Então eu não sei se isso... Não acredito... Meu sentimento, sem ler trabalho científico falando do antes e depois, porque eu imagino também que os trabalhos sempre dão trabalhos positivos (risos), tem que dar, né? Mas eu não acho...

P: Positivo nesse sentido de... De mudar alguma coisa?

E: Ter diminuido cárie, mancha branca... Claro, você tá fazendo lá atividade de escovação, enquanto tá fazendo... Mas acho que as crianças saem da escola e elas não levam aquilo, pra casa... É só atividade que fez na escola e ponto final, entendeu? Elas não levam nem o conhecimento que a gente passa por elas na escola, nem o... Nem o... Nem a... O, como fala? O resultado daquela ação se deu certo naquele momento não vai pra depois... O que eu sinto é isso, o que eu acho né. Então acho que fica por ali, a gente vai gastando esforço, esforço, pensando num negócio e não tá acontecendo. Acho também que as pessoas ficam as vezes muito preocupadas com o... A questão do flúor e da cárie, sabe, acho que as pessoas acham que o flúor faz milagre, né? Principalmente até as próprias pessoas que não são dentistas, mas que tem um pouco de conhecimento, principalmente mãe de criança né, essa história "ahh, Associação Brasileira de Pediatria recomenda flúor na pasta de dente desde o primeiro dentinho", a turma fica desesperada em usar pasta com flúor nas crianças porque recomendou, médico também recomenda, eu vou no pediatra, toda consulta ele me pergunta, ele sabe que eu sou dentista! Toda consulta ele me pergunta se eu tô usando pasta com flúor! Eu tenho vontade de perguntar pra ele "você sabe que que o flúor faz? Qual o problema de eu usar uma pasta sem flúor nela?", entendeu? E ele me cobra, se eu to usando ou não eu falo que eu tô e ele não continua o assunto. Mas um dia eu vou continuar... Mas eu acho que a turma fica desesperado em... Né, não tô desmerecendo o flúor, ele tem o papel dele comprovado cientificamente por grandes nomes da odontologia, mas eu tô dizendo assim às vezes o pessoal esquece qual é o papel do flúor, o flúor não vai fazer milagre... E as vezes fica, eu acho que é um jeito da turma tentar controlar isso em massa, mas... Individualmente, às vezes tem que ser analisado cada caso e ver se precisa de tudo aquilo, né... Flúor na água, flúor na pasta, flúor (risos), em todo lugar...

P: E o flúor de consultório, os pacientes comentam...

E: Não... Não... Eu mal uso também no consultório o flúor... Mas os pacientes que comentam é assim, muito raro, alguém perguntar porque já foi no dentista particular e o dentista particular aplicou. Às vezes sem indicação nenhuma, porque às vezes o paciente vem aqui com um monte de cárie que é tudo mancha branca... Né, e... Enfim... Então, às vezes é muito raro perguntar, mas eles não costumam questionar muito os tratamentos.

P: Mas você disse que geralmente é paciente que vem de fora?

E: É, paciente que já foi em dentista particular alguma vez e fazia, e agora a gente tá nessa onda de crise que a turma tá perdendo o convênio né, então a turma que tem convênio normalmente é gente que trabalha em empresa e a empresa dá o convênio, daí a paciente fica desempregada tá vindo porque ela perde o convênio, vem tooodo mundo no posto. E enquanto tinha o convênio pasou pelo procedimento... E eu não sei porque que o convênio fez, se tinha indicação ná época né, mas eles acham que é sempre assim, então... Mas é mutio raro, dá pra contar no dedo os que já fizeram perguntas nesse sentido. Normalmente eles não questionam o tratamento, normalmente na consulta inicial que a gente faz a anamnese, exame clínico eu mostro pra eles, explico que que tem de tratamento pra fazer né, dou umas orientações gerais a respeito das doenças bucais... Explico o que vai ser feito nele e normalmente eles "ah, tudo bem", não costumam questionar muito. Eu esclareço pra eles e normalmente está tudo bem.

P: (comento sobre a limpeza)

E: É, é eles já vem pedindo o que eles querem, né, não esperam, e tem uns que a gente nem faz né, porque... Mas, é... Eles vem sem falar "eu vim fazer uma limpeza", tá, eu já comecei, de tanto que eu ouço isso, eu já comecei até a usar o termo com eles porque eu sei que eles entendem o que é, então eu não explico mais, eu até falo junto: nós vamos fazer remoção da plcaca, mas eu até acabo usando a limpeza junto pra eles entenderem o que é.

P: No começo você...

E: É eu tentava evitar esses termos populares, tentava explicar, com linguagem fácil mas o que a gente ia fazer. Mas... Esses dias (risos), uma paciente grávida caiu uma resturação provisória do dente dela e ela me escreveu no facebook. Ela me achou e ela me escreveu uma mensagem dizendo que ela tava grávida e ela tava preocupada porque a "arbituração caiu do dente"... Ou seja, ela sabe o que é "arbituração", então é assim que eu vou falar com ela (risos) naquele horário, depois eu posso até explicar pra ela, né, como o paciente hoje, deitou, ontem acho, eu fui atender ele, ele deitou na cadeira e fui falar "então hoje nós vamos continuar as restaurações que a gente tá fazendo nos dentes", aí ele perguntou assim "o que 'eisso, doutora?" . Eu falei assim "é tirar a cárie e colocar uma massinha no dente, "ah tá" (risos)... Então eu respondi pra ele que nós vamos tirar a cárie e colocar massinha no buraco. "Ah tá, tá bom... (risos)

P: Às vezes a gente explica e nem passa pela nossa cabeça que ele não entendeu né...

E: É, porque as vezes ele acha, como você já falou, você acha que ele lembra o que é restauração, mas eles não lembram. Então você fala, eles não entendem, ele perguntou... E desse jeito ele entendeu, tava bom... (risso) Não é? Eu sei que assim eles entendem...

**Entrevista 3**

P: Eu queria que você me contasse um pouco, suas idéias pessoais a respeito do trabalho com os pacientes que tem cárie no dia a dia, como você se sente, o que você pensa, como é sua experiência aqui com isso?

E: Hoje em dia, acaba dividindo os pacientes com cárie por geração. Pela idade mesmo. Então hoje os pacientes adolescentes, muuitos tão aparecendo sem cárie, então é uma doença, talvez, não é só pela questão da fluorterapia, presença de flúor na água de abastecimento, não é só por isso porque já tá nisso há muitos anos, mas pela cultura que hoje tá melhor. Né... Mas... Então acho que dá pra dividir bem. Com relação à valorização dos pacientes da doença, se eles valorizam ou não a doença cárie, são poucos que, realmente, levam isso em consideração, acho que isso é um problema... A grande maioria acha isso, que é normal, que em algum momento da vida vai ter que ter mesmo e ponto final, né, que faz parte da vida, e... E no dia a dia acaba sendo dessa maneira né... Sem pensar no lado técnico é mais pensando nisso...

P: Naqueles casos em que os pacientes, assim, você tem um paciente seu de rotina né, quando ele volta, o mesmo paciente tem sempre muitas lesões de cárie, mesmo nos retornos, como você se sente com relação à esses pacientes?

E: Eu não acho que eu tenha faltado com alguma coisa, porque todos os pacientes eu faço orientação individual até... Né, fazia grupo, não senti que dava muito certo, aí comecei a falar "não, vou ter que fazer individual", faço orientação individual em todas as... É... Todos os retornos. Faz quatro anos que estou nessa mesma unidade, vai fazer, então tem paciente que já tá voltando... Criança, faço retorno até 12 anos de 6 em 6 meses, então tem criança que já vi 5 vezes, 6 vezes... Né... E todos os retornos faço orientação... E... Às vezes, é o que eu falei, às vezes chega a ser meio cultural, falo "ah não, ah tá, então tá... Ah eu acho que eu tô com uma cárie, tá mesmo, e pronto. Acontece isso, é a mesma coisa de ter uma gripe de vez em quando... Que ter uma dor de cabeça... Então ela vai ter. Tem paciente que entende realmente que entende que a falta é dele. Né... Que se eu fiz a minha parte, ele foi orientado, fiz a parte restauradora... Né... Ele já sabe como é que funciona, então paciente que entende fala assim, ah, não não... realmente o fio dental tô deixando pra lá... Então tem paciente que entende, paciente que não. Né... Tem paciente que acha que se ele tá vindo no dentista todo ano não é pra ter nada... Mas... Eu não acho que a falta seja minha, né... Porque eu tento cercar de todos os lados.

P: Sim, mas acaba mexendo com você?

E: No começo eu me frustrava muito. No começo sim. É... Só que aí quando eu percebi que a falta não era minha... Né, então, primeiro, segundo ano de SUS... Quando eu realmente percebi que a falta não era minha, que eu tava fazendo o certo, tava fazendo o meu, o melhor que eu podia, né, do jeito que eu aprendi, né... Então eu comecei a não me frustrar mais. Né, falei assim, então tá, ele não percebe que aquilo é importante, aquilo não é importante pra ele, então eu to dando muito mais importância do que ele... E isso não vai fazer diferença pra essa pessoa, vai fazer diferença pra mim e eu vou me frustrar e pra pessoa não vai surtir efeito nenhum. Então eu parei de me frustrar, abri mão de... De ter dor de cabeça com isso.

P: Entendi...No começo você sentia que era um pouquinho diferente então né?

E: Muito. Muito... No começo assim, eu sofria muito com isso. Sofria mesmo. De falar... Nossa, eu não sei mais o que fazer, não sei mais o que fazer, né... Aí depois eu percebi que a culpa não era minha, que eu tava fazendo o que eu acho melhor, que eu faço o que eu acho melhor, e eles... Pra eles não é tão importante, então... Né...

P: Não é muito fácil, né?

E: Não. Acho que todo mundo que entra no SUS tem esse choque inicial. Minha auxiliar... Teve... Todo mundo tem esse choque da diferença de valorização das coisas.

P: Sei... Você no começo da sua carreira chegou a trabalhar em consultório também, não?

E: Trabalhei...

P: Atualmente você não trabalha mais?

E: Não. Aqui eu faço 40 horas então fica muito puxado pra mim, mas sempre trabalhei, em clínica particular, atendia paciente de convênio, particular... Diferença?

P: É, aí que eu queria chegar, você sentiu alguma coisa de... De diferença?

E: Assim... Eu acho, não sei se é porque... Hoje, já, acabo tendo mais tempo de SUS do que particular, eu acho que os pacientes do SUS, não sei se é porque é de graça, mas hoje, muitos tem a concepção de que tem que ir pro dentista todo ano. Muitos realmente pensam ah não, só vou quando precisa. Eu acho que o particular... Sei lá, 75% vai só quando precisa. Né, não tem esse... São poucos que tem o conceito "eu vou todo ano, mesmo que não tenha nada, pra ver se tá tudo bem, tudo certo, preventivo é que eu vou valorizar", no particular ele só vai quando precisa, consequentemente ele vai demorar pra ir. Ele não vai todo... Ele tenta cuidar. Pra não ir todo ano. Pra não ter que gastar dinheiro todo ano. Acho que acaba sendo esse o pensamento do paciente particular. E o pensamento do paciente do SUS, eu acho que assim, os que pensam na prevenção falam "ah não, vou todo ano mesmo", não paga então ele vem todo ano mesmo, né. E... Mas tem muitos que falam, essa semana tive uma leva de pacientes que veio há 4... Quando eu entrei, ou veio com o Dr C. que foi o que tava antes de mim e veio agora. Né, mesmo adolescente. Já aconteceu... Essa semana mesmo aconteceu bastante isso.

P: Você viu criança e voltou adolescente?

E: Exatamente... E... Mas, tá mudando, tem muito paciente que eu já to percebendo que já tá vindo todo ano certinho, bonitinho, sem nada, sem nenhum problema... Tem alguns poucos que voltam todo ano com problema... Né, que assim, não gostam de escovar os dentes mesmo. Tem uns que já falam mesmo que não gostam, que não sabem "ah não sei, não gosto"... Mas... Eu acho que o valor entre os dois tipos de paciente é esse...

P: Mas atualmente já não dá muito mais canseira em você essa recorrência do mesmo...

E: Não, não dá mais não. Eu não sou... Eu não fico em entrelinhas, eu falo pro paciente "poxa, mas o senhor sabe..." Geralmente pessoal mais velha, mais idosa... Que tem mais dificuldade, às vezes tem até uma dificuldade física mesmo, às vezes realmente não... Fala "ah eu não fiz isso a vida inteira, vou fazer agora...", "ah mas o senhor vai sofrer", né alguma coisa do tipo, "ah não tem problema não", e fica nisso mesmo, mas hoje em dia eu não me estresso mais com isso porque eu sei que não vai surtir efeito.

P: Sei... Mas você acaba voltando no assunto com eles ou não?

E: Volto, volto... Dificilmente eu não falo nada. E como eu acabo tratando a família inteira, então a família inteira sabe o que tá acontecendo né, então vem a esposa falar pra mim, esposa do senhor falando pra mim "Mas eu falo pra ele fazer assim assim assim assado, eu falo pra ele, mas ele não quer fazer" "não esquenta, deixa... Deixa que depois a gente vê o que faz, converso com ele..."

P: Vem toda a família?

E: Vem, quando eu entrei, vinha um de cobaia, vamos dizer assim... É... Vinha um da família de cobaia depois marcava todo o restante da família, hora que terminava o tratamento marcava todo o restante da família, falando do tipo "ah, agora já conheço ela então agora vamo todo mundo", mais ou menos assim...

P: Eu gostaria que você me relatasse um pouco como é sua experiência com, mais especificamente com a parte preventiva no dia a dia, como é que costuma rolar isso?

E: Então, é o que eu falei pra você, antigamente eu fazia orientação de higiene, essas coisas, em grupo, aí eu percebi que assim, metade do grupo não prestava atenção. Então mesmo que, com crianças utilizava escovódromo e tudo mais, aí marcava dias... Como não tinha como marcar todo mundo no mesmo dia, marcava em grupos separados, às vezes... A mãe, quando é criança, a mãe pegava deixava a gente escovando os dentes e virava as costas, não prestava atenção, então, não vi resultado. Não, achei que não deu certo, acho que isso varia muito de população pra população, tem lugar que dá certo, tem lugar que não dá... Comigo não deu certo, então eu comecei a fazer essas orientações individuais. Né... É... Tudo, questão de higiene, ou de orientação bucal de uma maneira geral, o preventivo de cancer... Né... E... Toda vez, todos os retornos eu costumo fazer, relembrar, pergunto se tem dúvida todas as vezes... E aí de fluorterapia eu vou avaliando caso a caso, não faço em todos os pacientes não, avalio caso por caso.

P: Eles recebem isso bem?

E: Então... É... No começo eu perguntava... Se eles sabiam, como que fazia... "Ah eu sei escovar, eu sei passar o fio dental", e não dava certo. Então eu comecei já abordando, termino de fazer anamnese, exame clínico eu já vou direto ensinar a passar o fio dental e escovar. Aí a pessoa percebe e fala "ah não sei, não faço desse jeito", né... Aí deu certo, desse jeito vem dando certo... Eu vou testando, tem coisa assim que aparece diferente e eu vou testando um jeitinho diferente, tem gente que tem que ter uma abordagem diferente, adolescente tem que ter uma abordagem diferente, porque eles não aceitam que ninguém fale o que eles tem que fazer, como eles tem que fazer... Né... Então eu vou aos pouquinhos, falo "Ó, tá vendo esse lugar? Esse lugar você não tá sabendo, não é desse jeitinho, do jeito que você tá fazendo não tá dando certo", eu vou lá e ensino aquele lugarzinho... E assim vai indo...

P: Adolescente não é fácil, né?

E: Não, adolescente acho que é o público mais difícil. Eu acho mais que criança... Nesses casos são os piores, porque não quer escutar, né? Acha que sabe tudo, fala ah "o que eu to fazendo aqui?", né...

P: Como você vê os pacientes lidarem com o próprio problema deles no dia a dia?

E: (silêncio) É... A maioria tem o medo da dor. Né... Eles vem pra cá, mesmo que eles não tenham dor, eles tem medo que ela apareça. Outros tem medo simplesmente que exista cárie na boca deles. Pessoal com um pouco mais de consciência, ou que já tá a mais tempo aqui comigo... Né, então assim, o simples fato de ter cárie pra ele já não é uma coisa boa, a maioria fala assim "ah não, eu tenho cáries mas eu não quero ter dor". A maioria tá mais preocupada com a dor. Né, se tem... "Ah eu tenho cárie, mas vai doer? Vai ter que fazer canal?" ou associa já a dor com canal, logo de cara... Né, então acho que a relação deles com a cárie é essa, a maioria é com a dor. Ter medo de sentir dor, às vezes não tá nem sentindo, cárie de esmalte, mas tem medo de sentir dor.

P: E a parte dele no próprio controle, como você vê isso?

E: Então, é o que eu falei pra você da geração, né... Tem uns que são mais conscientes, tem uns que já entenderam que a prevenção é que cuida da cárie, vamos dizer assim, né... Tem uns que já entenderam, tem uns que não, que acham que, se vem no dentista todo ano não vai é pra ter cárie, né... E é o que eu falei da geração, os mais velhos acabam achando isso. Os mais velhos? Não só os mais velhos, então as pessoas que não tem... Ah, como é que eu posso dizer... Ah, pessoa que é descuidada mesmo, de maneira geral, ela acha que o fato dela vir aqui já não é pra ter cárie, outros já entenderam que não, e assim, o jeito de eu orientar esse tipo de pessoa é que eu falo assim pro paciente "então tá, você vem uma vez por ano aqui comigo, então você vai vir por cerca de... Um mês vai, quatro semanas, uma vez por semana. Quantos outros dias sobram? Então de 30 dias, são 365, então 335 dias no ano, que eu não vou te ver. E isso, pensando que 30 dias eu não te vejo, eu não estou te vendo 30 dias do mês, to te vendo 4, né, então falo assim, o que você faz nos outros 335 eu não tenho controle. Então é isso que eu tento conversar com ele, o que eu faço no meu trabalho é 10%, o trabalho dele é 90. Né, dessas pessoas que realmente acham que é só de vir aqui já tá curada, né... Então realmente, geralmente o que eu acabo abordando, como eu acabo abordando é dessa maneira.

P: (comento algo depositar a responsabilidade no dentista)

E: E não é, e assim, e é... O paciente que faz isso aqui faz isso no médico, faz isso com todo mundo, não é comigo. Ele vai chegar no médico e vai, então tá, o simples fato de eu ir no médico e ele me dar uma receita, e eu tomar aquele remédio, já, pronto, eu já fiz a minha parte, e não é assim. Então o cara vai lá, o cara é diabético, foi lá, pega o remédio dele, toma o remédio mas come pra caramba, não se cuida, não faz atividade física... Ué, mas eu vou no médico a cada 6 meses...

P: E como que você pensa ser a melhor forma, vamos dizer assim, o padrão ouro da odontologia atuar sobre a cárie?

E: Nossa... (silêncio) Eu acho assim, não tem como traçar uma receitinha de bolo, varia muito de paciente pra paciente mesmo, tipo, aquele paciente eu posso ser conservadora, ah então tá, é uma mancha branca que eu consigo controlar, então vamos controlar. Agora, se é um paciente que eu vejo que tem um índice de cárie um pouco maior, índice de placa um pouquinho maior, aí eu vou acabar não sendo conservadora. Né, eu acabo analisando isso, então não tem como traçar assim "não, então vamos ser conservadores", não tem como. Sempre, não dá pra ser conservador sempre. Não tem como falar assim você está com cárie e ponto final, né... Cada paciente é cada paciente, tem paciente que eu consigo levar e tem paciente que eu já errei... Né... De... Eu achava que o paciente ia saber controlar que eu senti que ele aderiu bem à parte preventiva, e tudo mais, chegou aqui e tava com cárie. Né... Então tem paciente que eu já errei. Mas isso... Isso vai, como é paciente que eu sei que vai voltar em retorno e tudo mais, então não chegou ao ponto de ser alguma coisa extrema, né... Mas já errei nesse ponto, de tentar ser conservadora e não deu certo.

P: Comento sobre a virtude de poder acompanhar longitudinalmente e sobre os pacientes que mudam do bairro.

E: Sim, mas os que voltam, voltam sempre. Né, dificilmente o paciente vem no retorno em um ano e depois não vem mais. Os que voltam eles voltam sempre, certinho todo ano. Mesmo que volte (risos) com um monte de coisa, mas volta todo ano. Né, então tem paciente que dá pra traçar um perfil assim... Gigante...

P: E essa relação dentista-paciente, nesse sentido, de aconselhamento e etc, como que é no dia a dia, tem coisa que te marca mais?

E: (silêncio) Eu acho que o principal é, na hora de orientação, o paciente achar que sabe tudo e depois que terminou a orientação você percebe que não sabe nada. Eu acho que isso é o principal, né, porque você pergunta "não, você passa o fio dental? Passo...", né, "todo dia? Todo dia", tudo mais, aí eu pego, mostro o espelho, dou o espelho, mostro como é, tudo o que tem na boca e falo "olha, do jeito que você tá fazendo não tá dando certo" eu não, acabo não... Adolescente eu acabo acusando. Do tipo, falo assim, "não, você não tá passando, ou então tá claro que você não tá passando", né? Agora as outras pessoas eu acabo falando, não tá dando certo, esse jeito que você tá fazendo não tá surtindo efeito, coisa do tipo, né?

P: Você falou que eles às vezes eles falam que sabem, será que eles realmente acham que sabe ou... Eles sabem mesmo?

E: Eles realmente acham que eles sabem... Aí depois eles vem e falam "ah mas eu não faço desse jeito", 99% falam "ah mas eu não faço desse jeito", né, tá vendo, esse meu jeito é melhor, eu não falo que a pessoa tá errada, né... Falo "ah não mas esse jeito acho que é melhor". Aí às vezes eu falo, falo "ó, até a faculdade eu também não sabia, foi na faculdade que eu descobri, porque meus pais não sabiam, eles aprende... me ensinaram do jeito que eles aprenderam... E eles aprenderam com quem, com a minha avó. Minha avó não tem mais um dente na boca há 40 anos, 50 anos! Eu acho que não foi o melhor parâmetro que eles tiveram. Né, então eu acabo passando esse tipo de, aí eles percebem, realmente, putz, eu não sou obriga... Aí eles entendem, eu não sou obrigado a saber, alguém precisa me ensinar. Né, eu não vou nascer sabendo, minha mãe, coitada, também não. E aí... Aí depois eles relaxam um pouco com relação a isso... Eles acham, no começo eles acham ofensa, que eu tô ofendendo. Dizer que eles não sabem, ou que não tá do jeito que tá fazendo não tá dando certo, eles ficam ofendidos. Depois eles percebem que não, realmente eu não tenho obrigação nenhuma de saber. Daqui pra frente eu tenho a obrigação de saber, que agora já me ensinaram.

P: Conforme você fala ele vê que são coisas diferentes do que ele trouxe, né?

E: Isso... E aí, por exemplo, caso de gengivite é bem fácil, de lidar com caso de gengivite porque aí o paciente vem hoje e na semana que vem fala "realmente, do jeito que você me ensinou funcionou, porque depois de dois dias já não tava mais doendo, já não tava mais sangrando..."

P: O desfecho é mais rápido, né?

E: Muito, é porque a reação é muito mais rápida né... Então aí é bem claro assim, quando é gengivite, é bem fácil de entender...

P: Comento sobre ser difícil ver diferença na cárie, por ser crônica

E: É, é muito difícil chegar uma cárie aguda, mas muito, nossa. Acho que se eu vi umas, aguda de verdade mesmo, do jeito que a gente aprendeu na faculdade, sabe? A dentina moole, aquela cooisa... Se eu vi nesses 6 anos de SUS... Umas duas vezes, foi muito. É o que você falou mesmo, por causa da fluorterapia acaba cronificando tudo... Agudona mesmo é difícil, é mais assim, você vê a dentina já, aquela mais, é, a contaminadinha, né... Mas, aguda mesmo, é muito difícil...

P: Queria que você me falasse sobre alguma experiência que marcou bastante você, com relação à essa temática de cárie...

E: Olha, uma coisa que me marcou foi de uma criança... Até outro dia eu comentei... É, acho que foi com os estagiários que eu comentei... Que eu acompanho também as creches, há muito tempo. Então eu tenho até, assim... E todas as crianças, eu avalio as crianças, encaminho as crianças pra mim ou pra dentista que seja do bairro em que mora, ou particular, aí fica a critério, né, de cada um, e aí tem... Realmente aquela criança que não tem o acompanhamento em casa, que não tem o acompanhamento na escola do tipo a mãe nem liga de ir na reunião da escola ou coisa do tipo, acaba não trazendo pra cá. Então eu lembro de uma menininha... Que eu acompanhei ela desde ce...do assim, acho que hoje ela já até tá na escola já, já saiu da creche, ou tá no último ano... Então já tem, sei lá, 3 anos que eu venho acompanhando ela, eu vejo no mínimo uma vez por semestre, no mínimo. Aí eu vi... Um decíduo... Uma cariezinha lá.... Mancha branca... Cavidade... Cavidade maior... Pulpite... Pólipo... Endodontia... Destruição... Extração... A mãe nunca levou. Nunca foi atrás. Foi mese... Anos, eu acompanhando, né... Então isso foi o que mais me chocou, porque era uma criança, a criança sofrendo, ela não podia resolver por si só, não podia simplesmente ir no dentista, alguém tinha que trazer por ela... Eu faço carta, mando colar no caderno, tudo mais, então foram várias vezes, várias cartas dessa... E foi... E teve todo o, todo o... Princípio, meio e fim da história de cárie. E não extraiu o dente, porque também não levou a menina pra extrair o dente... Eu só acompanhei, se eu tivesse tirado foto... Teria visto, falado ó... Interessante e triste, né... E a menina, você vê a menininha... Arrumadinha, é... Não é uma criança que você fala assim "ah ela é maltratada como um todo", não, ela não era maltratada como um todo. Arrumadinha, ia pra escola maquiadinha, roupa bem vestida, né? A mochila, você vê que é uma mochila, uma mochilinha cara, não era coisa barata, ou seja, não é gente que não tem... Que tem pouco recurso. É gente que tem um mínimo de recurso, né.... Só que não valoriza, e o pior, nem para pra pensar que a criança tá sofrendo, porque imagina o quanto que a menina já não sofreu pra chegar no final da história, né? E eu não tinha como intervir dentro da escola, então eu vou lá e faço carta e espero que venha... E não veio, então não sei quanto ao final da história, não vi mais ela... Era um molarzinho, acho que um segundo molar, se não me engano...

P: E não tinha nenhum tipo de resposta, né?

E: Nada, nada... Professora falava assim "olha a mãe não vem, não vem nas reuniões, se vem..." é gente que realmente não participa, que acha que cuidar do filho é deixar ele bem arrumadinho e de banho tomado, ponto. Não é participar do crescimento, da evolução da criança... Aí já... Não posso fazer muita coisa. Até assim, eu tenho umas cartinhas meio "intimação", sabe? Porque eu vou, toda vez que eu vou na creche, hoje mesmo a gente foi, eu levo a... Eu tenho, eu peço pra diretora imprimir pra mim a lista dos alunos. Eu levo a lista anterior, que eu fiz, que tá encaminhado ou não, pra eu saber se eu tô encaminhando novamente a mesma criança. Então eu tenho uma cartinha dizendo que a criança já foi encaminhada outras vezes e que... Medidas cabíveis serão tomadas se não for, se não levar... É assim, não quer dizer que eu vou... Que eu vou denunciar pro conselho tutelar, mas que fique subentendido... Né, pra ver se, pra ver se enxerga que o negócio tá feio, né... Algumas, realmente entendem isso, outras não tão nem aí. A maioria que chega nesse ponto não tá nem aí.

P: É, não é fácil...

E: Não. É triste, triste mesmo.

P: E daqui do consultório, tem algum que você lembra que te marcou?

E: Com história de cárie... Silêncio... Ah, dessa semana que eu falei que teve, criança que virou adolescente, eu fiz restauração de, é, falta de fio dental, tudo interproximal, voltei, criança voltou depois, sei lá, acho que faz uns 3 anos que veio... E tudo infiltradas, as mesmas restaurações... Aí você vê, cheio de placa, cheio de placa mas tem um piercing no nariz... Sabe assim? Cheio de placa mas tem cabelinho todo cheio de gel, então assim... Essa semana mesmo, dois irmãos... E eu atendo a família inteira, a mãe... E pra você ver como não tem... A mãe não veio, a consulta era um menino de... De 14, 15 anos, uma menina de 14 o menino de 15, depois no dia seguinte veio a irmã mais velha, acho que com 18, 19 anos, era pra vir a mãe mas a mãe não veio. Era pra ter vindo a mãe, a mãe não acompanhou os menores... Também não veio pra consulta dela.

P: Como é a questão da orientação pra esses aí, é complicada?

E: Geralmente pra adolesc... Menor de idade, eu espero que tenha um maior de idade pelo menos na primeira consulta, né, que acompanha. Quando tem até 14 anos, geralmente eu peço que esteja em todas as consultas. Ou em consulta que eu acho que vai ser mais puxada, vai ter que abrir uma endo, ou extração, alguma coisa assim, eu peço pra tá junto. De preferência, pai e mãe ou irmãos, tios, alguma coisa assim, porque às vezes chega "ah é meu namorado", a menina tem 15, o cara tem 21, o cara não tem responsabilidade nenhuma, né, então... Aí às vezes eu converso pra vir outra pessoa, né... Mas... Aí, maior de 15, 16, eu peço na primeira consulta pra estar junto. Nesse caso desses dois, como eles já eram retorno, eu achei que ia estar alguém junto. Né, que da outra vez veio, tudo, eles eram menorzinhos, mas... Tudo bem, eu passei, fiz avaliação e como a mãe ia passar em consulta depois né, foi um dia, no dia seguinte a mãe ia passar, que era ontem e não veio, então assim, então tá, o que eu passar pra eles eu passo pra ela depois, mas ela não veio...

P:E o que você acha que pegou mais nesse caso, de não... De você não ter visto a melhora?

E: Eu acho que é... A falta de insistência dos pais, porque nessa idade não vai por si só realmente, no caso deles em específico, né... É falta de insistência dos responsáveis de uma maneira geral e adolescente não, não é pra isso que ele vai ligar. Só vai ligar se tiver que chegar aqui com a cara deste tamanho, porque doer também não vai ser suficiente. Isso eu tô falando do perfil daqui, né, do perfil da região. Doer muitas vezes não vai ser suficiente. Né, ele vai enfiar o que for, uma bala de canhão dentro do dente pra não vim aqui. Aí de ficar com a cara desse tamanho aí ele vem. Aí de repente vai vir. Não vai falar pra mãe, que tá doendo... Porque pra eles, muitas vezes é... É, ah se eu falar pra minha mãe que eu tô com dor ela vai achar que eu sou criancinha e eu não sou criancinha... Né, eu sinto que tem muito disso também, né...

P: Você falou do aumentar porque talvez mexa com a aparência?

E: Também, e porque aí já percebe que chegou no fundo do poço mesmo, que dali é... Caixão. Ai vem. E pra eles não sei se eles acham bonito, o fato de estar sofrendo... Eu já fui adolescente mas eu não lembro de ter passado por isso... Mas... De falar pros outros, falar "ah nossa, tô morrendo de dor de dente", não sei se pra eles é legal isso... Uma auto-afirmação, não sei... Mas a maioria vem aqui obrigado. Ele só vem por si só quando já é de rotina, aí vem. Aí vem por si só. Mas senão, não, porque eu tenho uma meninha também que desde pequenininha ela vem, a rotina, a rotina semestral, aí passou os outros 12 anos e veio rotina anual e ela vem, ela mora aqui na esquina. E ela vem certinho. Era uma bombinha quando só tinha os decíduos, na medida que perdeu os decíduos aí ela cuidou super bem ela fala, e ela mesma falou "ah eu cuido, hoje eu cuido muito bem", hoje mesmo ela fala...

P: Você sente que ela é um pouquinho mais independente da família?

E: Ela é, ela não tem os pais. Ela mora com a avó, porque ela perdeu os pais... E ela faz tudo na casa, cozinha, passa, tudo, desde novinha.

P: Interessante né, justamente o caso mais difícil socialmente...

E: Do tipo "se eu não me virar por mim ninguém vai fazer por mim, então eu tenho que me seguir, e eu não quero ficar com dor", porque ela sofreu, "e eu não quero ter mais dor, então vou seguir o que ela tá falando".

P: Você gostaria de comentar alguma coisa que a gente não tenha perguntado (...)?

E: Acho que não...

(Depois comenta):

Ah o SUS tem muita coisa, nossa, a gente vê de tudo... Eu gosto, é puxado. É bem puxado, né... São os dois extremos, ou as pessoas são muito agradecidas ou elas não são nada agradecidas, parece que você tá aqui por obrigação, você tem obrigação de tratá-los, outras não, elas percebem que realmente você não tá aqui por obrigação, você tá aqui a trabalho... Né... E que a responsabilidade não é minha, é deles... Mas quando tem uma pessoa no dia que já fala obrigado já compensa já... Ó, essas coisas (referindo ao barulho de fora, na recepção), problema social, é muito difícil. É muito difícil, é diferente, meu marido, agora, ele trabalha em Americana, ele é dentista também, agora ele pediu afastamento da prefeitura lá, mas a visão que ele tem, ele trabalha em UBS.. É completamente diferente, UBS de PSF é completamente diferente, você faz pesquisa em UBS também, não? Completamente diferente... Não tem esse envolvimento, tem que ter muito perfil pra trabalhar em PSF, muito. Porque se a pessoa não tem, é... Não quer se envolver, não gosta de se envolver, aqui... Tá certo assim, eu fechei a porta do consultório pra ir embora, eu zero, porque senão, se eu carregar tudo comigo pra casa... Eu não aguento, não guento. Então assim, a pessoa tem que ter o perfil de saber se envolver com todos os problemas da pessoa que tá sentado aqui... Então eu pergunto de tudo, se tá tomando anticoncepcional, se fez papanicolau, direto eu faço diagnóstico que não é meu...

(comento e retomo a diferença com UBS)

É bem diferente o envolvimento, não tem... Você vem aqui pra tratar os dentes, e ponto.

P: O que você prefere, o jeitão de PSF ou de UBS?

E: Não sei, não sei... Eu realmente não sei. Aqui é assim, eu consigo, eu acho que UBS acaba sendo mais paulera, atende mais pacientes porque a área de abrangêcia é maior, é... Só que não tem esse envolvimento, comigo é ao contrário, eu acabo atendendo menos pacientes e tem mais envolvimento, então assim, são... Desgastes diferentes né, eu não sei qual que é pior, se é o desgaste físico ou o desgaste mental... Né, porque as coisas sugam a gente né...

(comento sobre minha experiência com meu trabalho e sobre a repetição mecânica e desgaste mental)

E: Talvez eu não gostasse de UBS por causa disso, aqui eu acabo... Muda muito, né? Me tira da minha zona de conforto, então eu falo assim "ah não adianta eu pegar e exigir daquela criança, quem trouxe aquela criança foi a prima que tem 19 anos que trouxe a criança, por que que a prima de 19 anos trouxe?" porque a mãe trabalha o dia inteiro, porque o pai tá preso, é... O irmão bate nele... Então como é que eu vou pedir pra mãe dessa criança passar fio dental nessa criança? Se ela trabalha assim, 12, 14 horas por dia, a hora que ele chega a criança já tá dormindo, não tem quem cuide, a criança fica o dia inteiro na escola... Então tem assim, é muita coisa pra eu analisar, né... Isso cansa, cansa bastante... Mas, quando você vê resultado... Aí compensa.

P: Comento algo sobre aí ter depois aquele procedimento restaurador

E: Mas a classe II difícil é o de menos nessa hora, sabe... Então assim, o que que eu preciso resolver, tá com dor? Beleza... Então isso, é minha parte, isso eu consigo resolver, essa parte é um problema a menos que aquela criança vai ter, dentro de outros milhares, né...

P: UBS é mais leve nesse sentido, né?

E: É só alí, mas é o mecânico. Você vai lá, vê o odontograma e vai só vai (desgastando)... Isso tira um pouco, o PSF tira um pouco do mecânico, aí eu vou pra creche, aí tem a parte educativa, aí tem essas reuniões de equipe... Aí tem o contato com o restante da equipe, coisa que a UBS não tem... Não tem muito desse contato por perto, né, a da vacina, "ah vamo lá ajudar que tá dando vacina e não tá conseguindo segurar a criança né, esse tipo de coisa, essa interação é que é gostoso, interessante...

P: Comento sobre como seria fazer só a parte mecânica

E: Ah eu não gosto assim... Eu acho que ia ser pouco, sabe assim? Pra mim ia ser pouco, acho...

**Entrevista 4**

P: Você trabalha aqui como dentista e certamente trabalha com a cárie no dia a dia, em diversos aspectos... E essa entrevista é justamente sobre isso, sobre esse trabalho com a cárie. Então eu queria saber como que é pra você, como você se sente, o que você pensa, como que é tua experiência aqui com o tratamento de cárie especificamente?

E: Então... Eu tenho aqui no bairro, eu tô há 4 anos quase aqui né, então eu falo que a minha experiência inicial foi ruim e a minha experiência agora com a cárie é uma outra. Em termos de demanda, tá? Em termos de demanda, isso, vou começar daí porque isso muda um pouco a... A história, né. Ah, no começo, ahh... O número de pessoas que eu atendia com cáries era maior. Né, mas a partir do momento que eu fui resolvendo essa situação, os pacientes foram tratando, ahhh, hoje, olha, como hoje de manhã, eu atendi quase que tudo prevenção, né? Então isso me dá uma satisfação, né, porque eu nunca fui, desde a minha formação de... Graduação, eu tinha, eu tinha esse sentimento assim de não querer trabalhar com uma boca muito doente, né? E eu queria o contrário, eu queria incentivar ou queria mostrar pras pessoas que elas podiam sair dessa situação, né... E isso eu consegui aqui, em tão pouco tempo, né, também porque a clientela responde bem. Eles vêm no retorno, então é isso, né... Então... Aqui, especificamente, eu tenh... Venho tendo um resultado, que me deixaaa... Eh, parece que eu tô confirmando algumas coisas que eu sempre quis fazer ahm, hum, que eu sempre acreditei, assim, que vale a pena você ensinar, a escovar, a passar o fio, vale a pena você cobrar, você, vale a pena você se colocar mais como aliado do paciente como cobrando dele que escove, sabe aquela coisa, que parece que sempre aconteceu assim na odontologia né, então essa coisa do vínculo com o paciente de incentivar lhes, "olha como tá ficando bom, você viu como tava no início, você viu como tá agora", né? Isso me tá dando resultado. Né, aí eu começo a pensar que sempre foi assim, porque eu sempre trabalhei assim, né? Então só que não tinha visto resultados tão rápidos, aqui eu tô vendo resultado muito rápido, né... E... Me deixa... Ass... quando aparece, aparece ainda aqueles pacientes com muita necessidade de tratamento em termos de cárie, me deixa muito... Me deixa aquele sentimento dos meus 20 e tantos anos atrás de profissão, que era só isso que a gente fazia né, e aquilo me angustiava demais, sabe aquela coisa de só remover cárie, fechar cavidade né, não tinha ainda o... O paciente ele não-ã-n... Não entendia e por mais que a gente explicasse, parece que não entendia, culturamente parece que mudou isso. Sabe? Parece que o pacien... Que o paciente já começou a entender que tem a parte dele na história, que ele pode cuidar, que... Então eu acho que por causa desse tempo meu de profissão eu já posso entender isso melhor assim, né?

P: Legal... Você acha que isso veio com o tempo de profissão?

E: Ahhh... De eu sentir? Ah, com certeza, com certeza... Você começa a... A acreditar assim, né, mais que as pessoas podem ter uma condição... Eh, bucal melhor, né... E que assim, aquela coisa ã muito intervencionista não é por ali, e isso é muito forte... Isso eu sinto que é muito forte assim, né. Por exemplo, no início de minha formação eu olhava pra uma lesão crônica, eu já sabia que aquilo não era pra mexer. Mas o paciente muitas vezes queria mexer. Sabe, isso me angustiava. Né, porque eu queria fazer o que eu achava que era certo, mas a boca não era minha! A boca não era minha... O paciente, muitas vezes, "né mas eu queria porque me incomoda, que é preto, que não sei o que", então hoje o paciente já entende melhor isso, né. Um dente posterior, com uma lesão pequena crônica, não quer, você explica e ele já entende que não precisa remover, né... Então... Assim, ã... eu acho que vem... Vem mesmo com os anos e vem com essa mudança cultural do paciente em relação ao tratamento também. Né, você explica que muitas vezes não precisa tratar, não precisa intervir, eles entendem também. É, isso eu sinto bem claro assim.

P: E pelo que você falou você achou que foi uma mudança que vem dos pacientes, da sua parte você acha que alguma coisa que favoreceu isso, ou que alterou isso?

E: Nos meus pacientes eu sinto que sim, todo dia, toda hora... Assim, porque quand... Eu acho que quando você explica, você mostra, você fala, né a, o que, o que, ce, o que você acredita em relação à cárie, o paciente entende. Ã, mais, agora. Mais hoje, mais hoje, um tempo atrás talvez, mas no início da minha profissão era meio complicado isso, né... Assim até da aceitação deles, que eles podiam intervir, né, em termos de tratame... De condição bucal, de saúde, né... Um, ã, o paciente vinha pro dentista como se fosse a única solução pra ele em relação à... Doença, sabe? Era isso que eu sentia. Vinham ali achavam que tava resolvido, e o resto, né, em termos de higiene, n-não tinha, não era... Hoje é muito claro isso, o paciente quer aprender. Paciente quer que você diga pra ele como ele se cui... Que ele tenha um a, né, s-saiba se auto, se... Au... Cuidar, entendeu? (DISFLUENCIA GRANDE)... Então... (Ah - Disfluencia) eu acho que tem de ambos, os os (disfluencia) ambas as partes, tanto minha como deles, porque eu fui sempre mais acreditando nisso. Né... A experiência vai te mostrando assim, nossa é isso mesmo eu tô no caminho certo, não é que eu tô no caminho certo? Sabe, assim? Né, aliás eu tô numa fase muito bacana assim da minha profissão depois desses anos tantos, porque você vai vendo que você acertou, cê fez certo, sabe? Você acreditou e é isso aí mesmo... Né...

P: Você tocou um pouco nesse assunto, mas certamente você já se deparou com aqueles pacientes que... Retornam nas consultas, mas eles sempre reaparecem com cárie. Como é que você se sente frente à essa situação?

E: Eu me sinto um fracasso. Quer dizer, o paci... Eu tenho uma paciente agora, eu tô a segunda vez terminando o tratamento com ela. Essa paciente terminou, ela tava, eu dei um... Eu... Eu sempre marco no cartão o mês do retorno, tá em vermelho assim, pra eles não se perderem muito, não que ele tenha que voltar naquele mês exatamente, mas pra não deixar passar muito tempo, pra, né, dependendo de-da necessidade assim, eu não uso critérios rígidos pra isso né, eu penso "não, essa paciente vai ter que voltar daqui 1 ano", essa paciente eu tinha dado 6 meses. Aí ela não veio em 6 meses... De repente ela aparece... Ela tava com várias lesões, cavidades mesmo. Eu me senti um fracasso... Sabe... Como se eu não tivesse... Conseguido, com essa paciente, né, de ela mant... Se manter bem sozinha, assim, e... Então eu me sinto bem fracassada. Porque eu me empenho demais, eu explico demais, eu-eu ensino fio dental, né, passou, umas, é, ã, algumas consultas passaram eu vejo que, nossa, não tá bom ainda, o quê tá acontecendo ma - Peço (8:08) pra ele me mostrar como tá passando o fio, né... Eu sou muito empenhada nesse... Por causa daquilo que eu falei, porque eu acredito que é por aí... Né, você tem, cê tem, você não consegue trabalhar diferente né, então você acredita que ele vai, ele vai conseguir aprender se... O cuidado, né... E... Então eu me sinto bem fracassada, quando aparece de novo, essa paciente foi um... caso nítido disso assim, aí cê tinha o quê, aí eu tentei investigar com ela... Aí pensei, será que mudou de emprego? Tem aqueles empregos que ficam tomando cafezinho com açúcar o tempo inteiro, mas não, era uma coisa muito... E até agora eu não consegui identificar, a não ser que essa paciente não conseguiu estabelecer um, um hábito de higiene bucal adequado... Sabe... E pode ser, porque eu ve... Eu atendo outros fami... Pessoas da família... E eu vejo que é tudo meio nessa linha, sabe? Eles não conseguem estabelecer um hábito de saúde assim... Parece que não conseguem... Então se por um lado eu sinto meio fracassada, mas é mais por causa do meu empenho, "como que eu não consegui nessa paciente", né, "por quê", né? Mas eu vejo... O lado dela também, né... Mas assim eu me sinto frustrada. No geral eu me sinto bem frustrada, não só quando ele aparece com cárie nova mas quando ele aparece com condição de gengiva... Que não está boa, que não é o teu contexto, mas... Né, assim, uma condição de gengiva que não tá boa também, quer dizer né, nossa... Não... Não consegui né, parece que eu não consegui, sabe... (9:42)

P: Sobre a aplicação de medidas preventivas que a gente costuma usar né, como orientação de higiene, de dieta... O que você pensa sobre elas, como que é tua experiência com relação à essas... Medidas que a gente costuma usar?

E: Bom, um pouco eu já falei né, eu falo disso todo dia, com a mãe da criança, com o paciente adulto, paciente adolescente... Eu vejo que a minha prática nunca passou separado disso, nunca. Porque também(10:22) eu tive uma experiência muito boa preventiva assim no (HIDDEN). Né, no (HIDDEN) a gente trabalhava muito isso assim. Então não sei, eu tenho isso incorporado, eu não trabalho fazendo só... Procedimento. Eu tô fazendo eu já tô falando, eu tô olhando aquela saliva muito pegajosa então ele já pen (disfluencia), "cê anda comendo muito doce? cê t- como cê tá assim, como que é no seu hábito, né, vou conversando e vou fazendo. Porque, é, pra mim é junto isso. Né... Então, e já vou orientando, já vou falando, já... Então é uma coisa que caminha junto com a prática técnica. Não pode, pra mim... Não existe andar separado disso... Né... Principalmente com criança então, meu Deus do céu... É... Criança é mais animador ainda, se trabalhar isso. É mais animador com pediatria porque você explica pra mãe, a criança, ela... Ela cobra da mãe. Pra fazer o que eu tô pedindo. Sabe... E... Então, e depois é mais animador também porque pensa - não, se eu incutir o hábito nessa criança agora, vai ser, né, pra vida toda, você começa a pensar, né. Principalmente as crianças que vem e retornam direitinho, né, eu motivo de novo, faço de novo, então é mais gratificante ainda, você vai ver que ali vai ter um resultado, agora adulto é um pouco mais difícil né. Adolescente também não é difícil. Adolescente também cê pega bem porque você pega pela estética... Né, porque geralmente é uma fase que tá namorando... Então cê pega por aí, né...

P: Aqui no tempo que você tá, você conseguiu acompanhar por um tempo... Assim, longitudinalmente uma criança ou algumas crianças, como que tem sido isso daí?

E: Um resultado muito positivo... Muito... Essa semana eu atendi um menininho de 15 anos, quer dizer ele veio comigo a primeira vez com 11, eu to aqui há quase 4, né... Esse menino... Assim eu peguei ele com os dentin... Os molarzinhos de leite, então... Todos com cárie, agora tão vindo, já tá com os permanentes, porque ele teve a esfoliação dos dentinhos molares mais atrasadinho. Então quer dizer, com 11 anos ele ainda tinha esses dentes todos com cárie extensa, assim. Tá com os dentinhos permanentes todos inteirinhos, ele tem 1 restauração no primeiro molar. Mas assim, e a mãe contente, cê vê a gratific... ah, assim que a mãe fica... Se sente gratificada, né, e e a criança também fica feliz, ah... Mas acho que a mãe mais, porque eles ainda não se dão muito conta do valor disso, né... Mas assim, esse menino aí é um exemplo... É um exemplo.

P: Acompanhar por um tempo e ver né, isso deve ser bacana, mudando a idade, né...

A: Por isso que aquela situação que eu te falei de mudança de... (se referindo ao que tinha falado antes da entrevista, que foi convidada a mudar de unidade) Pra mim foi... Eu quase não dormia a noite. Porque... (choro)

Aqui eu já criei um vínculo com eles, sabe, uma coisa de confiança tão forte, tão bacana...

P: Com eles quem, com os pacientes ou com a equipe?

A: Com os pacientes... Não, com a equipe também... A equipe aqui é ótima. Mas assim, com os pacientes, né, de você já ver resultado. E assim, no dia a dia talvez você nem se dá conta disso... Né... Cê não se dá conta assim de, de de... Quanto importante é o seu papel nesta questão, né, da saúde deles... Porque o dia a dia é essa, essa coisa que a gente conhece, né... Tudo muito rápido, vai, fala e fala e fala, isso que eu te falo assim que a gente não se cansa de falar, e não se cansa de explicar, cê tem a sensação às vezes no dia a dia de que... Você tá só repetindo, né... Cê tá só repetindo... E aí quando cê vê resultado e isso eu te falo assim, depois de 4 anos, você viu o resultado, né... Eu tive, é, eu tive um sentimento assim de... De não querer sair daqui, né... Não, me deixa aqui porque agora eu tô colhendo... É! Deixa aqui que agora eu tô colhendo os frutos... É muito bacana, muito bacana mesmo.

P:Como você vê no geral os pacientes lidarem com seu próprio problema de cárie?

A: Eu acho maravilhoso. Tanto você fazer eles se darem conta do que tá acontecendo, porque assim, a maioria dos pacientes vem com... Vamos pensar na... Na cárie cavita... Na lesão de cárie cavitada já, tá... O paciente, no geral, eles acham que... Ele tava fadado a isso. ‘’todas as pessoas tem cárie ué, por que que eu não teria?”. Quando você começa a fazer ele entender, é... Que ele não tava fadado a isso e que ele pode... É... Ele pode melhorar, ele pode fazer algo, né, ele tem um papel nisso, ahh... cê muda tudo, né... Desculpa, como foi a pergunta?

P: Como você vê os pacientes lidarem com seu próprio problema de cárie?

E: Isso... Então, ah, eles, a maioria dos pacientes lida dessa forma, eles acham que eles vem aqui pra fazer um procedimento, que tá tendo algo que todo mundo tem... Como uma coisa... Que não, não causa estranhamento, ele ´´Aaai, tô com uma...´´, né... Imagina nós, nós, profissionais da odontologia, a gente com uma lesão cariosa... A gente vai achar isso um drama, né? Mas assim, pro lei... Pra eles, na, na na verdade deles, isso é uma coisa que tá acontecendo a toda hora e isso aconteceu com ele também... Né... Então aaaa.... Isso que eu te falei de quando cê ensina o autocuidado, né, que o paciente começa a mudar, ele parece que, tem paciente que parece que dá um clique, sabe, eu acho isso maravilhoso... Eu acho que é isso aí, eu acho que é isso, engraçado que parece que aaaa nossa função é técnica, né... Eu não vejo assim... Eu vou... Aqui a função é técnica pra tirar o perdido, entendeu? Pra, pra... desmanchar o perdido, né... mas a minha função não é técnica... Assim, puramente. Minha função é... Fazer o paciente entender que ele pode mudar situação. Essa situação. Né, e que ele tem um papel nisso. Eu sempre falo pro paciente, ´´olha, eu vou, eu tô fazendo isso aqui por você, isso você, isso você não consegue fazer sozinha ainda, mas você tem que ter essa parte em casa... ´´ e depois, quando eu dou alta, agora você vai saber se cuidar sozinho, entendeu? Vou por aqui, vou marcar o mês de retorno, pra gente acompanhar um pouquinho, ver se você tá indo bem... Mas... Eu tenho cert... né, muitas vezes eu tenho certeza que esse paciente, do jeito que ele tá respondendo, ele não vai precisar de mais nada, né... Talvez ele não precisasse voltar tão... Com aquela frequência tão curta, né... E isso é legal. Isso é muito legal... Cê ver o paciente aprendendo a fazer isso, sabe, tem uns pacientes que... emocionam, sabe... Tem uns pacientes que emocionam... Parece... Nossa, uma coisa muito... Muito gratificante... E eles aprendem e se cuidam, aqueles querem muito, que tão bem motivados a isso, que... Que só tavam naquela condição porque não receberam uma... Ou não tiveram, ou não aproveitaram talvez alguma oportunidade de instrução com relação à isso, mas assim... Mas que quer aprender, esses pacientes vão o resto da vida sem precisar de mim, sem precisar pisar o pé no consultório, sabe... E isso que eu acho que tinha que ser, entendeu... Eu a... Eu acho que não tem que ter aquela coisa assim, certinha ´´Ai de um em um ano... Seis em seis meses´´ (marcando na mesa batendo), né? Isso aí não existe pra mim... Ainda é porque ainda é uma prá... ´´Ahhh e quando que eu volto?´´, sabe, paciente pergunta ´´quando eu volto?´´. Por mim não precisava voltar nunca mais, do jeito que eu to vendo ele motivado, né, mas o paciente pergunta, ele cobra ainda, né... Eu acho que... Que devia partir do paciente. Eu acho que a gente chega, num, assim, determinado momento lá, devia partir do pacien... ´´Ah agora eu vou lá, tô sentindo que a minha gengiva tá mudando e eu não tô conseguindo mudar essa situação, minha gengiva tá ficando avermelhada, eu tenho um sangramento e eu não to conseguindo´´, nem é difícil porque o paciente foi orientado e ele vai reverter, sabe porque tá sangrando, entendeu? Eu expliquei pra ele porque que tá sangrando a gengiva, porque que sangra quando escova... E eu expliquei pra ele o que ele tem que fazer quando acontece isso, que isso é um aviso que a higiene não tá como tinha que ser... Né... Então acho que se partisse dele, eh, eu acho que seria muito melhor... Não sou eu que vou dizer assim, parece, parece uma coisa muito automática né, e a pessoa querer, eh, não sei, de achar que que o paciente tem que ficar indo no dentista, ficar indo no dentista e o dentista que vai dizer o que tem que fazer, eu acho que eu não acho isso... E isso tudo, e isso tudo, assim, eu te falo, eh... Hoje tá mui... Eu formei assim... E... Mas assim, isso foi ficando cada vez mais claro pra mim, sabe, por isso que eu digo que eu não consigo trabalhar diferente, e trabalhar no saúde da família pra mim foi um presente...

P: Comento sobre a formação...

E: Então, agora assim, né, eu comentei contigo que eu formei em enfermagem né, eu acho que isso também, acho que isso também teve um papel assim... Fundamental, na minha forma de... porque já na enfermagem, ahh... Eu, lá em Florianópolis, eu ia pra um lugar que chamava Costeira do Pirajubaé, tinha um... uma unidade de saúde alternativa lá, era da prefeitura mas era alternativa, era um projeto universitário, e a gente ia ver fazer atendimento médico... Eu tinha um livrinho, que, hoje eu não sei se acha em sebo, até já dei esse livro pra uma menina que fez enfermagem há anos, e... O livrinho chamava ´´Onde não há médico´´, né e... E esse livro, fa-falava de técnicas e abordagens, que você... Que não eram tradicionais assim, né... Então eu acho que daí já, eu fui, fui me formando como uma profissional que não... Que não, não... acha que o dentista tem que estar dentro do consultório trabalhando, né... Só... Claro que precisa, eu falei, tem que fazer o que já foi... Claro, não, tem, porque a maioria dos pacientes tem algum tipo de necessidade assim, mas... Eu acho que eu, eu me formei achando que... Não era só isso. Não é o dentista que determina ´´olha, esse dente aqui precisa disso, precisa daquilo´´ não é ele, tudo passa pelo paciente pra mim, entendeu... Não sei se eu consigo explicar bem... Eu não... Não... Coloco o paciente sempre como um agente do tratamento dele, é esse termo que eu queria usar... Claro que assim, o que tá ali cavitado tem que fazer, vou ter que intervir naquele dente, mas... Tem momentos em que o paciente determina o que ele quer, entendeu... Uma restauração... Eu não vou chegar pro paciente, veja só a situação, ele tá com uma resina anterior, no incisivo central superior, que eu tô vendo que não tá muito estética, mas o paciente não teve queixa daquele dente. Sabe, não sou eu que vou dizer ´´eu vou mexer nesse dente porque ta com a coloração alterada´´, sabe, essa coisa muito intervencionista que eu não consigo, que eu acho que não precisa... então... nem, olha... eu, eu me lembro das restaurações de amálgama, porque no inicio da minha carreira é que começou essa história da gente usar resina em dente posterior né, daí, me chegavam aqueles pacientes com aqueles dentes com infiltração porque... Ah, eh... aí parou, começou a virar carne de vaca né, resina em dente posterior, né, ã, eh, aí muitas vezes acho que não obedeciam muito a técnica entã... E o paciente também não ajudava muito porque também não orientavam como é que tinha que cuidar, né, daquilo... Em termos de higiene... E... Tsc... E assim, aí o que eu ia falar é que assim, que boa parte assim de, de de... relatos de colegas meus dentistas era assim, eles propunham a troca de amalgama com resina, né, e... E isso também, nunca foi uma, uma... Um comportamento meu. Mesmo na época que eu tive consultório, não... Ele não tá se incomodando com aquilo, n-não sou eu que vou dizer que precisa trocar. Mesmo em consultório, porque isso é um comportamento típico de consultório, porque ele vai tá ganhando pra fazer aquele procedimento, entendeu... Então é isso, eu acho que sempre... Ã... Eu, eu me formei e eu acho que é isso que me... Que me moveu, assim... Né, e por isso que eu acho que foi muito bacana eu vir pro saúde da família. Porque você trabalha numa, num sentido mais amplo, né, você tá fazendo grupo, você tá fazendo visita domiciliar, você chega lá aquele paciente acamado, família não sabe o que fazer, você vai orientar, ó... Então é muito bacana. Muito bacana. Cê não fica lá no, no consultório direto, né. Você tem mais coisas pra fazer. Entendeu... Cê tem mais coisas de de promoção de saúde pra fazer. Não é ali... Que cê vai fazer, só. É bem bacana...

P: No nosso dia a dia, a gente passa por experiências que às vezes marcam a gente né, sobre esse tema de cárie, você tem alguma experiência que te marcou bastante, algum caso que você lembre de ter sido interessante, assim...

E: Ah, eu acho que o que marca a gente, e eu que eu, ã, sempre lembro assim, é mais assim, mais aquelas situações de início de profissão, porque hoje, você não... Aqui pelo menos e em outras unidades que eu trabalhei, trabalhei aqui em (hidden), coisas de, digamos, de...8 anos pra cá assim, 8 anos pra cá, você não pega mais aquelas condições de boca assim, muito... Né... Mas no início de profissão eu pegava, sabe... Eu... Eu... Eu lembro que eu fui trabalhar, eu trabalhava numa unidade móvel pelo (HIDDEN), numa cidade em Santa Catarina, e... eu acho que a quase totalidade dos paci.. Eu fui trabalhar numa unidade, numa empresa... de.... Plantadores de banana. Era uma empresa que produzia... Ah... Ah esses, como fala, aromatizantes e, coisas a base de banana. Então, depois eu trabalhei em consultório do (HIDDEN), mas esse aí foi meu primeiro emprego como dentista. E... E quase a totalidade dos pacientes vinham com a boca, com os dentes todos perdidos, assim. Né... Dentes pra extração. Tinha que extrair todos os dentes, por problema periodontal, cárie extensa, né... E, e isso acho que foi marcante pra mim, sabe. Era... Eu pensava... Eu não me formei pra fazer isso! Pra, sentir as pessoas tão mutiladas, né... Fisicamente e mutiladas de... Eh... De oportunidades de saúde. Né, eu me sentia muito mal. Isso é muito marcante, tá, então, crianças, não nessa época ali, mas crianças com muitas lesões, ahm... Eu trabalhei, né, eu trabalhei pelo (HIDDEN) nessa unidade móvel depois eu voltei pra (hidden) pra trabalhar, e fui trabalhar pela prefeitura de (hidden), depois eu peguei um (HIDDEN) em (hidden) também e trabalhava pelos dois, que nessa, essa, esse meu início de profissão não teve consultório, como algumas pessoas que já vão, formam, né, fazem alguma especialidade... Não era... Né... E eu me sentia bem assim, porque eu queria fazer algo mais, eu queria fazer acho que alguma coisa... Que fugia dessa coisa muito... Eh... Do que a gente achava que era a odontologia, né... Eu já tava percebendo que eu podia fazer alguma coisa a mais, então era legal nesse sentido... Mas assim, depois, depo... Eh, ah, e aí nessa outra fase de (hidden), na prefeitura eu pegava também uma sit... Umas situações de boca muito assim, complicadas, mas já no (HIDDEN) não, porque, o (HIDDEN) trabalhava com prevenção, por isso que te falei do (HIDDEN). Eu apren... Eu aprendi muito no (HIDDEN), a trabalhar com prevenção, era consultório também, consultório pesado. Atendia muita criança lá. Mas eu tinha a oportunidade de fazer uma coisa mais de prevenção né, então... Bacana foi. Agora o que me marcou mesmo foi isso, acho que mais no início de profissão... E... porque hoje eu não pego mais esses casos assim... Não vejo... Aqui, um ou outro... Mas... Porque agora também parece que não me abala tanto, sabe... Eu acho que eu tô ocupada com outras preocupações em relação à isso, aí então faço porque vejo que é necessário e tal, mas... Mas não é mais assim, não é algo que me choca... Que... Que vai fazer ficar... Agora, por outro lado, eu tenho histórias também, de... De pessoas que, assim, tiveram uma resposta muito boa ao tratamento né, essas também lembro, né... Agora, o que me, me deixa sempre surpresa, são esses pacientes que... Colocam prótese total, ou porque, né, eu encaminhei, talvez daqui que a prótese tava muito desgastada, que isso paciente com 30 anos de prótese total na boca, né, e aparece aqui pra mim, e a... E eu vou, e eu vou, aí eu proponho ´´como é que você tá sentindo, com essa pró´´... Eu nunca sou eu que vou ´´vou mandar fazer uma prótese nova pra você´´, não é assim, entendeu... Cê tá vendo como é que... Então, ´´como é que você tá se sentindo, você tá mastigando bem com essa prótese? Como você tá sentindo ´´, né... Ah então, eu, ah então, não machuca, não nada, tal... Então, se esse pacie... Se eu vejo que tá interferindo em alguma coisa em termos de saúde, esse paciente não tá mastigando direito, paciente relata que não tá bom, que ele não sorri porque a prótese tá toda manchada, aí eu proponho, ´´oh, você sabia que dá pra fazer nova´´, né, aí, o que eu ia falar, é... Que eles vem mostrar a prótese nova, sabe... Sorri... E isso me... Isso também me... Isso é uma coisa mais atual, né, que eu vejo essa felicidade da prótese... e pra eles é uma coisa que foi tão gratificante... E eu fico assim atônita, porque eu, meu Deus, o paciente não se dá conta né, de que ele perdeu, ele tá feliz com a prótese, ele não tá triste porque não tem mais os dentes, sabe... Os dentes dele... Talvez se ele tivesse tido oportunidade, isso que me passa daí... Eu fico com um dó tremendo assim... Então é a realidade dele mas eu penso assim, nooossa...

P: Tem mais algo que você gostaria de comentar?

E: Eu falo assim, que hoje, eu faço um tipo de atendimento, mais... Não sei, eu me sinto muito mais consciente... Sabe, assim do que eu devo fazer, né, de como que eu vou trabalhar com esses pacientes... Eh... Com criança também, eu trabalho muito melhor... E isso tudo falando com relação à cárie. Então acho que... As coisas, de uns anos pra cá mudaram, né...

P: Esse jeito, frente à demanda que existe, você acha isso como?

E: Que mudou a demanda?

P: Não não, você trabalhar dessa forma, é... E a demanda que você tem, de agenda, de horários, isso tem rolado bem, você acha que uma coisa interfere na outra ou não?

E: Ah, a agenda de consultório com outras agendas, com outras atividades?

P: É, com as outras atividades e com esse próprio jeito de trabalhar, você acha que tem...

E: Ah não, eu consigo bem... Eu consigo... Aqui eu consigo bem, você vê que eu to falando que eu não tenho, tem semana que eu não tenho exodontia, né... Então não tenho, por exemplo, no início de profissão eu trabalhava muito com exodontia... Então não dá muito tempo e nem, você não vai fazer procedimento de exodontia, paciente chega com aquela condição extrema, e você vai querer fazer uma abordagem prevencionista, né, conversar, fazer, entendeu, não dá, né, mas os meus, o meu tipo de paciente permite isso, hoje, que a maioria permite. Bem bacana... Cê vê que cê... Aqui no bairro também não é um bairro... ele tem uma parte assim bem carente, mas é um bairro de pessoas que são um pouquinho mais esclarecidas, mas que busca o tratamento pro filho, que vem, que traz, né... Então eu acho que isso facilita também, né... Porque respondem bem...

P: E a resposta aquele seu jeito de você, às vezes deixar na mão dele né, a cada quanto tempo você tem que voltar... Eles recebem bem isso?

E: Não, mas eu, eu... Eu não quis dizer que eu falo, eu gostaria que o paciente tomasse as rédeas de quando ele deve voltar, é nesse sentido, tá. Porque eu não me sinto segura ainda, porque a minha formação não foi assim, né... A minha formação é que dentista determina o tempo que ele deve voltar, e pra eu me sentir mais segura eu faço isso ainda. Entendeu, porque eu marquei, agora você vem... Mês de novembro, né, novembro desse ano. O paciente que precisa de um pouco mais de atenção. Então cê vem em novembro, a gente vê se essa gengiva, se você tá dando conta do recado. Então eu marco, né, porque eu ainda não me sinto segura do paciente dizer, porque eu tenho medo de que o paciente não se dê conta de como tá, ele não consiga reconhecer ou que ele não se sinta motivado o suficiente pra se cuidar...

P: E os outros casos, que você sabe que tão tranquilos, você dá um prazo maior...

E: Ah dou...

P: E eles recebem bem? Não costuma dar problema.

E: Ah sim... Não, não... Recebem bem...

Iamos fechando a entrevista e a dentista lembrou de mais um caso:

E: Lá aconteceu um caso, pra te falar... Uma menina de... Quantos anos tinha aquela menina... Sei lá 13 anos, nem sei... Naquela época a gente não tinha a unidade de referência de especialidades lá. Tinha só de cirurgia, endodontia não tinha, acho que a gente tinha só cirurgia, porque tava começando, quando eu cheguei lá tinha acabando de regionalizar, sabe, tava se organizando. A minha unidade era uma unidade que tinha uma demanda altíssima. A gente até trabalhava naquele esquema modular, que eram duas dentistas, com duas auxiliares na cadeira, depois duas... TSBs, hoje é tsb que na época eram THDs que fechavam as cavidades... E elas tinham cada uma sua auxiliar também, e uma volante no meio do balcão que servia os materiais, tal... E a demanda altíssima, e esse esquema de trabalho foi muito bom pra isso. Porque... A gente rendia bastante, fazia muita coisa... E quando eu saí de lá também, era uma extração por semana, cê vê? Então a gente deu conta de uma demanda que tava lá esperando... E... Aí tinha essa menina de uns 13 anos e não tinha pra onde encaminhar pra fazer o canalzinho do dente, primeiro molar inferior... Direito, permanente... A menina ela era negra, então ela tinha... Alguns negros tem uns dentes bonitos assim, brancos, essa menina tinha um molar assim, e eu abri só a oclusal assim, pra acessar o canal, e o dente tava... inteiro... E a mãe ia ter que extrair aquele dente, e... Eu chamei a mãe e disse pra ela assim: ´´vamo, vamo fazer uma coisa? Cê vai tentando ver uma possibilidade de tratar esse dente, e eu vou fazendo uma troca de curativo, hidróxido de cálcio, fui, fui´´ não levou um ano, ela conseguiu, ela veio falar pra mim que ela tinha conseguido tratar o canal, o tratamento. Não sei como, não lembro mais assim os detalhes, mas que ela tinha conseguido. Aí ela veio me agradecer, e... Então, essas histórias assim, que eu sinto o que eu fiz por aquela menina que é bem bacana, né... Essas histórias, tem outras, sabe, que você faz, porque você tá acreditando, e eu acredito que você vai conseguir fazer esse canalzinho, não vamos extrair esse dente... Né, então é muito legal né, você pensar nossa, eu fico às vezes até pensando ´´ onde será que tá aquele paciente, né, como que tá a vida dele´´, porque você criou até uma, uma coisa meio diferente com o paciente, né... Você apostou que ele conseguisse e ele conseguiu, você sentiu aquilo ali como uma vitória sua também né, é bem legal...

**Entrevista 5**

P: Você trabalha aqui na unidade, como clínico, e... Certamente você trabalha todo dia com a cárie, e essa entrevista é justamente sobre isso, a terapêutica da cárie. Então sobre isso, queria que você me falasse, como que é pra você, como você se sente, o que você pensa, como que é tua experiência aqui na unidade com o tratamento de cárie?

E: Bem extensa viu... Cárie, cárie é que assim, graduação, a gente... Praticamente aprende a trocar restauração. Raramente, pelo menos no caso da faculdade... Uma vez ou outra você vê um dente com cárie mesmo né... Desde que eu vim pra cá pra USF aqui em (hidden), que eu fiquei 4 anos e meio em outra unidade, tô dois 2 anos e meio nessa daqui... Eu aprendi a tirar cárie mesmo aqui, na prefeitura, né... E pela população que a gente atende, tanto naquele bairro como nesse aqui, o pessoal mais carente, né... Cárie assim, é todo dia. Tudo quanto é modalidade, tipo de cavidade, face de dente, dimensão, profundidade, tudo, tudo... Então assim, todos os dias cê treina e aprende um tipo diferente de cárie que cê nunca viu. Cheguei a pegar umas duas ou três classes VI já... Lembra, ponta de cúspide? Então o negócio você não entende direito como que aquilo acontece alí né, o dente tá inteiro bonitinho, na ponta da cúspide do molar tem uma cavidade, mole, então assim, é, tudo quanto é tipo de cárie que cê possa imaginar eu já ví, ou nesse posto ou naquele.

P: Você começou a falar da história de mais trocar restauração... Mas isso você disse que era na faculdade ou... Ou é aqui?

E: É, a minha experiência na faculdade, na graduação, eu não lembro de ter tirado nenhuma cárie, eu lembro de só ter trocado restauração. Aqui eu comecei a ver dente com cavidade aberta assim sabe, que não tem uma restauração que deu infiltração e tem que trocar. O dente hígido, tava hígido, e criou uma cárie agora. Então é a primeira intervenção daquele dente. Isso eu comecei a ver na prefeitura.

P: E com relação à isso aí, essa diferença, que que você sentiu, isso te deu um... Isso mexeu de alguma forma com o jeito que você pensava, ou... Te chamou a atenção?

E: Mexeu um pouco, tá. Porque... Eu lembro que a minha cabeça, antes de vir aqui, a gente tem aquela, aquele receio, todo mundo tem quando sai da graduação assim, de acertar a polpa, ter que fazer endo. Então assim, no começo, questão de 2, 3 meses, primeiros 2, 3 meses da minha vida de consultório público né, cê ia com aquele cuidado, o alta ali na, rotação baixa, vai devagar, fico vendo sempre ali se chegou, não chegou, chegou, não chegou, aquilo tá doendo, demora... Aquela remoção de cárie demorada... Mas de tanto que você vai fazendo a mesma coisa cê já começa a prever o que que vai acontecer, você olha ali e fala "ó essa tá funda, mas provavelmente o dente não vai chegar", então cê já vai, rapidamente você já tira a cárie inteira, então assim, a diferença que eu senti assim... É... Antes de trabalhar com cárie, aquele medo de fazer besteira. Depois que você começou a trabalhar e viu tanta... Cê perde o medo, assim, porque se por acaso vai ser necessário fazer a endo do dente, não foi por causa da minha remoção da cárie né, foi porque a cárie chegou lá, e se ela chegou você tem que tirar onde quer que ela esteja, né, tem que enfiar na cabeça assim, o que eu falo pros alunos da faculdade que vinham aqui fazer o estágio, né... Aonde tiver a cárie você tem que tirar, não importa se vai dar endo, se vai acertar o ligamento periodontal, não adianta, cê tem que tirar tudo, porque se você deixa ela lá, ela vai crescer e vai dar um problema maior, né... A diferença que a gente vê é essa.

P: E certamente você já se deparou com os pacientes aqui que nos retornos eles, reaparecem sempre com cárie, o mesmo paciente que você já atendeu o cara vai lá e reaparece né, nesses casos daí o que você pensa sobre isso, como você se sente frente a esses casos?

E: Eu já vi, eu já identifiquei dois pacientes assim com mentalidades diferentes pra isso. Um paciente que ele acha que a reponsabilidade sobre a saúde dele é sua, é do cirurgião dentista, entendeu, então se você colocou uma restauração a restauração deu algum problema, foi você que não fez direito. Acontece, né... E tem aquele paciente que, nesse caso que eu falei agora é o paciente que não se importa. Então ele joga a responsabilidade pra você. E tem o outro, o outro tipo de paciente, que ele não tem a habilidade, manual, pra conseguir fazer a sua própria higiene. Eu tive uma paciente no outro posto que eu falei olha... Tá ruim, tá com bastante placa, tá com cárie aqui, eu vou precisar dar uma ajeitada no jeito que você vai escovar. Então traz a escova na próxima consulta que eu pego o espelhinho ali, eu mostro como que você faz pra escovar.... Veio trouxe, falei, é, moça, por favor, pega escova, mostra pra mim como você escova. Ela segurou a escova pela cerda, ela escovava com o cabo. Moça tinha 26 anos, então assim... Que que cê faz com um paciente desse, tem que ensinar desde o zero mesmo, como que pega na escova, que parte da escova que cê usa lá dentro do dente pra efetivamente limpar. Então, dos pacientes que eu reparei que são... Cárie sempre recidivantes ali, eu identifiquei esses dois, o que joga a culpa em você e o que não entende o que você tá falando pra ele. Mesmo você falando na linguagem mais simples possível, mostrando, ele não entende. Ou não se importa, não sei, né...

P: Não se importa às vezes né...

E: É... Mas são poucos, tá...

P: Sobre a aplicação de medidas preventivas que a agente costuma usar, orientação de higiene, orientação de dieta, o que você pensa sobre essas medidas, como é sua experiência com esse tipo de coisa aqui?

E: Saber como que eu aplico a prevenção aqui, é isso?

P: É, qual que é sua idéia sobre elas, sobre essa parte de medidas preventivas, orientação de higiene, de dieta, como é sua experiência com elas aqui?

E: Ah...... As orientações de, de, de higiene... Eu sempre faço com pacientes que eu vejo que tão precisando dessas orientações. Eu não faço com todos, tá... Paciente que, aparece ali, é uma cariezinha num lugarzinho, é só uma profilaxia, ou então você percebe que ele até tem um tratamento um pouco mais complexo, mas cê passa a gengiva inteira assim não tem uma gengivite, não tem uma placa, eu não falo nada. Tá... Porque, a, grande parte desses pacientes eu identifico que são tratamentos que foram mal concluídos. Que foi, cárie deixada embaixo de restauração, e por isso que ele tá vindo aqui tendo esses problemas. Agora eu identifiquei que realmente ele tem uma quantidade, quantidade grande de biofillme, gengivite, inflamações, cáries, eu pego o espelho, faço o primeiro tratamento do dia, peço, viu o senhor, próxima consulta, por favor, traz a sua escova, fio dental eu tenho aqui, vou pegar o espelho e ver como você tá escovando... Nós vamos adaptar sua escovação pra ver como você vai melhorar isso daí, né... Então assim, não sei nem se isso seria a forma correta de fazer, mas eu, de uma certa forma eu seleciono os pacientes que eu dou essa orientação, entendeu, isso pensando no tempo clínico que eu tenho aqui, se eu parar todo mundo e falar, ó, vamos fazer, vamos fazer a escovação assim e tal, mostrar, escovação pra todo mundo, eu não atendo a quantidade que eu tenho que atender, né... Então eu acabo deixando as orientações pros pacientes... Entre aspas assim, mais sujos, e pros médio-sujos, quem não tem muita necessidad, muita, quem não apresenta muitas... Consequências de biofilme, eu não falo nada, tá, eu só aviso, "ó, tá tudo legal, não tem mais nada, sua escovação tá bem, se você continuar assim, vamo tratar esse dente, esse dente, esse dente e você continua desse jeito. Eu faço orientação desse jeito, tá.

P: Comento que penso assim, que não tem um só jeito certo de fazer.

E: É a gente vê que, nem todo mundo que precisa de tratamento odontológico é porque não escova direito, né...

Estimulo a falar mais sobre esses casos, retoma mais a frente:

E: Vamo pegar aqui na USF, aqui eu acho que... Uns 30, 40% dos pacientes aqui eu não faço orientação. Que eu vejo assim, essa necessidade...

P: E como você vê os próprios pacientes lidando com o próprio problema da cárie deles?

E: Olha eu tenho, aqui eu acho que eu tenho um percentual assim de uns 60, 80% que eu trato, um ano depois ele volta só pra fazer profilaxia. Mais ou menos essa taxa, tá. Porque assim, os outros, o restante, os 30% ali eu tenho ali uns que eu nunca mais vejo, tem outros que voltam no outro ano e vem a primeira vez com 20 cáries, volta no outro ano com 6. Entendeu, é uma melhora... Nossa... Tratamos tudo e ele volta com 6 ainda, mas tudo bem, a gente trata, aí no outro ano vem, volta com 2... Então demora... Eu vejo assim, esse pessoal com bastante cárie, normalmente não dá certo. Ou abandona, que fica um tratamento extenso, 3, 4 meses tratando, cê vindo ali toda semana... Alguma... A maioria deles em algum momento falha. Um momento não vem, ou então abandona, por... Variados motivos, né... Mas assim, é... Tem algum sucesso. Tem alguns pacientes ali que vem bonitinho, trata 3, 4 meses ali e volta o outro ano, não tem nada. Diria que dos pacientes que tem a boca bem comprometida, acho que mais ou menos metade segue esse padrão, soluciona. A outra metade... Se perde ou volta com o mesmo problema, em menor escala. Agora voltar o problema com a mesma escala, eu nunca tive.

P: Sobre a parte de aconselhamento né, e orientação pro paciente, sobre essa tomada de controle, pra ele controlar o problema dele e etc, como que é isso daí pra você, como que você lida com essa questão de orientação, é tranquilo, não é muito?

E: Tranquilo, tranquilo. Porque eu acho que você tem que ter... Jeito pra falar, com ele, né... Eu, é, simulando uma consulta... Vejo tal o paciente tem bastante dente comprometido falo, ó, sr João, Sr João tô vendo aqui, o senhor tem muitos dentes aqui que nós vamos precisar tratar né, sr tem 15, 20 dentes alí que nós vamos precisar anestesiar, mexer com o motorzinho ali, tirar a cárie, tampar, tal alguns pra tirar, então assim, o que que eu entendo ali do sr, o jeito que o Sr tá escovando, não tá dando certo. Porque o sr tem todos aí pra fazer, que eu mostrei aí no espelhinho, tal. Então, se eu tratar tudo, deixar tudo bonitinho, mandar o senhor embora, daqui 1 ano que o sr voltar aqui pra tratar comigo vai estar tudo igual. Então o que que nós vamos fazer. Traz a escova, traz na próxima consulta, vou pegar o espelho e mostrar pro Sr como o sr vai fazer pra isso aí não voltar, tá... Porque se o sr não mudar o seu jeito de escovar, o problema se repete. Entendeu, a intenção é, a gente tratar, e ensinar o sr a manter tratado, vamos deixar tudo limpinho e a ultima consulta que o sr vier, que a gente fizer a profilaxia, a aplicação de flúor, essa profilaxia ela vai durar até a primeira vez que o sr comer. Sr comeu, já não tá mais limpo. Então eu preciso ensinar o sr a deixar isso dai limpinho pra você manter esse tratamento, é isso que eu faço, entendeu... Costuma dar certo...

P: No dia a dia também as vezes a gente passa por algumas experiências clínicas que as vezes chamam mais a atenção, com relação ao tratamento de cárie, tem alguma experiência que você passou aqui que te marcou mais, que você lembre agora?

E: Teve... Teve dois pacientes que me marcaram. Três na verdade... Uma moça ainda tá em tratamento... Ela... Só sobre cárie, ou sobre tratamento? Uma moça, tá em tratamento, ela tem 19 anos, ela... Ela vai ficar com... 3 dentes embaixo. Só... Conversei com ela, falei "viu, é... Se eu for falar pra você que você tem que tirar todos os dentes eu não tenho, mas os dentes ali que, teoricamente aguentam uma PPR em cima, que tem um suporte legal pra aguentar a mastigação e segurar uma PPR, são 6, tá... São os 2 caninos em cima, os 2 caninos de baixo e um ou outro pré de cada lado, embaixo. Os outros... É aquela cárie que já tá no nível cervical assim e, na dentina ela já socavou por baixo assim, então é dente que não... Tem como, assim, como recuperar. Então assim... Eu deixo na sua mão, tá... Se eu for pegar o livro, o que tá escrito lá, você tira esse, esse esse aqui, deixa os 6, e daí esses 6 você vai no particular, pagar pra fazer uma PPR, tá... Qual que é o outro tratamento que é possível... A gente tirar TUDO e eu encaminho você pra fazer um par de dentadura, que daí você não tem custo. Agora, cê... É, conversa em casa... Que 19 anos, apesar de ser maior de idade, vive com os pais, né... Conversa em casa, veja lá, que no meu entendimento, você colocar uma, uma prótese total em cima, e colocar uma PPR suportada com 2 dentes, não vejo muita diferença, né... Esse é um caso, tá em andamento ainda, não terminou.

P: E nesse caso aí, especificando um pouco mais nele, você identificou algo de muito diferente, que tenha levado ela pra aquele quadro, ou... Parece um paciente como qualquer outro?

E: Eu tendo a não perguntar. Porque... Algumas vezes que a gente pergunta... Ele acaba remexendo em coisa que o paciente não gosta. Já teve pacientes lá, eu aprendi há muito tempo assim que os casos que me fez aprender isso eu nem lembro mais porque, mas de vez em quando minha auxiliar pergunta, sabe... Pergunta, nossa o que aconteceu moço, que você tá com os dentes da frente tudo cariado, tudo quebrando, não sei o que, pá, aí o paciente vira assim pra ela e fala "Ó eu passei 10 anos na cadeia" (risos), desmonta você... Entendeu... Então eu já tendo a nem perguntar mais. Eu, ao invés de focar no motivo, por que que tá daquele jeito lá, eu pego e falo tá assim, vamo trabalhar pra ficar legal, eu não penso muito pra trás, entendeu... Mesmo porque, no meu ver, não vai ter mais nada o que fazer pra trás... Né...

P: Você acha que no começo esse tipo de situação mexia diferente com você ou você sempre pensou assim?

E: No começo eu ficava mais impressionado. Mas... Aquela história, de tanto ver... A gente acaba ficando duro, né... Raramente tem alguma coisa que me surpreende agora. A gente fica sabendo de um caso menininha de 6 anos que cuida da irmã recém-nascida, parou de ir na escola e cuida da irmã recém-nascida, entendeu, ela tem 6 anos... Porque o companheiro largou da mãe, e a mãe não quer tratar da criaça... Largou a escola e tá cuidando do nenê recém-nascido sozinho. Então você acaba ficando duro, sabe... Nada mais surpreende, nada mesmo.

P: Você ia continuar dos outros casos...

E: Teve um dum rapaz... Não tinha nem tanto problema assim, o problema dele era periodontite, ele tinha acho que uns 16 anos, 16, 17 anos, isso aí foi no começo do ano agora aí em Janeiro. Era uma perio P1, mas aquele sangramento né, todos os dentes ali, falei "olha, o problema seu não é grave, a gente, só que vai ter que tratar né, senão fica sangrando, fica esse hálito que você tá reclamando pra mim, né, o tratamento é assim, assim assim e vamo". Foi um tratamento traquilo, foram duas ou três consultas ali, uma raspagem, profilaxia, fluor, acabou. Terminou a última consulta, mulher é mais fácil, mais comum fazer isso né, agora o homem, adolescente ainda, tá naquela fase, chega assim "doutor, muito obrigado", começar a chorar e te abraçar... Né, aquele marcou, "muito obrigado, tava muito preocupado, agradeço de coração, abraçou eu", falei nossa... Eu nunca ia fazer isso quando era adolescente, nem hoje não faria, risos... Isso aí, falei gente, impressionou, entendeu? Gratificante...

P: Você lembra do terceiro caso?

E: Terceiro caso acho que eu terminei ano passado ou retrasado, paciente daqui também, de... Restaurei todos os dentes dele, eu mexi em todos, entre restauração, endo, raspagem e exo... Todos... Teve um que não foi tratado ali, então foi um tratamento bem comprido... E ele tem problema com... Alcoolismo, drogas também, sabe... A consulta ele vinha em todas, bonitinho. Terminou, tratei, tudo, um tratamento complicado, sabe, que cê... Porque normalmente quando o paciente tem muita coisa pra fazer, no começo do tratamento parece que cê não tá fazendo nada, você faz um, faz outro, faz outro, paciente chega pra consulta, dá um sorriso, tá igual... Não tá, você vê que o tratamento não tá indo pra frente, né... Mas daí conseguimos, tratei uns 4 meses, 5 meses de tratamento ali... Daí terminou a última consulta também, ele falou "nossa doutor, muito obrigado viu", ele tem acho que uns 25, 30 anos... Dr muito obrigado, vocês são muito gente boa, vou vim aqui visitar vocês, no futuro ali... Foi um caso que eu duvidava muito que ele ia continuar... Porque ele tem aquele, comportamento... Como eu digo pra você, aquele comportamento que faz você entender que é uma pessoa desleixada, que anda balançando os braços, olhando pra cima, aqueles bonés virados de lado assim... Mas veio em todas, não deu... Não deu trabalho nenhuma vez, deixava anestesiar tranquilo, tratava tranquilo, ia embora, apertava a mão... Chegava alcoolizado algumas vezes, mas era aquele bêbado que não dá trabalho sabe, ficava quieto, então... Foi sossegado, e foi um tratamento assim que terminou assim, você via a diferença nele...

P: Nesses casos muito, né, você falou que precisou mexer em todos os dentes, a parte que a gente tava conversando antes, de orientação de higiene tal, como que foi nesse caso? Você chegou a trabalhar isso com ele?

E: Trabalhei, trabalhei, trabalhei... Na primeira consulta eu mostrei tudo, como é que ele fazia, a escovação, a higiene, o fio dental... Segunda consulta ele ainda tava mais ou menos, não tava muito... Bem escovadinho assim não, insisti mais uma vez... Terceira consulta pra frente, acabou, tudo limpinho. Tudo cariado, tudo com... Endo pra fazer, exo pra fazer, mas... Biofilme, gengivite, não tinha mais. Daí foi indo até o final.

P: Pergunto se tem alguma coisa mais a complementar e etc

E: Acho estranho só como o pessoal aí por fora costuma tratar selamento biológico como cárie... Eu não sei se é falta de instrução ou se é... É uma forma de ganhar dinheiro entendeu, na ignorância do paciente. Nossa, muito, muito mesmo... Tá tudo riscadinho, tudo quanto é fissura, fóssula, tá tudo preto. Mas, nada de cárie, nada... Ou quando vai, vai colocar aparelho, depois vem aqui no consultório e fala "a dentista falou que tem 8 cáries, nunca tive cárie e falou que tem 8... Cê vê lá tem 8 dentes com selamento biológico... Paciente fica assim né, quem que tá falando a verdade, aí eu pego o espelho. Pego o espelho, ponho pra ele, falo tá vendo aqui ó, vou passar a sonda aqui pra você ver, tá duro... Não sai isso daqui. Tá... Aí eu explico tudo, tá, é uma bactéria que tá ali, que ela protege a região contra a cárie, não sei o quê... Ai falo ó, deixo com você, você quer continuar com seu dentista do aparelho lá, a gente tira tudo o selamento biológico e coloca restauração. Entendeu? Mas assim, se fosse comigo... Eu não faria. Né, porque você vai trocar um dente hígido por um dente restaurado. Eu não faria...

P: O que você sente de resposta deles?

E: Eles costumam acreditar em mim (risos), eu já tirei paciente de vários ortos já com essa conversa... É, não é conversa, é verdade... A maioria eu falo assim viu, a maioria desse pessoal tem 14, 16 anos aí vem com o pai, com a mãe, é a idade que o pessoal põe aparelho... Tá vendo esse dente aqui, ó... Primeiro molar inferior, ele nasce com uns 6, 7 anos de idade. Tá... Então faz tempo, pergunto pra criança, faz tempo que você tem esses risquinho aqui em cima do dente? "Ah faz tempo", então... Faz 8, 10 anos que cê tem esse risquinho aí... Pra que nós vamos mexer? Né... Costuma ser um argumento bom. Eu tenho 30 anos e tenho isso daí desde adolescente, né, não tenho nada... É, acho que é aqueles dentistas véio né que olha assim "preto é cárie", e o paciente também, preto é cárie. Acho que é isso...

E: Uma das... Uma das opções que eu dou pro paciente, e assim, isso eu tenho comigo, já desenvolvi isso depois de um tempo de formado, né, não saí com esse pensamento que eu tenho hoje, que é assim... Eu acho que quem manda mesmo... Quem manda mesmo no dente do paciente, na minha opinião, é ele. O dente é dele. Eu falo, ó, paciente chega com dor assim, com aquela cárie, falo assim ó, seguinte, tem duas coisas que vão resolver o seu, sua dor. Por exemplo. No caso do seu dente a gente tem dois caminhos. Primeiro caminho, a gente faz o tratamento de canal, faz tratamento de canal, eu faço a primeira fase, encaminho você, faz com especialista lá no centro de especialidades, daí ele manda você aqui de volta pra mim, a gente faz uma restauração... É pra melhorar. Esse é um caminho, o outro caminho, tira o dente. Não é um dente perdido, é um dente que tem recuperação, mas assim... Você tem essas duas opções. Endo, canal, ou extração, tá. De um lado é um tratamento ali que você vai ficar 1, 2 meses ali tratando, até voltar com o dente tudo bem, o outro tratamento 20 minutos tá tratado mas só que você fica sem o dente. O que você prefere? O que ele escolher eu faço. Isso eu faço frequentemente... Eu saí da faculdade, eu saia com aquele, tipo orçamento, né, que o pessoal fala, tem que fazer isso, tem que fazer isso, isso isso isso isso, o tratamento é fechado, esse dente não é perdido, é endo, nós vamos fazer o canal e vamos restaurar depois. Quando eu trabalhava no particular muitas vezes eu falava isso o paciente queria tirar, ele ia no outro alí na esquina que tirava e eu perdia o paciente, né... Eu tenho isso comigo ainda, quem manda é ele, eu dou as opções e ele escolhe. Eu saí da faculdade pensando como aluno da graduação (risos) né, é isso que eu tenho que fazer. As vezes não, as vezes paciente quer outra coisa.

P: No começo quando ele queria outra coisa...

E: Ah não, é assim que tem que fazer porque assim que é certo. Não é, ele ia em outro.

**Entrevista 6**

P: Fale como é sua experiência no manejo clínico do dia-a-dia da cárie, como você se sente, o que você pensa, como é sua experiência aqui sobre isso?

E: Ah, é a, é ainda a doença principal, né, como todos nós sabemos, eu acho que em relação aos adultos é aquela questão da polaridade, tem muitos pacientes que não tem cáries ou tem um número bastante reduzido, em contrapartida tem alguns pacientes que muitas cáries. Eu percebo que muitos pacientes, principalmente adultos, vem de outros estados, e nos estados de origem eles tem bastante dificuldade em acesso ao tratamento odontológico, então eles chegam aqui com várias cavidades, com cáries muito grandes, muita extrações indicadas, mas por conta das dificuldade no acesso ao tratamento, mas assim, trabalhando em periferia, com uma população com um poder aquisitivo mais baixo, é algo comum, ter muitas cáries, isso ocorre bastante ainda.

P:Sei, e o manejo no dia a dia, como é que é, o que você sente com relação à isso?

E: É, eu costumo assim, é, quando eu percebo que o paciente tem muita necessidade, eu costumo fazer assim, primeiramente remoção de cárie é, abertura, acesso pra polpa de alguns dentes que tem, que são mais passíveis de dor, as extrações que são indicadas, e depois começo a fazer, é... Como chama, me fugiu o nome... Ah, eu removo a cárie, coloco curativo, e aí eu tento conversar com os pacientes, orientar... Mas assim, eu sou da opinião que, né, eu acho que eu mudei um pouco meu conceito. Quando eu fiz faculdade, a alguns anos atrás (risos), nós eramos orientados, né, o que nós aprendemos, foi "oriente o paciente porque o paciente tem que fazer isso, tem que fazer aquilo" né, é isso que o paciente tem que fazer, então é muuito direcionado, muito unilateral, bastante verticalizado, só que eu acho que com o tempo eu percebi, n-no dia a dia, a gente percebe que muitas vezes n-não funciona né, então eu vejo assim, eu tento assim motivar o paciente né, eu falo "olha, a situação é essa, a situação dos seus dentes não está boa, essas serão as consequências e mais pra frente você vai sofrer as consequências da situação que hoje a gente encontra", porém eu sempre tento passar pro paciente que é ele quem deve decidir né, então eu tento motivar, eu explico, eu, né, eu... Na verdade eu sempre falo assim olha, eu não tô mandando você fazer tal coisa, eu estou aconselhando, acho que cabe ao paciente escolher. Assim eu vejo muito assim, até pra nós mesmos, eu vou ao médico, ele fala "ai, coma só salada, frutinha, nada de gordura, nada de açúcar", eu, eu sei disso né, mas cabe a mim, né, a responsabilidade de aceitar ou não mas eu sei das responsab... eu me responsabilizo por aquilo que eu faço, por aquilo que eu decido, mas assim, a questão de optar por isso é minha, e eu acho que o paciente também tem esse direito né, é diferente eu acho, que é um pouquinho diferente quando a questão é com as crianças né, aí eu tento né, já explico várias vezes né, eu converso com as crianças, mas eu tento, assim, sempre deixar bastante claro que a mãe precisa, né, também ajudar nesse enfoque, as vezes a mãe fala "ah, puxa, mas bala, chiclete, ele ganha do vizinho, o vô traz", então isso envolve a família mesmo, e às vezes não se sabe se é sogro, se é sogra, se é vó, então isso é um pouco complicado né, mas aí eu tento conversar né, já cheguei a ponto assim né, manda uma cartinha pro pai... Porque isso é uma questão muito comum, quando os pais são separados "ah em casa eu cuido, mas quando vai com o pai é diferente", então já fiz até cartinha pedindo né, pra manter uma escovação adequada, controle de alimentação, então assim, eu tento motivar os pacientes, eu acho que isso é importante, acho que abrir e fechar cavidade acho que é muito fácil mas não resolve o problema, mas eu deixo sempre bem claro pro paciente que é ele que deve decidir por ele mesmo.

P: Você falou que tem uma pequena diferença entre adulto e criança, como que você vê, mais no caso dos adultos, eles lidarem com o próprio problema da cárie?

E: Em relação aos comportamentos...? É, na verdade assim, às vezes a pessoa, o paciente vem e ele tá bem assim, bem consciente daquilo que... Ah, eu estou com muitas cáries mas por conta... É, devido ao trabalho, eu não consigo escovar várias vezes, ah não consigo muitas vezes passar o fio dental, acabo esquecendo, é uma coisa muito difíc... Talvez a coisa mais difícil pra nossa profissão não é a motivação para escovar os dentes, mas sim para o uso de fio dental, eu acho que é mais difícil o paciente utilizar o fio dental do que a escovação propriamente dita... (interrupção de colega de outra unidade que veio pegar assinatura)...

P: Você tava naquela parte do, dos próprios pacientes lidarem com seu problema de cárie, você parou na história do fio né...

E: É então, assim, às vezes os pacientes tem plena consciência, às vezes eles vem e falam "olha, faz tempo que eu não vou no dentista, mas eu sei que a situação, eu tenho várias cáries, mas eu sei que o meu comportamento né, os meus hábitos culminaram pra isso".

P: Eles mesmos falam?

E: Gente, a maioria! Ai eu tenho alguns pacientes que tem cáries mas eles falam "puxa, mas eu escovo os dentes, eu passo o fio dental" só que às vezes são comportamentos que eles não... Não conseguem entender, um exemplo claro, semana passada veio um paciente com várias cáries em cervical, manchas brancas, aí eu comentei dessas manchas com ele né, expliquei e aí ele comentou comigo que ele não gostava de doces, não comia doces, que ele não tinha esse costume, aí eu falei "olha, mas alguma coisa está acontecendo nesse momento... Você belisca, chega, várias vezes tá comendo alguma coisa e tal", (e ele) "ah uma coisa que eu faço é tomar café", "várias vezes por dia?", "várias vezes por dia", "mas com açúcar?", "com açúcar... Aaahh é né...", então assim, as vezes é difícil, é por falta de... Assim, ele não, não... Não caiu a ficha de que aquele comportamento poderia alterar né, a situação bucal dele, mas normalmente os pacientes já vem conscientes né, "eu já tenho várias cáries", né eu não tomei cuidado por muito tempo, então a maioria tem mais ou menos esse posicionamento, um pouco diferente às vezes das crianças que as vezes tem aquela questão cultural, que as vezes eu explico, mas quando a pessoa né, é difícil lidar com essa questão cultural né "ah é o antibiótico, é porque ficou doente", então essa questão fica mais, mais difícil para as crianças, né, então o controle de cárie, às vezes dependendo da criança é um pouquinho mais difícil mas normalmente os pacientes tem consciência do que, do que tá acontecendo e quais os comportamentos levaram àquela situação bucal.

P: Tem consciência né...

E: Ah normalmente, aliás tem uns pacientes que chegam e já falam "não vai se assustar" (risos). Ou então "olha, faz dez anos que não vou ao dentista", ou porque não pôde, ou porque é uma questão de trabalho "ah meu chefe não deixa", ou então, muitos falam que é por medo, porque não gostam de ir ao dentista, então aqui assim, eu converso muito com o paciente acho que, por eu gostar de criança eu acabo conversando com os pacientes então a maioria dos adultos que tem medo, eles, de repente, não consigam superar o medo, mas assim, mais importante é que ele consiga tratar, então assim a maioria dos pacientes eles acabam conseguindo tratar.

P: Legal... E como que é pra você lidar com os casos bem avançados de doença, que que você, como que rola isso aqui.

E: Ah então... Não tem muito, (disfluência) graças a Deus nós não temos muito casos muito assim, pacientes com muitas cáries, mas nesses poucos casos que ocorre, eu tento fazer assim, o problema dessas cáries avançadas é a perda de estrutura né, a perda de estrutura acaba afetando depois a resistência do dente que futuramente acaba havendo fraturas e tal, mas eu deixo bem ciente, eu esclareço muito, eu sou bem sincera com os pacientes nessa questão, eu deixo claro a real situação dele, do que deve ser feito e quais as consequências lá pra frente, porque eu acho que desde que ele saiba o que tá acontecendo eu acho que ele não se assusta se, ele, de repente... Caso ocorra, então isso é bem comum "ah aconteceu tal coisa que você já tinha me falado", então é bem comum, então assim, eu faço a abertura, dos dentes que potencialmente ou que já tem sensibilidade dolorosa, ou que potencialmente podem vir a doer, aí eu faço a abertura, as vezes já faço o acesso do canal, aí o que é indicado pra extração a gente faz ou é encaminhado pra especialidade, e aí assim, eu tento fazer o que é possível, mas a gente tem algumas limitações né, por exemplo, ah é... Endodontia de molar na prefeitura é até 26 anos, aí então, assim, me restringe um pouco, aí por exemplo, num caso... Logicamente eu entendo a situação da prefeitura né, toda prefeitura tá com dificuldade né, mas por exemplo, num caso de prótese total é possível fazer na prefeitura, mas um caso de PPR ou prótese unitária não, então isso restringe bastante o tratamento, mas tudo o que é possível ser feito aqui eu acabo fazendo, mas eu sei que diante da situação do paciente não são todas as necessidades dele, mas a gente faz o que é possível ser feito na prefeitura.

P: E essa limitação, como intefere no dia a dia...

E: Ah interfere pois eu sei que o paciente tem uma necessidade que a prefeitura não pode resolver, então assim, eu tento passar pros pacientes quais são as alternativas, então por exemplo, no caso de endodontia de molar, eu encaminho pra (HIDDEN), peço pra que ele entre em contato na APCD para ver se naquele momento tem vagas disponíveis, no caso de PPR também, de prótese unitária, nos casos de, reconstruções... Assim, há muito tempo atrás quando eu fazia clínica geral eu não fazia muitas reconstruções porque eu sabia que eram dentes que fatalmente iriam, né, dariam problema, ou fratura, ou fratura do dente, então é difícil, então eram coisas que eu não fazia eu já falava "olha, tá indicado pra prótese unitária", já deixava bem claro pro paciente, só que aqui, eu assim, eu me compadeço pela situação dos pacientes porque eles falam "olha eu realmente não tenho condições de fazer", então eu tento fazer reconstruções -eu sei que não é o ideal - que na adaptação das restaurações, principalmente em classe II, são realmente restaurações extensas eu acabo tentando fazer reconstrução mas aí eu deixo bem claro pro paciente a situação que, se quebrar, quando quebrar, provavelmente esse dente já vai pra extração, muitas vezes isso já aconteceu, mas aí paciente diz, mas eu tento fazer o que é possível, mas muitas vezes, se só tem a raíz mesmo, não tem como eu fazer nada, mas eu tento fazer o que é possível, mas é limitante, com certeza, ou as vezes algum paciente que tem DTM, ou então o paciente não tem cáries mas tem necessidade de acompanhamento ortodôntico que é muito comum pras crianças, tem sido assim uma constante, faço avaliação nas crianças né, aí, hoje, graças a Deus a gente percebe que poucas crianças tem muitas cáries, o restante não, e aí eles vem todos felizinhos "ah eu vou precisar usar aparelho?" "Ah você vai precisar", eles ficam todos felizes, mas TOODOS precisam, a maioria precisa, então isso é uma limitação grande né, porque é uma intervenção precoce, poderia evitar, tudo, você vê alguns pacientes que, quando adultos irão pra ortognática, isso é uma realidade, então... Bastante limitante nessa situação, mas eu entendo né, a prefeitura realmente não consegue comportar todas as necessidades dos pacientes, mas assim, a gente trabalha com o que a gente tem e a gente tenta orientar os pacientes, alguns vão pra (HIDDEN), alguns acabam fazendo tratamento na... Mas assim, eu percebo que muitos pacientes vão atrás de tratamento aí eles retornam, muitos já fizeram implante na APCD, na (HIDDEN), então assim, percebo que quando a pessoa tem o interesse, ela vai atrás, ela junta dinheiro, tem muitos pacientes que fazem isso... E aí eles retornam pra fazer as manutenções né, é gostoso quando a gente vê, é gratificante... Aí tem alguns pacientes que retornam, eu percebo que eles tão com aparelho montado, a maioria acaba colocando em profissional particular mesmo, aí eu sempre oriento que se precisar fazer restauração, as limpezas periódicas ele pode retornar aqui, então assim, o que você puder fazer aqui na prefeitura está disponível, faça tudo aqui na prefeitura e faça o que não é possível ser feito aqui, nos dentistas particulares.

P: É, você falou da limpeza, como costuma rolar essa parte de, como que costuma rolar o trabalho preventivo no dia a dia?

E: Ah é assim, depende muito... Nos adultos, quando a pessoa tem muitas cáries, principalmente cáries interproximais, eu faço um intervalo de tempo menor, então a cada 6 meses, ou então aqueles pacientes que formam muito cálculo, então eles acabam passando antes, mas é mais comum isso, os controles mais frequentes em crianças, então tem crianças que passam comigo há bastaaante tempo...

P: Bastante tempo...

E: AH, bastante (risos), eu tô há vários anos aqui, há vários anos eu atendo alguns, só que é a única forma de estabilizar o quadro, não tem outra alternativa, então, tem alguns pacientes que eu atendia assim com quatro, cinco anos, que hoje tem oito, nove, então eu sempre faço os controles, as manutenções, eu explico e lógico, você sempre tem que motivar porque um dia ou outro, né, tem mais placa bacteriana, então eu explico, eu mostro, eu converso com a mãe, eu sei que assim essa motivação ela precisa ser periódica, porque, lógico todo mundo tem problemas, nós temos problemas então assim, é importante a gente sempre frisar a importância, e eu percebo assim, muitos casos, tem aqueles pacientes que acabam desistindo, tem uma dificuldade ou outra, mas esses que persistem, que eu observo, eu percebo bastante melhora, que estabiliza o quadro, aí eles perdem naturalmente, esfoliam os dentes decíduos, vem os dentes permanentes sem cáries então eu sempre friso a importância: "olha, ele teve cárie em dentinho de leite, não é por isso que ele terá em dentes permanentes mas aumenta a chance", então assim, eu percebo que tem alguns pacientes aqui, agora eu não tenho percebido tanto, mas teve um período de tempo em que eu tinha vários casos de hipoplasia de esmalte, então, nesses casos o controle é mais frequente mesmo, porque eles tem maior recidiva de cárie, maior perda de estrutura por desgaste, então nesses casos realmente o acompanhamento é mais próximo mesmo.

P: Mais próximo...

E: Sim, tem alguns pacientes que são bem próximos (risos), então assim "ah, daqui a dois meses deixa eu dar uma olhadinha, ah daqui a três meses", aí conforme eu pecebo uma melhora no quadro, porque assim, não sei se é pelo fato de eu trabalhar muito com crianças, você percebe quando o paciente tá um pouco melhor, tá um pouco pior, você percebe diferenças em, ah, perde restaurações ou tem manchas novas, então quando eu percebo que tá assim eu já diminuo, né, o intervalo de tempo, então assim, eu tento trabalhar, e assim, desde que a mãe colabore em relação à frequência mesmo, assim, eu percebo que... Só que infelizmente, eu vou ser bem sincera, eu gostaria de ter mais pacientes nessas condições, de ter controle mais próximo, porque muitas vezes eles acabam desistindo no meio do caminho...

P:O que acontece com esses...

E: Olha... É pela questão, acho, questão de tempo mesmo, porque a maioria das pessoas agora trabalha, porque a criança já estuda em outra escola que não é no bairro, isso é muito comum também, mas assim, eu gostaria que esses controles fossem mais frequentes com a maioria das crianças, entendeu, porque eu acho que faz muita diferença, eu tenho pacientes que, por exemplo, tem hipoplasia de esmalte, que hoje eu faço o controle, que se, eu falo pra mãe e é verdade, se ela não tivesse a persistência né, a orientação de sempre retornar, ele provavelmente teria perdido aquele dente né, então, porque é um caso grave né, de hipoplasia, então acho que assim, desde que eles retornem mesmo eu percebo essa diferença sim, e aí sempre assim, pras crianças realmente os retornos são mais frequentes, pros adultos dependendo da necessidade.

P: Como você pensa que a odontologia poderia atuar sobre a cárie, poderia assim num sentido mais... Talvez... Seria o ideal, vamos dizer assim...

E: Olha, eu acho... Ah eu acho que é complicado, porque isso é a longo prazo e... E é um pouco contraditório porque... No mercado capitalista, o enfoque é um pouquinho diferente né, eu percebo pelos pacientes que me perguntam "ah, qual a melhor pasta né" e eles sabem os nomes das pastas, de dentes, "ah Total 12, Whitness, ah porque aquela escrova cross não sei o que da Oral-B, ah porque aquela pasta que é enxaguante..." eu falo assim "gente, o importante é remoção mecânica de biofilme" né, então assim, é importante que você utilize, né, tudo bem, mas é importante que você faça o uso de fio dental, que você faça escovação, e isso é um coadjuvante, você não precisa necessariamente desses produtos. Vejo também pela questão das crianças nas escolas, eu vou e faço escovação, eu coloco aquela quantidade, né, necessária - "Imagina, nossa só isso de pasta, não!" - aí eles querem colocar uma quantidade enorme, então, assim, o enfoque por exemplo poderia ser diferente né, acho assim, não é no produto que você deve comprar, mas na mudança de hábitos, então é importante falar sobre a prevenção e não sobre ah, compre essa pasta ou esse bochecho, acho que o enfoque é diferente, isso não causa impacto na verdade, entendeu, então acho que falta isso, não sei se... Eu acho assim, eu trabalho nas escolas, eu acho que isso é importante, né, pra tentar... Eu percebo, por exemplo, nessa questão de prevenção mas na reabilitação dos pacientes eu percebo que eu chamo uma criança da escola que, né, a gente faz a captação das crianças com necessidades e eles vêm pro tratamento, aí eu percebo que a mãe traz a criança e ela "ai, puxa..." então veio também, então eu acabo conhecendo a família daquela criança pelo fato da criança, da escola, ter vindo fazer o tratamento, então acho que é importante pra que todos tenham o acesso, mas assim, eu acho que falta muito pra gente conseguir assim, são passinhos de tartaruga, como se fosse uma gotinha no oceano na verdade, acho que a gente tá muito longe do que a gente deveria, do que seria o ideal, mas né, eu acho que a gente não pode desistir, risos... (34:30)Acho que as auxiliares sempre pensam "nossa, ela fala demais, ela orienta demais, ela conversa demais" (risos), mas eu acho que é nosso papeel né...

P: É, você sente essa história de acharem que você fala demais?

E: Nossa!! Todo mundo fala! (risos)

P: Como que é isso aí?

E: Ah, por exemplo, eu sento e falo com o paciente "olha, é assim, assim, assim, eu fiz essa restauração, eu coloquei esse material, o que você pode sentir é isso, isso isso... O que pode acontecer ah, de repente quebra, se soltar a restauração você tem que vir rápido", então acho que são orientações necessárias, e aí "você teve uma cárie entre um dente e outro, por isso que fio dental é importante, não adianta só escovar os dentes, então eu sempre falo, sempre numa sessão eu tô falando alguma coisa, pras mães eu seeempre falo alguma coisa, pras crianças... As vezes uma criança vem, eu vejo que tem placa bacteriana, invés de falar "ah, você não tá escovando direito" eu pego e mooostro, mostro pra criança, "olha, tá vendo, se você escovar, você tira", então eu mostro pra criança, mostro pra mãe, eu converso, só que assim, é difícil viu, porque as vezes as pessoas acham que eu falo demais, eu faço plantão, eu fazia plantão, (risos) já veio um dentista e disse "Ah mas você quer fazer prevenção no plantão" (risos) , mas é uma questão de orientação né, então assim, todo mundo acha isso, as auxiliares todas com certeza acham que eu falo demais...

P: Engraçado né... Por que será que estranhem talvez?

E: Pois é né (risos), não deveria ser (risos)... É, mas eu sempre tento orientar bastante os pacientes nesses sentidos né, eu devo até falar demais né, mas eu acho importante, eu acho que é o nosso trabalho, os pacientes as vezes eles querem, de repente, "Ah eu gosto de tratar com você" mas eu acho que não é pela minha habilidade manual, ah porque eu faço... Mas é porque eu explico, eu sento, eu oriento, eu acho que isso faz diferença né, acho que assim, eu dou atenção, se ele quer conversar naquele dia eu converso, acho que a pessoa se sente melhor assim, sabe, ela se sente acolhida, eu acho isso importante... Em saúde pública é meio difícil as vezes porque eles querem que a gente atenda, que a gente atenda, que a gente atenda, só que eu acho que o acesso deve aumentar, melhorar, acho importante, só que desde que não comprometa a qualidade e a atenção que você dê para o paciente, né, eu acho importante, mesmo porque o paciente sabe se eu necessito daquele tempo pra orientar, pra fazer um tratamento adequado, ele vai, né, entender que aquilo é importante.

P: E como você acha que o acesso atrapalha esse outro jeito melhor de trabalhar?

E: Olha, é uma opinião minha, graças a Deus né ninguém vai saber, mas né, um exemplo: o PMAQ. O Pmaq ele fala, o programa de aumento de acesso, e da qualidade, só que assim, tudo bem, eu sou a favor de aumentar a quali... É, o acesso, só que até que ponto que a gente consegue aumentar o acesso para um profissional, né, digamos, eu estou aqui, eu tenho 7 consultas pra fazer mais dois encaixes, assim, eu atendo esses pacientes desde que eu consiga fazer um tratamento adequado, acho que é difícil o profissional ter aquela limitação porque quando você fala que tem que atender novos possíveis naquele período, você já determina o tempo que você tem pra cada paciente, você precisa encurtar o tempo de algum paciente pra poder atender adequadamente outro, entendeu, isso é uma realidade, não adianta, e eu, assim, eu tento, eu procuro fazer aqui um trabalho que eu faria se eu tivesse no consultório particular, eu acho que não existe essa história de que "ah, porque é saúde pública", os pacientes aqui eles sabem que, assim, que eu vou tentar fazer o melhor por eles entendeu, ah não vou fazer de qualquer jeito, só que assim, é difícil você ter que atender muitos pacientes e você conseguir dar atenção adequada pra todos eles, acho assim, se eu preciso de um tempo x pra esse paciente, eu acho que como profissional deva ter o direito de falar "olha, pra esse paciente eu tenho x de tempo", porque o acesso é importante, mas a qualidade é importante também, não adianta, então assim, eu acho que é, eu me coloco no lugar do paciente. Eu me sinto muito desconfortável quando eu vou ser atendida por um profissional e eu percebo que ele tá com pressa, sabe, isso é uma coisa horrível, imagina pra quando você precisa anestesiar, precisa fazer uma rest... Precisa esperar que a anesteia realmente faça o efeito, então assim, isso eu acho difícil entendeu, mas eu acabo assim, tentando fazer de uma forma que eu consiga tratar o paciente de uma forma adequada...

P: E como que você acha que essa... Essa menor atenção interfere nas decisões de tratamento?

E: Ah eu acho que a pessoa fica menos motivada, você imagina XXXXX, se eu chegasse pro paciente e falasse: "Senta, abre a boca, fecha a boca, tá tudo bem? Ah ok, pode marcar consulta..." - é totalmente diferente, totalmente diferente... Se eu fosse num dentista que fizesse isso eu realmente nem gostaria de voltar, eu não teria motivação nenhuma pra mudar meus hábitos, é diferente... Eu acho, eu não acho que, assim, não criticando as pessoas, acho que cada profissional trabalha de um jeito, só que eu acho assim, que você não vai ter uma melhora nesse quadro se a gente não tentar, sabe, modificar, e se a gente não aconselhar o paciente, não tentar motivá-los, isso não vai mudar também. E a gente não pode esquecer que o pai, os hábitos do pai, da mãe refletem nas crianças, então assim, eu acho importante a gente ter um tempo hábil pra gente conseguir fazer isso, e nem sempre, né... Eu entendo a questão dos gestores em relação à isso, porque a necessidade da população é grande né, a gente trabalha com uma comunidade carente, mas é importante que eu possa tratar adequadamente os pacientes... Eu tento sempre dar um jeitinho assim na agenda e tal pra conseguir mesmo né, mas não é fácil, acho que isso é uma dificuldade, mas eu não acho ruim trabalhar na prefeitura, eu gosto, me mudei pra cá e não arrependo não, eu gosto do trabalho...

P: Gosta...

E: Gosto, gosto, eu gosto assim de poder perceber melhora nos pacientes, como eu estava acostumada com crianças eu gosto assim de ir a escola, eu gosto de fazer a, a gente sempre faz a avaliação todo ano, sempre faz uma brincadeira, todo ano tem que fazer uma brincadeira diferente, ah tem dias que ah hoje é só escovação mas hoje é dia do fio dental em tal classe, então assim eu gosto do trabalho, sabe, eu tenho que fazer da melhor forma possível né, a gente tem essas limitações né, mas não é ruim trabalhar na prefeitura, é bom, eu gosto (risos), é bom sim...

**Entrevista 7**

P: A gente trabalha todo dia com cárie, e... Essa pesquisa é justamente sobre isso, sobre o trabalho com a cárie. Com relação à isso, eu queria saber o que você pensa, como você se sente, como que é sua experiência com o trabalho de tratamento de cárie, aqui na unidade?

E: Nesta unidade, é mais tranquilo, por ser um perfil melhorado... Não temos, temos somente duas famílias mais vulneráveis, então o que a gente percebe é que o pessoal é mais... É.. Vamos dizer assim, mais esclarecido e portanto a gente nota um nível de cárie n-né, um índice de cárie diminuido. É mais tranquilo, então eu acho que a cárie tá diretamente relacionada ao nível socioeconômico cultural do indivíduo. Porém já trabalhei em outras unidades bem mais em áreas de periferia e que a gente nota um índice de cáries muito mais elevado, né, o nível também das pessoas, o entendimento, o nível es- é, diretamente com a escolaridade, então... Tem um nível bem mais acentuado, um índice muito maior de cárie. Aqui, a gente nota, por exemplo, paciente vem... Uma ou duas consultas já é terminado, finalizado o tratamento, o... Em outros locais, nesses anterior que eu trabalhei que era muito mais vulneráveis, nossa, iam 10 consultas e... Muitas vezes acabavam nem finalizando, porque o paciente cansava do tratamento, tinha tanta cárie pra restaurar, tanta cárie pra fazer que, ali, que paciente até (risos) desistia do, do tratamento. Ou fazia só o que doia, o que incomodava, talvez na estética e abandonava o tratamento. Então aqui o nível melhorou muito, né. Pelo bairro mesmo ser bem melhor, né.

P: E pra você trabalhar nesses, aqui e lá, você... Você... Como você se sente com esse trabalho, você teve mais dificuldade aqui ou lá??

E: Menos dificuldade aqui. Que aqui a gente fica até mais motivado que você vê o retorno, o... A percepção das pessoas também, a gente orienta as mães que já tem uma saúde bucal boa, então a mãe é mais colaboradora, já desde pequeno, desde bebê faz os hábitos, institui os hábitos corretamente, então a gente tem muito mais facilidade. Claro que tem aqueles que a mudança de hábito é mais difícil, né, mas mesmo assim a adesão é maior, a aceitação... Comparando aos locais anterior que eu trabalhei em periferias, então a aceitabilidade difícil, sss é, a gente, nossa era uma sementinha plantada mas poucas que... Né, acabavam se... Ééé... Se modificando nos hábitos, tinha mais valor muitas vezes o que o vizinho falava do que o próprio profissional. E não só o odontológico, mas o médico, a enfermeira, o que era falado não, não tinha muita validade não.

P: Entendi, nas outras áreas também né...

E: Nas outras áreas também, não só na saúde bucal...

P: E você sentiu alguma razão de porque ser mais difícil lá, conseguiu ver alguma coisa diferente?

E: Pela própria condição social que eles se encontravam... Né, então assim, a saúde bucal ainda, infelizmente, não é nada valorizada. Né, no nosso, país, falta muito ainda essa percepção na população, principalmente mais carente, nos que tem a baixa renda. E era, era difícil eles ahh terem essa valorização e, quando a gente conseguia alguma coisa, é, nesse sentido, também não durava muito. Então acho que é relacionado ao próprio contexto social no qual eles estavam incluídos, é... As dificuldades do dia a dia, locais bem vulneráveis, bem pobres, então eles estavam mais preocupados no que iam comer, ou onde iam, onde iam passar a noite, trabalhei em locais de... Comunidades mesmo onde o chão era de terra batida, é... Barracos mesmo, construídos com tábuas, o chão... Sem saneamento básico, então... Uma casa que não tinha nem água nem esgoto... Como que, que que adiantava se dar escova e pasta (risos), não tinha água, a pessoa não tomava banho, ela ia escovar os dentes? Não ia. Então... Alimentação também pobre em nutrientes, muita ingestão de carboidratos e açúcares, e... Faltando a higienização mínima... Então, o índice de cárie, que aumentava absurdamente... E a cultura, ah, dente, ah dente depois vai lá e tira... Ainda tem muito disso nas periferias, então o que a gente nota é um índice muito alto... E essa consciência de que dente ao fim da vida vai ser tirado mesmo, depois vou lá, coloco uma dentadura e resolve o meu problema, nunca mais eu tenho dor, tá tudo certo. Infelizmente é isso que tem ainda nas periferias, muito dessa mentalidade.

P: Entendi, e comparando lá com aqui, a sua atuação dentro do consultório, em cadeira, você acha que tem alguma influência disso lá e aqui de forma diferente?

E: Tem, porque aqui num local melhorado a gente vê mais... Mais retornos, um feedback mais positivo, então assim, até acho que vira um círculo vicioso, uma coisa leva a outra, alguma mãe que não tá bem esclarecida a gente acaba conscientizando, a gente vê a adesão, aí ela volta pra uma consulta de retorno, pro acompanhamento, a gente percebe o... A dificuldade e marca de acordo com a necessidade né, do paciente ou da criança, dessa mãe e é, a gente vê que ela mudou os hábitos, mas facilmente ela aderiu, ela aceitou o que o profissional acabou indicando, isso como eu falo também, não só no odontológico, mas tanto na enfermagem, com o médico, a gente nota que uma coisa leva a outra e essa mãe né, com a criança ou o próprio paciente acaba tendo uma adesão muito maior do que nas áreas mais periféricas... Lá a gente não conseguia, tanto nos grupos pra participar, então você vinha, a criança, ou a mãe vinha e o paciente vinha, a gente resolv... É, orientava, fazia, mas lá eles procuram pelo curativo, e não pela prevenção, então quer que resolva aquele problema, aquele imediatismo, e não tem continuidade no tratamento, geralmente, quando volta, é... Pra fazer alguma outra necessidade que ele julgue, nece... É, nece... Necessária, mas não... de repente o que era o mais importante, e depois tem muito abandono do tratamento. Aí não tem a adesão, nem pra grupos, a gente não conseguia formar os grupos de fazer a sequência, o acompanhamento.

P: Diferente um pouco daqui, né...

E: Muito, muito diferente, eu notei um contraste muito grande nesse sentido, então assim, tudo o que a gente tentava lá, conseguia muito pouco de retorno positivo, então eu noto uma diferença muito grande, um contraste muito grande, relacionado com esse contexto social, sócioeconômico cultural do indivíduo.

P: Sim, sim... A gente sempre tem aqueles pacientes que... Alguns pacientes retornam sempre com, com muitas lesões de cárie nos sucessivos retornos. Nesse tipo de caso, o que você pensa sobre eles, como que você se sente com relação à esse tipo de caso?

E: Ah eu me sinto frustrada (risos). É. Muito frustrada, porque parece... É... Que o meu, o meu empoderamento pra ele não foi suficiente, né. Eu acho que... E, e é complicado porque a, o paciente, muitas vezes, vem achando que não é, também, culpa dele... Né, não vou dizer culpa, mas... Assim, ah, é assim mesmo, então eu me sinto... É... frustrada, acho que é a palavra mais certa...

P: E como você vê eles lidando com o próprio problema de cárie?

E: Eles, muitos não se preocupam, a não ser quando, quando tem alguma, algum desconforto, alguma dor... Né, é como eu disse. Aqui, por ser um bairro melhorado, tem um entendimento maior, né, então já vê alguma coisa já procura, mas sempre tem também aqueles que não colaboraram com a escovação, por mais que tenha sido orientado anteriormente, então volta com um índice de placa grande, não mudou... É, não teve mudança no hábito alimentar, né, então a gente re-orienta, então... Mas fica... É, o grau de, assim, de frustração permanece.

P: Bom, você falou de orientação de higiene, de dieta. Com relação à essas medidas preventivas, o que você pensa sobre elas, como é sua experiência com elas aqui?

E: É, é boa, é mais positiva do que no, nesses locais em que o, o paciente é menos esclarecido. Ele não está, nesses outros locais, ele não está... Muito interessado em mudanças... É... Poucos são os que aderem às orientações de dieta. Muitas vezes por falta, é, de condição financeira, né.. Ou... Ou mesmo de não gostar, de não querer aderir. Né... Já trabalhei em locais que na época a pessoa preferia, se tinha 1 real, preferia comprar... um pacote de bolacha recheada do que comprar uma fruta, fosse uma banana, né... Na época era, mais, mais valorizado o nosso real, mas porque, é... Ah eu gosto mais da bolacha recheada, então, não vou... Tá mudando isso. E aí a frustração aumentava mais ainda... Aquela sensação de impotência perante esse, esse quadro.

P: Bom na rotina sua, dentro de consultório, com relação à aconselhamento, conversar com o paciente, como que é isso aí na sua rotina?

E: É, na primeira consulta, né, a gente depois da anamnese, do, do exame clínico... Eu proponho pro paciente essa... Pergunto também como é a... A dieta, vejo a escovação, pego o macro modelo, mostro, eu falo me mostra aqui como você faz essa escovação, questão do fio dental também, mesmo aqui, um um bairro melhorado, o uso do fio dental não é diário. Então anoto daí no prontuário, né, se seja na minha ficha clínica seja no prontuário da unidade, essa, tem uma resistência muito grande com relação ao fio dental. Então eu faço toda essa abordagem, toda essa... É... Orientação inicial. Mas o paciente também, eu percebo que não quer só isso numa primeira consulta. Mesmo que ele não esteja com dor, é... Ele quer algum... É, atendimento clínico. Nem que seja pra você fazer uma raspagem, porque aí o tempo também da consulta, praticamente já , já foi (risos), né. A gente tem 30 minutos, pra cada consulta, e aí, nessa, toda nessa orientação, nessa... Abordagem inicial sobrou pouco. Então quer dizer, alguma coisa percebo que tem que ser feita, seja uma raspagem, seja uma restauração provisória, porque senão, muitas vezes, ele acaba não voltando. (17:37) "Fui lá, ela só olhou", o entendimento é esse... "Só olhou... O... Orie... Co... Nem orientação, ela conversou, falou umas coisas lá e..." Muitas vezes já aconteceu o paciente não voltar, porque não teve algo clínico na primeira consulta. Notei bastante isso. E na, nas áreas periféricas, eu sempre faço um parâmetro porque pra mim foi muito constrastante isso, piorou, se você vai lá, só orienta, só faz essa abordagem inicial, na cabeça dele, só o-só falou, foi lá só conversou não fez nada, eles nem voltam. Então é mais ainda, gritante, nesse... Contexto...

P: Nos retornos também?

E: Nos retornos também, eu vejo, eu sempre anoto, porque é difícil depois lembrar de cada um, são muitos pacientes né, eu anoto numa, num modo que eu entenda né, e eu comparo, então se o paciente houve melhora vou elogiar, vou motivar, né, muitos vem com casos de gengivite, ou até já uma perio muito avançada, então eu faço o que eu consigo aqui se eu vejo que o paciente não tá dando aquele retorno desejado eu vou encaminhar pro periodontista por exemplo, né, mas tento motivar nessa melhora e se não melhorou vamos fazer de novo a reorientação, mas sempre acompanhado de alguma coisa clínica, senão ele, percebo que ele acha que não tá andando o tratamento. Que é só a parte preventiva ali, e às vezes eu explico, "olha eu não vou conseguir restaurar, fazer as suas restaurações sem antes você tá com a sua gengiva melhorada, com sua escovação, né, melhorada, procuro... medir as palavras também pra que o paciente não se sinta... Agredido. né... Dizendo que eu "pô t-tá, vai lá e fala que eu não sei, escovar os dentes, que eu não escovo os dentes", então eu procuro ser mais sutil nessa, nessa parte mas sempre incentivando a melhora, e ainda tem alguns, a maioria acaba, né, melhoraando já na segunda consulta e tudo mais, eu mostro também, vou, removendo a placa, se tá muito o índice de placa aumentado eu eu removo "ó isso aqui", né, ninguém gosta de ver, tá comendo aquilo ali, "tá, isso aqui é que cê tá comendo" (risos), junto, né, isso aqui com a escovação pode ser removido e tudo mais, e daí o paciente parece que fica mais... Chocado e... E adere (risos).

P: Curioso o que você falou que às vezes você sente que eles ficam esperando alguma coisa além né, da... Da conversa.

E:Ficam. É, não tem esse entendimento de que, é... A prevenção acaba sendo a base de tudo, isso não tem, mesmo nos mais esclarecidos, eles querem alguma coisa que foi feito, ah foi lá pôs massinha nesse dente, fez a... Às vezes eu falo "olha eu vou deixar aqui com... Uma restauração provisória, pra o preparo pra próxima sessão e aí eu noto que, notei que tem um retorno melhor. "Ah então, hoje não é que ela não fez nada, mas ela... Eu vou voltar a mexer nesse dente, ainda não posso restaurar definitivo, notei isso, que tem essa necessidade... É, é... Curativa, junto. A prevenção funciona por ser mais esclareido, melhorado o perfil deles, mas tem que ter alguma coisa. É, eu já escutei até de... Como somos em duas colegas, é, falar "ah mas eu vim com a outra, só ficava falando, e, e não fazia nada"... Então ainda tem muito disso... Assim, de achar que você orientar, você tentar mudança de hábito, essa conscientização, é nada. "Ah vou lá la fica só conversando, só olha e não faz nada, então eu vim aqui que você já começa na primeira consulta", e... E eu, eu falei "nossa", aí eu pensei, não é que não faz nada, né, falei até pro paciente, é que há a necessidade mesmo dessa mudaaança, então passei a... A por isso na primeira consulta pra ter uma adesão maior.

P: Mas sentiu um clima ruim nisso, né...

E: É, uma, uma... Insatisfação do paciente quanto a isso, e isso nos-nos dois, tanto num perfil melhorado quanto num mais carente.

P: Certo, e mudando um pouco de assunto, no nosso dia a dia a gente passa às vezes por algumas experiências mais marcantes né, e com relação à tratamento de cárie, tem alguma delas que você se lembra que tenha sido mais marcante?

E: Hum... Me lembro de um caso dum alu... Dum dente anterior... Que, é... A pessoa vinha do nordeste... Uma pessoa, uma moça né, de uns 20 anos mais ou menos, e elaa... Veio, já não tinha o 11, e o 21 tinha uma cárie, uma classe III. Ainda não era nem endo mas tava bem grande, mas n-n-n-não viraria uma endo inicialmente. E ela veio pra extrair o dente, não pra restaurar. E eu falei, "é, mas por que você quer tirar esse dente, dá pra fazer só a restauração, tem uma cárie, nós restauramos e o dente fica... Novo... Novamente, fica bom", ela falou "não, porque o outro tinha isso, o dentista tirou lá no nordeste (risos) e foi a melhor coisa que foi feita, então eu quero já tirar o outro porque daí eu já faço um-uma ponte só"... E... Eu fiquei muito frustrada porque eu não consegui convencê-la a fazer a restauração. Não consegui e também não extraí o dente. Ela falou "não, eu quero que você tire o dente", eu falei "ah então você...vai tirar com outro dentista que tenha essa coragem, eu não vou tirar seu dente, eu me proponho a tratar"... "Não, tratar eu não quero, então eu vou procurar outro que tire", falei "então você fique à vontade porque eu não vou fazer, não foi isso... Que eu aprendi, não é a minha... Conduta", e... Mas eu fiquei muito frustrada porque eu não consegui, eu não tive o poder de convencimento pra ela... Eu pensei "como pode, uma pessoa de vinte anos, já ter essa.... Né, ment... Ter essa mentalidade, não querer... É... Pro... Se dispor ao recurso que eu tava... Propondo pra tratamento e ela queria... Depois eu não t-t fiquei sabendo, mas eu acho que ela acabou extraindo o 21 também. E eram dentes, o restante, dentes bons... E eu fiquei muito frustrada porque eu não consegui... Poupar esse elemento... Então acho que essa foi a mais marcante assim que eu tive... Nesse...

P: Com relação ao tratamento de cárie, você lembra mais algum que tenha marcado bastante?

E: Ah esse é o que me vem à cabeça (risos)... Já tive casos também de, é... Cáries em dentes anteriores... Que... Já era endo mas na época, é, eu trabalhava em outra prefeitura, que a gente fazia a endo lá, era um... Um do anterior também, dum 11 também, eu me propus pro paciente a fazer, ele tinha o 21 mas assim, eu me propus a fazer o tratamento da, do canal, depois a restauração e a pessoa, por ter já sofrido, por tar com um abscesso ali, queria porque queria extrair o dente e eu drenei aquele abscesso mas eu não extraí. Não eu me propus, você volta aqui eu marco, eu vou fazer a endo, vou restaurar, seu dente vai ficar novo de novo, e a pessoa não acreditou, essa ela já tava com um abscesso muito grande ali uma drenagem pesada, que tinha que ser feita, mas ela não, ela queria extrair... E também era um paciente jovem, né, e num se, falei, "mas você não se importa de tirar? Extrair seu dente da frente e..." "Não não, num quero nunca mais passar por isso", então a pessoa desacreditada que, um tratamento endodôntico resolveria, ela preferia extrair o dente. Outro caso que eu me lembro... Isso também nessa outra prefeitura, é, a gente atendia muitos que eram trabalhadores de safra, da cana de açúcar... E na época não era nada mecanizada a safra, então eles vinham muito também de minas, do nordeste, da Bahia, vinham pra fazer a safra. E era comum eles quererem que extraísse o dente... Com uma cárie ali pequena, uma classe I, coisa pouca, mas eles não queriam restaurar. Eles queriam tirar o dente em troca do dia de folga. Então muitos acontecia isso, vinha lá pra extrair molares que... Facilmente se resolveria com uma restauração simples, pequena até, e eles queriam a extração porque aí queria o dia da folga, ou eles tinham o dia de folga no dia seguinte, ou num dia anterior e eles queriam mais um dia em troca de tirar da extração, por conta do atestado do dia, então isso me marcava bastante e eu acabava, eu não fazia. Eles saíam bravos... Um, um dia jogou... Falou "então você me dá... Declaração que eu estive aqui", daí falei "ah mas não vou te dar do dia todo", né e eu fiz a declaração, "não vai ser do dia?", "não, não vai ser do dia, eu só posso do período que você esteve aqui", né, e aí ele jogou o papel em cima de mim e saiu bravo... Então era comum lá... Porque a extração do dente com uma cárie simples por conta do atestado do dia... Era triste, muito triste também. São histórias que... (risos) Pra nós é até chocante... E eram também, pacientes jovens, muitas vezes abaixo dos 30 anos... Dente, praticamente hígido, pouca coisa mas... Eles aceitavam que não, ééé... Não se importavam com aquela mutilação por mais que tentasse convencer... Quanto à desarmonia, né, toda que ocorreria, as consequências, eles não se importavam, então era muito triste também essa troca do dente pelo dia da folga.

P: Tem algo mais que você gostaria de comentar que não tenha sido perguntado, que você gostaria de complementar sobre o assunto, ou não?

E: É... Assim, o que eu noto, lendo artigos, pesquisas, que o índice de cárie por exemplo, até mesmo, o de (hidden), né, as pesquisas apontam uma redução... Né, acho que a redução acontece na, no índice de cárie, mas eu acho que não tanto quanto... Tem sido relatado nas pesquisas, como eu falei, a experiência que eu tenho nessa prefeitura anterior que eu trabalhei em Rio das Pedras, uma cidade mais pobre, então com muita... É, demanda, lá, de de índice de cárie, por conta até dessas pessoas que vinham, fazer parte da população, vinha de fora, esse, né, essa... É... Imigração que tinha, então eu percebo assim que, ou também nas periferias de (hidden), aqui a desigualdade social é muito contrastante, então eu percebo queee não relat... Não retrata a realidade dessas áreas periféricas, onde o índice de cárie é muito alto ainda, então tem muito trabalho a ser feito e, não só assim no curativo né, mas como prevenn... No preventivo. Percebo que também no saúde da família a gente rema contra a maré. Ainda a mentalidade, é aquela do imediatismo, é aquela do... Do curativismo. Tem muita, muito chão pela frente pra gente tar mudando isso e partir, já teve muita evolução, claro, mas eu acho que ainda tem muito a ser feito.

P: Mas isso você acha que dentro do programa ou está falando dos pacientes?

E: Dos dois, dos dois... Porque assim, eu acho que o programa veio pra ajudar e ajudar muito, mas assim, ele é mais fácil de ser, é... Assim, de correr bem nessas áreas melhoradas, nas periféricas as equipes ainda tem muita dificuldade, então eu acho que esse contraste que existe, sócio-econômico cultural contribui e, a pobreza que existe aí também, no... No povo, né, brasileiro, essa... É... Diferença de valores que, que tem, eu acho que não retrata a realidade muitas vezes que a área acadêmica acaba passando. Né, acaba, não, não é o que se vê no mundo acadêmico. Percebo isso.

P: Você acha que o saúde da família tem bastante dificuldade ainda nessa parte... Nessa áreas mais difíceis, né...

E: Mais, são mais de baixa renda, de... Áreas periféricas eu acho que sim, porque, até as agentes de saúde vem falando, elas nos trazem, né, o que tá ali na rua, embora a gente vá pras visitas domiciliares também, mas nas áreas periféricas elas tem muita dificuldade, né, de... Então falta muito ainda de, de semear, de fazer aquela semeadura pra ter uma plantinha ali que floresça, a gente tá muito longe ainda, então é muita dificuldade, tem muita cobrança em cima disso mas eu costumo falar que o buraco é mais embaixo, sabe, falta, falta aquela estrutura, imagina você trabalhar numa área que não tenha ainda saneamento básico, isso acontece, então nossa, aí você vai ver que uma coisa chama a outra e... E acaba, com-complicando, vira um nó muito grande... Né...

P: Bom, você falou que tem uma certa cobrança e cima disso né, você acha maior de que lado a cobrança?

E: Ah, dos gestores! Dos gestores, vira um efeito dominó e... Começa lá do prefeito, vai pro secretário, pros coordenadores e chega nas equipes dum modo, muitas vezes, fora da realidade, quem tá ali no dia a dia, percebe que tá, tem uma barreira muito grande. Então tem uma cobrança pra se manter no papel, aquela, por exemplo, de índice de mortalidade infantil, né, que aumentou no último ano, só que a condição que está ali foge muitas vezes do alcance da própria equipe. Então, é, e isso é cobrado como se a, a... Culpa fosse da equipe, a responsabilidade muitas vezes foge, foi feito o que era possível, enfermeira, médico, agente de saúde, todo mundo ali junto, é... Odontológico, e houve o óbito infantil, e isso não é, parece, que visto, muitas vezes pelo, pelos gestores, não é compreendido, só que, quem tá ali no dia a dia que, tá vendo, então o sentimento de frustração cresce ainda mais.

P:Tentando fazer o link de todo esse contexto e o tratamento de cárie, você acha que tem alguma interferência de todo esse contexto no tratamento, no jeito das equipes trabalharem?

E:(Silêncio)... É, sim, com relação à cárie também, é, acho que tudo contribui pra uma melhora ou também, né, ou não, essa barreira que existe, né... Mas é, acho que ainda assim... Porque assim, a adesão pro tratamento, pra eu conseguir terminar o meu tratamento, então todo esse contexto leva, né, ao retorno adequado, à pessoa conseguir vir e fazer toda essa, o tratamento... Então assim, a percepção, a importância, a valorização dela pro tratamento, "não, vou terminar", e a gente nota assim, que o paciente que não termina o tratamento odontológico, tá cheio de cárie, tá com problema periodontal, né, muitas vezes é aquele paciente também com diabetes descompensado, com a pressão lá na casa do chapéu, então é, parece que aquele polarizado, né, que tem todos os problemas estão ali, que ele não tem aquela adesão, não só no bucal mas também na parte médica, sistêmica dele, acaba não aderindo, então... Agora, quando tem todo uma equipe ali que consegue fazer, eu acho que tá tudo correlacionado, né, e aí o paciente vem pro tratamento, vai finalizar e acaba, claro que resultando né, num, num... Tratamento concluído e diminuindo o índice de cárie, então acho que é tudo correlacionado com certeza.

**Entrevista 8**

P: No dia a dia a gente trabalha com cárie, e essa entrevista é justamente sobre esse assunto. Eu queria que você me disse como você se sente, o que você pensa, como que é tua experiência aqui com o tratamento de cárie dentária?

E: Experiência... Humm, é, a gente faz restaurações né de amálgama, resina, ionômero, né... O que mais, o que mais a gente faz aqui é restauração, né? Remoção-Remoção da cárie e restauração, todas-todas as idades, né, desde criança até mais velhos, todos... Todo-todo-todo dia tem né, tod- o que mais tem é, o que mais faz aqui são restaurações.

P: Bom, como que você se sente com esse trabalho, você gosta de trabalhar com isso?

E: Ah eu gosto, eu gosto, a gente vê o resultado na hora, ali né, um tratamento fáci-fácil de fazer assim né, que resolve né, que resolve o problema, eu gosto sim.

P: Bom, a gente sempre tem alguns casos de pacientes que quando eles retornam, retornam sempre com novos problemas de cárie, né, com relação à esse tipo de situação, o que você pensa sobre isso, o que você sente com isso?

E: É, isso aí é meio desmotivador, é a parte mais difícil assim é mudar hábito, né. Mas aqui assim, nesse bairro assim até que nã-não tem muitos casos assim não, viu? Maioria que a gente t- a gente trata assim resolve, são-são poucos assim que a gente vê que não se cuida, né... Isso acontece mais assim com, com raspagem né, que a gente faz a limpeza, pouco tempo depois já volta com bastante tártaro de novo né, mais complicado assim é mudar hábito, principalmente paciente mais... Mais velho né... É, é algo que é, a parte mais complicada é essa.

P: Você acha que mais velho é um pouquinho diferente?

E: Ah é mais difícil né de faze- orientar a escovar, é, criar o hábito né, passar o fio dental, tal, é mais difícil né, criança é mais fácil.

P: Você acha mais tranquilo com crianças?

E: Acho. Pôr esse hábito né, mudar o hábito, eu acho mais fácil.

P: E com as crianças como que é a experiência aqui?

E: Ééé, a gente faz escovação na escola e na creche toda semana né, orienta no consultório também a fazer escovação, passar o fio dental, orienta os pais, criança muito pequena a gente orienta os pais como escovar, orienta escovar principalmente à noite antes de dormir, passa as orientações de di-dieta também, né...

P: Na escola como que rola lá?

E: É, na escola vai... A auxiliar, auxiliar de saúde bucal, mais o estagiário, vai toda semana ou na creche aqui ou na escola, aí faz escovação com flúor em todas... Em todas as crianças, duas salas por semana né, não faz em toda a escola toda semana.

P: Sobre esse trabalho de aconselhamento e orientação por paciente aqui né, em clínica, relativo à cárie, como que é sua experiência com isso aqui no consultório?

E: Ah... Aconselhamentos?

P: É, aconselhamento, orientação, a parte preventiva aí de cárie, como é que costuma rolar isso aqui na unidade?

E: É, caso a caso, aqui assim não tem um, não tem um grupo assim específico pra isso, né, tem só o grupo de gestantes que esse ano não tá tendo, a gente passava essas orientações, mas é no-é no consultório mesmo, ano passado também fui no grupo da caminhaaada, e passei lá orientação de cárie, é... Gengivite, periodontite nesse grupo.

P: Grupo de caminhada da unidade? Como que é, eles vão fim de semana?

E: Não, não, todo dia, é... Caminhada é terça, quarta e quinta se não me engano, 7 horas da manhã, eles fazem ali no zoológico.

P: Foi legal, como é que foi?

E: Foi, foi legal, eles perguntaram bastante, demonstraram, demonstraram bastante interesse, né... É um público mais homogêneo, são todos mais... Pessoal de mais idade, então a gente pode passar... Orientação da mesma forma assim que, todos entendem...

P: Como você vê os pacientes lidando com o próprio problema de cárie deles?

E: (silêncio) Bom... Varia bastante, é, tem uns que não se importam, né, já tem ou já tiveram um monte de cárie, tem um monte de cárie, não tão nem aí, tem uns que falam que tem uma cariezinha já fica assustado, né... Varia bastante né, de um paciente pro outro...

P: Bom mas e a parte de eles tocarem em casa né, como é que você tem visto isso? Deles manterem, se cuidarem, você tem alguma percepção nesse sentido?

E: É, percebo que a maioria assim dos pacientes aqui se cuida bem assim, são poucos casos assim dos mais desleixados, é... Assim, é, às vezes é usuário de droga né, os casos são esses né, que não se cuida... Bebida, né, alcoólatra... Maioria das pessoas a gente percebe que se cuida, assim...

P: No nosso dia a dia a gente passa por algumas experiências que são mais marcantes. Com relação à cárie, né, você se lembra de alguma experiência mais marcante que você tenha passado durante o tempo que você tá no saúde da família?

E: O que-que assusta mais, assim, impressiona mais assim são, às vezes que aparecem, mas pouco, às vezes aparece criança pequenininha 4, 5 anos com muitas cáries, mais de... Mais de 10 dentes cariados né, isso aí que-que assusta e é mais difícil tratar também né, criança pequenininha assim com muita cárie... Vai ter dor né, então bem complicado, né... Mais, mais impressiona, mais incomoda assim porque é mais difícil resolver, né... E também não adianta só restaurar, que... Não mudar os hábitos vai cariar rapidinho de novo. Nesses caso assim maioria a gente acaba... Encaminhando pro ceozinho, tem especialista, lá eles fazem o tratamento...

P: De adulto você já chegou a pegar alguma coisa bem... Bem assim, que chame mais a atenção com relação à cárie?

E: Ah já né, aparece, aparece alguns casos em adultos com vá-várias raízes residuais né, várias extrações indicadas, né... Aparece também de vez em quando, né... Assim, com relação à criança é mais fácil de resolver aqui vamos dizer assim porque o que eu vou fazer aqui é... Nos casos que tem muitas raízes residuais é extração né, depois assim o SUS não... Só se for extração total que o SUS vai resolver aqui em (hidden) e fazer uma, uma prótese total né, senão tem que encaminhar pra algum outro serviço pra fazer uma prótese parcial, né... O que a gente faz aqui no, no PSF assim seria... Não seria difícil resolver que seriam as extrações, restauração em adulto né, então, o problema é reabilitar depois né, fazer a prótese é que é... É complicado né, já não vai fazer por aqui. Só total, por enquanto aqui em (hidden) é só total.

P: Com relação à parte de aconselhamento, orientação, no dia a dia tem algum tipo de dificuldade com esse momento em específico ou você acha que transcorre tranquilo, como que é?

E: Varia muito de paciente pra paciente, tem paciente que... Que entende bem o que a gente tá falando assim né, agora tem paciente que tem muita dificuldade em entender né...Por mais, mais fácil que a gente tente falar, mais... Facilidade, simplificar, não usar muito termo técnico, mesmo assim tem pacientes que tem dificuldade né, então varia muito de paciente pra paciente também... Então tem que tomar cuidado com paciente que a gente vê assim que não tem, não tem assim... Escolaridade, não tem... Estudo nenhum né, usar palavras bem... Bem simples assim pra eles entnederem, né... E também vai muito do interesse do paciente, tem paciente também que não, não tá nem aí né, a gente tá falando e... Não dá importância praquilo.

P: Esse tipo de paciente você acha que é mais comum ou incomum?

E: É mais incomum, é mais incomum, a maioria entende bem o que a gente faaala né, se impooorta com o que a gente tá falando... Tem exceções assim de paciente que não se cuida, que não, não tão nem aí né, não vão mudar hábito, tal, só vem no dentista quando tá com dor, uma coisa incomodando mesmo assim, mas a maioria... A maioria colabora sim.

P:Você acha que tem algum perfil assim que, eles são geralmente de algum certo perfil ou...

E: Assim, na maioria das vezes são mais...Carentes... São pacientes que tem um problema com álcool, drogas, né... Na maioria das vezes são pacientes mais jovens também, assim né, adultos jovens... Mais ou menos esse o perfil, assim... Entre 20, 20, 40... São mais desleixados... Mais difícil ver paciente mais idoso, descuidado assim... E criança, assim, entre 10 e 20 anos eu vejo que a maioria até se cuida mais, assim... Menos de 10 anos, aí a responsabilidade é dos pais, né...

P: E com os pais você falou que rola mais tranquilo essa parte de orientação...

E: É... Varia muito de pai pra pai, tem pai que você vê que não... Não cuida do filho, da criança né, você vê que a pessoa não tá muito... Muito a fim de ficar ouvindo aquilo ali, né... Varia bastante também, pais com bastante zêlo, zêlo até demais né... Varia muito...

P: Esse tipo de caso que é mais complicado de ter adesão do paciente, você... Isso de alguma forma te desgasta mais ou é tranquilo?

E: Ah um pouco assim, a gente fica meio, é, assim, é... Sente que o trabalho não tá sendo valorizaado né, fica um pouco, um pouco... Meio assim decepcionado né, mas faz parte... Lidar com gentes bem diferentes né...

P: Da época que você começou pra hoje você sente alguma diferença com relação à isso, à tua percepção com esse trabalho de cárie?

E: Assim, eu percebo que eu tenho... Bem mais facilidade pra fazer o tratamento, parte técnica, quando eu entrei eu era recém-formado, né... Assim, é... Tenho mais facilidade pra diagnóstico, do que é... do que vai precisar fazer canal, do que não vai, tenho bem mais facilidade dessa parte técnica, né... Até mesmo, é, diagnóstico de cárie, o que é, o que não é cárie, o que precisa restaurar, o que não precisa, mudou um pouco assim, né...

P: E com relação à esse relacionamento com o paciente nesse sentido, de aconselhamento, orientação, como que era no passado e como é hoje?

E: Eu acho que antigamente era assim mais parecido assim com todos os pacientes, era a mesma, vamos dizer, a mesma orientação pra todos, hoje acho que... Cada caso é um caso, já consegue perceber mais a diferença entre os pacientes, né, entre aqueles que se importam que vai valer a pena você... Orientar, tem aqueles que você... ficar passando orientação vai ser perda de tempo... Mudou nesse sentido, você consegue perceber mais... Paciente que se importa com isso ou não...

P: E quanto ao desgaste, você sentiu alguma diferença? Desgaste assim, de mexer com você, de te incomodar...

E: É, hoje me incomoda menos, assim, essas coisas, se vem um paciente que não se cuida, vêr que o trabalho não tá... Não vai dar muito resultado, hoje me incomoda menos do que quando era recém-formado.

P: Algo mais a ser comentado que não (...) - Não.

**Entrevista 9**

P: Bom, a gente trabalha com cárie, todos nós dentistas... E essa entrevista é justamente sobre isso. Eu queria que você me disse como você se sente, o que você pensa, como que é tua experiência aqui com o tratamento de cárie dentária?

E: Então aqui, bom, vou falar daqui porque faz tanto tempo que eu trabalho aqui que eu já não sei como que é em outro lugar, né... Então XXXXX, então aqui é o seguinte, quando a gente entrou aqui na, na rede pública, é... A gente conta com... ... vários fatores aí, né, é... Aqui na parte mais... Periférica, aqui não é tanto ainda, mas quando, né, há alguns anos atrás ainda era quando não tinha tantas construções aqui... A gente percebe que a população não tem conhecimento do que é cárie, às vezes nem a gente tem direito, nem tem direito do que é cárie né, mas a população eles não... Eles não tem muita noção do que, de que cárie é uma doeeença, que é transmissííível, tal, e que, o que provoca a cárie são duas coisas aqui que eu acho fundamentais, é... Tsc, o nível, a parte cultural e a parte de alimentação. Né, então isso aí é muito complicado de você... É... Direcionar alimentação, e cultura, cultura de prevenção, né, pra... Pra população. Então o que foi feito aqui foram... Trabalho preventivo foi feito nas escolas através do, do... Do programa da, municipal de saúde com os dentistas, com os técnicos de higiene dental, eles fizeram, eles fazem uma, uma escovação em maaaassa, em períodos estabelecidos né, orientação, paleeestra nas escolas, pra que as crianças, elas escovem os dentes pelo menos na escola. Tem, tem trabalho que são feitos semanais quanto a... com relação à isso e percebe-se às vezes que a criança só escova lá, naquele momento em que vai o pessoal escovar. Então é assim, a gente percebeu aqui que... É... Esse, essa parte cultural e alimentação é meio complicado de trabalhar, mas fazem uma coisa bem bacana ainda e, o que percebe-se em relação ao tempo é o flúor na água né, a fluoretação da água que diminuiu muito o índice de cárie dental... É... Em relação à essa parte cultural e alimentar, então combate-se a cárie nas torneiras (3:54) né, e ... Isso ajuda bastante, mas ainda tem bastante... Tem bastante índice, né... Não é uma coisa assim como era há muito tempo atrás, mas eu acho que é mais pelo fato da fluoretação, da fluoretação de água mesmo né... É, a parte educatiiiiva, com, com relação à má escovação e prevençããão, é feita bastante, inclusive alimentação também mas é complicado né meu, você vê que a mídia ela acaba com tudo o que cê fala né... E outra coisa que acontece também é que você às vezes faz uma palestra pras crianças né, adolescentes, e chega em casa os pais... Dá um pacote de, de salgadinho, aqueles "gorduritos" né, aqui é muito comum você ver criança esperando, pra ser atendido, com pacotão de... Joga né, a mãe joga e fica quieto aí e boa, e come e fica quieto, aí vamos ver as consequências... E aqui, hum, é, você vai fazer compra no supermercado, tem aquelas coisas de pirulito né, nos caixas ali então... Pai com criança no colo já pega um pirulitão, a criança nem sabe o que é aquilo, já soca... Então esse tipo de coisa é meio com... Complicado de combater. Então é... É assim, fator alimentar aí é, muito complicado, porque até a televisão joga uma coisa a gente joga outra... E é difícil de combater né...

P: Você falou um pouco dessas questões externas né, e isso dentro do consultório como que você vê isso?

E: É, aqui dentro do consultório a gente vê que... É, é... Tsc... O que resolve bastante são os trabalhos nas escolas e a fluoretação de água, né... Então a gente tem, teve uma diminuição drástica nos níveis de cárie aqui, o índice CPO-D né? Teve uma redução muito, muito grande né, mas é mais em relação à esse tipo de coisa que é feito externo, né... Mas a... Os trabalhos de prevenção aqui são feitos. Principalmente com a... O pessoal da, da saúde bucal né, que roda as escolas aí, é feito de uma maneira muito eficiente, né? É... Essa parte de educação... Agora aqui dentro, cara, assim, o que que a gente tem aqui. É, o público nosso aqui de, de... Tsc... Mais forte, são mães... Que não, não trabalham né, pelo fato do horário de funcionamento da unidade né, os, os maridos assim eles... É, geralmente eles vem fazer o tratamento mas tem que pegar um atestaaaado, tem que vim nas férias, tem que vim... Tipo, o cara trabalha a tarde e a noite aí vem de manhã, trabalha a noite e de manhã vem na tarde, né... E... As crianças, é, tão estudando mas, é, tem... Tem um... Um... É, uma demanda muito grande aqui. Assim, o que eu percebo aqui dentro, XXXXX... É o seguinte... É... Existem muitas políticas de, de... Prevenção pra crianças. Né... Só que a grande dificuldade minha aqui é mesmo a hora que chega na adolescência, eu não vejo nenhum tipo de programa pra adolescente. Então é... É... Eles tem, as crianças tem uma saúde bucal boa e... Chega na adolescência e... Falta um programa e eles, tipo que abandonam aquela, aquele trabalho preventivo. Por que você vê aqui, por exemplo, acabei de marcar aqui... Trêêêss miiil pessoas, aqui eu atendo, isso do Jaraguá 1, que eu atendo duas equipes, então na verdade são seeeeis mil paceintes que a gente tem aqui, é lógico que tem muita gente que tem plano de saúde. E que não vem aqui tratar, mas o plano de saúde às vezes é só na parte médica, na parte odontológica não tem muita coisa de plano. Então são seeeis mil pessoas. É... Então acho que seria legal ter mais um profissional na área aqui pra... Ajudar nessa parte preventiva, porque a gente faz todo o... Trabalho externo aqui de... Nas escolas, a gente vai uma vez por mês fazer escovação, mas a... Os, as agentes de saúde, tanto aqui quanto do dois, eles vão toda semana, né, fazer esse trabalho de escovação, nas escolas. Aqui a gente tem na, na verdade são, são 3 escolas aqui que a gente toma conta, que é o Euclides Buseto, a creche lá e essa creche aqui do lado. As outras escolas já não fazem parte, como não é, PSF né, regionalizado, aí depois, quando ficam, quando virou PSF aqui melhorou muito né, porque como regionalizou a gente tem noção de tudo que acontece em tudo quanto é lugar. Principalmente na parte de saúde bucal- quando era unidade básica, a gente não tinha noção nenhuma do que acontecia, nada, porque hoje tem as agentes de saúde que fazem todo um relatório. E a saúde bucal tá incluída nesse relatório né, então a gente sabe aonde tá, tá com mais problema, qual família que tá mais vulnerável, então isso é bacana. Então a gente faz esse trabalho, tem uma família que tá muito vulnerável vamo lá, vamo conversar com eles particularmente. Né... Na época da UBS a gente não sabia de de de, a gente não tinha dado, não sabia quantas famílias tinham, quem era vulnerável ou não, o pessoal vinha eu atendia e ia embora, né, e com-como virou PSF aqui melhorou, essa parte de acolhimento melhorou muito. Inclusive, é... Em relação à saúde bucal também. A gente faz um trabalho preventivo melhor. O que peca ainda cara é a alimentação. Alimentação é o "X" da questão, porque... A... Bebezinho a gente faz muito trabalho com bebês né, ah o bebezinho você põe, você dá mamad... É, dá açúcar pro nenê? "Não, não dou, dou leite Ninho", "Dou (sei lá), farinha láctea"... Como se não tivesse açúcar, né. Como se o açúcar fosse aquele saco branco escrito "açúcar refinado" só. Então ainda bate bastante ainda nessa parte de alimentação. Né... Mas assim, melhorou com, com relação à mudança pra PSF melhorou bastante, inclusive a gente tem mais, é... A gente tem muito mais informação sobre o estado de saúde geral do paciente. Né, então não é só... É... Boca... Não é, a gente tem muita interatividade aqui médico, enfermeiro, agente, tem todo um... Os agentes de saúde fizeram treinamento com a Colgate, acho que deram uns kits aí, elas tão bem motivadas nessa área... Então isso aí é um trabalho meio a longo prazo, mas... Ficou bem legal. Ainda a alimentação ainda eu acho que... Problema.

P: Com relação à aqueles casos de consultório que... Paciente seu de bastante tempo e tem alguns casos que eles sempre reaparecem com cárie, sempre reaparecem com cárie, com relação à esse tipo de caso, o que você pensa sobre eles?

E: Então, tem paciente assim cara, tem paciente que não tem jeito né, eu acho. Que... É aquela coisa né, o , o... Que eu falo de adolescente, você passa da infância pra adolescência, (11:01), se você se perder na curva você não volta mais. Muito difícil né... Enquanto você é criança você ainda pode orientar de uma maneira legal, tal. Agora, tem paciente assim, principalmente adolescentes, que não... Não tem jeito cara, ele... Termina o tratamento, "quarenta e cinco" cáries, ele volta, com "quarenta e oito"... E assim, vai, vaaaai na casa, conversa, não tem jeito. Tem muita gen... Tem muita coisa que influi, por exemplo... Droga né, droga é problema. Crack, tem muito aqui. Rola muito crack aqui no bairro, então... Esses adolescentes que se perderam na curva, eles não, é muito complicado de fazer um trabalho com eles né. É, parte de saúde bucal, eu converso, fala, pá, tem um paciente meu aqui por exemplo que ele começou a tratar com 13 anos ele já não tinha nem um molar mais. Hoje com 18, 19 já usa prótese total. Não tem jeito o cara, sabe, fomos lá, conversamos, vinha com a família, palestra, orientação... Não deu. Mas é assim, é, é... Eu falei pra você, são seeeeis mil pacientes, tipo, uns 50... Não dá nem 10% de pacientes que são "problema". Agora tem aqueles que são muito bacana, né, tem aquele que você termina... E passa um, dois, três anos, volta, alguns voltam nos retornos e tá tudo bem. Tem nada... É outro oposto... Vai depender da famííília, vai depender muito da, da orientação que tem né, mas assim, é bastan... Os casos são bastante... Satisfatórios, né...

P: E desse tipo de caso que são meio refratários assim, você falou de adolescentes que são problemáticos assim nesse sentido de drogas, tal, e você tem alguma experiência disso com adulto, por exemplo, que não esteja nessa situação?

E: Adulto que voltou com recidiva? Ah tem... É, são poucos os casos viu, não são muitos, não tem uma coisa muito... É assim, o paciente que dá trabalho ele vai dar trabalho sempre. É... A gente bate assim numa, numas teclas assim que... A gente conversa muito nas reuniões o que fazer né, com esses pacientes aí que dão muito trabalho. Tem uma turma que acha que tem que deixar, que não tem jeito... Tem uma turma que acha que o paciente "bom" que você tem que se dedicar, aquele que não, o paciente que tá a fim. Ele tá a fim de tratar você se dedica à ele, não... Outros achão que não, que aquele que tá a fim vai tar sempre a fim... É assim, eu, eu vejo... É... Quando, quando o paciente não tá envolvido com droga, não tá envolvido com nenhum problema psicológico, e... Volta com muita recidiva é falta de cuidado, né... É... O que tem muito também XXXXX, às vezes o paciente mora aqui e muda de bairro. Aí relaxa. Aí no outro bairro não tem dentista, por exemplo. Aí ele não pode vim aqui tratar. Aí ele fica um ano, dois, e volta pra cá. Volta pro bairro, aí já tá... Já desequilibrou tudo. Né... Aí vai depender muito da onde ele for, que tipo de acolhimento ele vai ter lá, não que aqui seja bom ou ruim tal mas, a partir do momento que virou PSF isso estorou muito aqui, né...

P: E como você se sente quando chegam esses casos aí, isso...

E: Recidiva? Mal, muito mal. Se o cara mudou de bairro, não tem o que fazer, né, agora, se ele tá aqui, às vezes acontece o cara tá aqui, é, é... Volta, depois de um tempo tá com coisa pra fazer, é... Então a gente se sente meio que... Não deu nada certo do que a gente fez, né... É, não, não, pô, a gente tá aqui fazendo o quê, né? Eu fiz uma vez um trabalho, cara, lá em São Paulo na UNIFESP, da especialização, mudou, a gente, o, o que eu vejo aqui, XXXXX, assim, o dentista ele é meio, ele é um cara meio complicado de se inserir nessas equipes aí. Porque... É, não só dentista, mas o dentista muito, ele tem uma formação muitoooo, voltada... Pra serviço particular. Né, pelo menos não sei como tá agora, mas na minha época, né, eles, eles não tem uma... É, uma orientação muito... É... é... Muito grande ou muito eficiente pra serviço público. Então quando ele chega num PSF, num programa de saúde da família é difícil dele se inserir, nesse tipo de coisa. Então muitas vezes, a... A falha, é do profissional. Quando eu entrei aqui em UBS, é... E virou PSF, eu vi que a falha era minha, de não me inserir na equipe. Porque eu não tinha, eu não tinha noção do que acontecia, às vezes, o problema ambiental do cara é um, sei lá, é um emprego que ele perdeu, é... A mulher dele foi presa... Então pô, ele descuidou por problemas psicológicos da saúde da boca. Então a gente não tinha muito essa noção quando virou, quando teve essa transição, na época da mudança. Então ai com o trabalho de PSF a gente foi identificando alguns fatores, por exemplo, é... Desemprego... (16:03) Tinha gente que se sintia deprimido porque não tinha escola, não tinha coleta de lixo, não tinha ponto de ônibus... Então o... A, a coisa era muito mais, não era um descuidado com a boca porque... "Ah não quero escovar o dente". Mas porque existiam outros fatores fooora, que... Né, que influiram nessa, nessa decisão da pessoa não se cuidar. Então mas isso aí a gente trabalha muito também. Né, essa parte psicológica, por exemplo, conseguir... É... Coleta de lixo na rua da pessoa, daí, pessoal daí, do centro comunitário, vão atrás, a gente identifica o problema e eles vão ver se, se vai resolver ou não, aí já é... Mas vai, e o, o paciente, o tsc, ele fica feliz, só de correr atrás das coisas dele, né... Então teve muito disso também XXXXX, com todas essas partes, esses fatores psicológicos, por fora, que não tem nada a ver... Quer dizer, tem a ver. Mas a gente não tinha essa visão. E acho que as faculdades hoje elas não, eu não sei como que é por aí mas essa, essa visão a gente só aprende fora mesmo. Que é muito voltada pra... Consultório particular e... Sei lá, pessoal quer só ver a parte financeira, aí não é... Não é só por aí.

P: Comento sobre algumas questões da formação, e pergunto sobre receber os alunos de graduação lá pra estágio.

E: É então, exatamente, veio, sei lá, acho que umas... Umas duas semanas, vieram... É, sei lá, uns 5, 6 aí (alunos), era um só que achou legal e outros, "ah não, isso aqui eu... Eu vou me formar e montar consultório". É, não sei como é que tá, mas a gente via muito disso né, as pessoas se voltando... Não se voltando pra isso, pra esse fator integral da saúde bucal. Olhar o paciente como um todo e não só a boca, né, mas a gente via o... Muita gente aí só preocupada mesmo em pegar o diploma e partir pra área particular, né...

P: Você comentou um pouquinho sobre isso mas como é que você vê o paciente lidando com a, o próprio controle, a própria questão da cárie?

E: O paciente? É, em conhecimento ou em cuidado?

P: O que você achar mais importante (risos)

E: O paciente, é... Hoje ele tem... É, assim, tsc... Quando se fala em estéti... O, o paciente ele tá muito preocupado com estética, né, então assim, se ele tem cáries, por exemplo, nos dentes da frente, ele procura mais do que no fundo. É... Ele, o, o conhecimento dele sobre a doença cárie é muito, não é tãooo... Depende do, depende muito do que ele tá procurando também, do que ele quer saber, a gente procura explicar nas palestras tal que cárie é uma doeeença, contagioooosa, é muito comum "ah, não posso usar a escova da minha mulher" não, não pode, porque é uma doença que pega né, agora em relação ao paciente, assim, é... Ele tá muito, hoje com essas mídias, ele tá muito mais esclarecido né... Agora, eu acho agora que tem coisas, por exemplo, que... A gente tem que bater de frente, tipo pastas que fazem clareameeento, né... É... Não tem muita divulgação com relação ao fio dental... Então é mais... É mais assim, o pa, o paciente ele tá interessado, é... Muito na estética e quando dói, né, agora o tratamento preventivo né, assim, de vez em quando, hoje melhorou muito, qunado tá bem. Eu tô bem então eu tenho que procurar o dentista, né... Melhorou muito né, mas a pouco, a não tanto tempo atrás era só... Curativo e quando tava com dor, curativo e estético no caso né, mas hoje, hoje tá bem mais... Hoje tá bem mais esclarecido com relação da prevenção né. Mas eu acho também que a faculdade faz um trabalho legal quanto à isso né, a parte de preventiva e... E... Aqui em (hidden) a gente tem uma vantagem né cara, você tem a faculdade, você tem o CEO, e você tem os PSFs né... Que não é todo lugar que tem isso tudo integrado né... Mas assim eu acho que em relação ao paciente sim, eu acho que... É... A gente procura esclarecer mas eles não... Eles ainda não entenderam que a boca é um todo. Né... Ainda... T-tão começando a entender, né... Que a boca é um todo e que cárie é uma doença. Né, não sei se é isso que você queria saber...

P: E você comentou sobre um caso que te marcou, sobre a prótese total, com relação à cárie tem mais algum caso aí que cê lembra que tenha te marcado bastante, que tenha sido interessante assim?

E: É, em matéria negativa foi esse aí, né? Ah mas aí tem muitas coisas positivas aí né, você vê pacientes que não, que eu trabalho aqui faz tempo, e... Pacientes que tinham, sei lá, 5, 6 anos de idade e hoje tão adultos né, aí passou essa fase sem nada. Ah tem bastante caso assim, tem bastante... Passou sem nada... Isso é gratificante, né! O que a gente costuma fazer é dar um prêmio, dar um presente, não é barganhar... Né mas pô, o cara tá aí, bom vamos fazer um kitzinho de escova, pasta de dente... Fio dental, né... Só pra fazer um agrado aí, costumo fazer bastante isso aí... Mas assim, na parte negativa foi esse mais, e... Agora na parte positiva tem bastante caso aí que passaram... Sem nada, sem cárie, sem doença periodontal... Casos legais, tem bastante...

P: Com relação à esse assunto tem mais alguma coisa que eu não tenha perguntado que você ache interessante comentar?

E: Não, tá tranquilo!

**Entrevista 10**

P: Bom, a gente trabalha com cárie, todos nós dentistas... E essa entrevista é justamente sobre isso. Eu queria que você me disse como você se sente, o que você pensa, como que é tua experiência aqui com o trabalho sobre a cárie dentária?

E: (silêncio) Então, a cárie, até onde a gente estuda e é passado pra gente, uma multifatorial, né? (...) Causada pelo hospedeiro, a microbiota oral e a dieta, né? E... Com relação à prevenção, a gente trabalha em cima dessa tríade... Em relação ao diagnóstico... É visual, a gente usa a sonda exploradora, bom senso e opinião do paciente... (silêncio)

P: E no dia a dia, a gente tem alguns casos de pacientes, alguns pacientes que... São pacientes nossos de rotina, tal... Alguns deles sempre voltam com novas lesões... Com relação à esse tipo de caso, como que é pra você, como que você se sente, como que é sua experiência com isso aqui?

E: É, como a gente falou na primeira pergunta, por ser uma doença multifatorial, ela depende do comportamento do paciente pra ter recidiva ou não. Então se houver uma boa higienização, uso do fio dental, escovação adequada, tendência é diminuir. Caso ele não adote esses hábitos, a tendência é que mantenha o fluxo, né... Aí chegando aí a gente trata o que pode tratar aqui... O que for mais complexo e a prefeitura não fizer a gente encaminha.

P: E no dia a dia, de dentro do consultório, como é que costuma, esse tipo de caso é frequente, não é, ou... Desses pacientes que são... São meio refratrários ao tratamento?

E: Acho que a média padrão em qualquer lugar é... Sempre tem, os casos de sucesso e insucesso né, todo profissional tem...

P: Você se sente de alguma forma, mal por causa disso? Mexe com você ou nom momento você acha que tá tranquilo?

E: É, a gente faz o que tá ao alcance da gente. O resto... É com Deus... (risos)

P: Na experiência do dia a dia, como é que costuma rolar essa parte preventiva, como é que você vê isso?

E: (Silêncio) É então, a... A parte da prevenção a prefeitura faz a parte da fluoretação da água né... A gente indica... A escovação, o uso do fio dental pro paciente e... E a pasta de dente acho que todos hoje tem flúor também né... Então, o que tá ao alcance a gente tá fazendo... Só pra criança mesmo que às vezes contraindica porque eles ingerem, né... Ou se for usar fluoretada usar pouco, né...

P: Sei... E da tua rotina assim você lembra de alguma... Algum caso, alguma situação clínica que tenha te marcado bastante com relação à esse assunto de cárie?

E: (silêncio) Olha, não aquiii em (hidden), mas lá em minas uma vez um paciente apareceu... com... início de dor de dente, ao exame clínico aparentemente não tinha nada... Era só o sulco que tava... Um pouquinho escurecido, mas a sondagem, sondava e não... Não tinha nada, mas aí hora que abriu tinha lesão. Isso que eu falo que, às vezes eu sempre questiono com o paciente... Ó, tem, o sulco tá escurecido, não prende a sonda, quer abrir a gente abre. Não quer a gente proserva, acompanha. Mas foi isso...

P: Esse caso ele teve alguma... Algo mais, ele parou aí daí resolveu, como é que foi?

E: Então, eu fiz a restauração, padrão normal né, forrei com hidróxido de cálcio, coloquei ionômero de vidro, depois coloquei o amálgama depois logo em seguida eu mudei de lá, então... Eu não sei se evoluiu, porque eu falei que podia evoluir pra canal, quando é muito fundo, a exposição pulpar, né... Então eu não sei se evoluiu ou não. Porque eu mudei de lá, eu não... Não deu pra acompanhar. Mas quando aparecem casos que tem restauração profunda aqui, eu sempre aviso o paciente que pooode dar endo, né, a gente sempre alerta eles.

P: E esse caso em específico que você falou do sulco, por que que ele te marcou mais?

E: (Silêncio) Então, não, é porque olhando assim aparentemente cê sondava e não tinha nada, eu era recém formado também né, (10:48) e aííí... Eu tinha, na minha boca... É... Lesões semelhantes, de cárie incipiente, o professor da faculdade falou que era melhor não mexer... E aí eu tomei isso como referência e tá até hoje as minhas... Né, não, não deu nada, eu vou no dentista ele olha, acompanha, aparentemente está tudo bem... E o caso dele que era bastante semelhante não, a sonda não prendia, não tinha orifício, nada... Deu isso, né... Um paradoxo, né...

P: E... Na tua experiência do dia a dia aqui, como é que você vê os pacientes lidando com o próprio problemas dele sobre a cárie?

E: Então, eu sempre tive comigo que o problema da cárie, é... Ele tem que ser compartilhado. Como a gente falou na primeira pergunta, por ser uma doença multifatorial tem o fator do paciente, né... Se ele não escovar, não usar o fio dental, milagre a gente não vai poder fazer, né... Então a gente tenta limitar o dano... Chega, pega o estágio que tá, o que eu consigo fazer eu faço, o que eu não consigo eu encaminho...

P: E esse tipo de orientação que rola no dia a dia, isso costuma ser tranquilo, sente algum tipo de dificuldade, uma tensão aí, como é que funciona?

E: É, em geral eu explico, eu falo olha, é... A gente vai até onde a gente tem a habilidade técnica né, pra fazer determinado procedimento... Eu fiquei parado um tempo né, quase não me dediquei à dentística na minha... Carreira, então algumas coisas realmente pra mim elas... Elas tem um grau de dificuldade talvez maior do que pra outro profissional. Mas o que eu consigo fazer e eu vejo que dá pra fazer eu faço, eu ajudo, eu me disponho a sempre servir da melhor maneira possível.

P: Com relação a você trabalhar com paciente essa questão de orientação e tal, tem algum tipo de coisa que pega mais no dia a dia, que incomoda?

E: Não, não, é tudo tranquilo, tudo tranquilo, a maioria às vezes fala não, deixa que eu... Eu me viro, eu dou um jeito... Obrigado pelo que você já fez... Percentual baixo é que fala "Ah eu não tenho condição, então queria tirar o dente", e... Esses dias tinha um que eu falei "olha, às vezes a restauração aqui vai ser complicaaaada, tá a nivel da gengiiiiva, pode ter sangramento, às vezes precisa de um profissional mais habilitado pra fazer... Ele falou "ah, eu não tenho condição, e se for pra me dar problema depois eu prefiro que extraaaia"... Eu falei não, tenta, "não, não quero, quero rancar"... Aí a gente obedece o paciente, ele... Ele pede, a gente... Muitas vezes... A gente quer fazer uma coisa ele quer outra, né...

P: Com relação à cárie, ou tratamento em geral, você consegue pegar, perceber algum tipo de sofrimento dos pacientes maior com alguma coisa?

E: É, tem alguns pacientes quee... Acho que por histórico deles mesmo e ter medo, de tratamento né, o que eu vejo às vezes é isso, restaurações simples eles tem pavor, alguns pedem anestesia pra qualquer tipo de intervenção... Outros tem maior tolerância... Padrão normal que ocorre em todo lugar, né...

P: E com relação às gerações mais novas, os pacientes mais novos, comparando com o pessoal adulto, em termos de prevenção de cárie, de controle por parte deles você vê alguma diferença, como é cada um deles?

E: Eu acho que em geral tem melhorado... Mas existem algumas comunidades... Não sei se pela condição social, a portelinha aqui do lado... Porque a gente não sabe o que a pessoa paaassa, o que a família paaaassa, a dificuldade às vezes em comprar escova, a gente dá, tem escova disponível, então... Muitas vezes não é nem a dificuldade de comprar, porque é disponível, a gente sempre oferece... Às vezes tem uns que queixam que não tem dinheiro pra comprar pasta de dente ou fio dental, quando tem a gente doa também... Prefeitura sempre disponibilizou boa parte das coisas. Nesse ponto não há queixa em relação ao serviço público. Mas aí você, o que você faz com a escova, com a pasta e o fio dental também é importante, né... Às vezes a técnica correta, se escova ou não, né... Então os casos mais complexos até hoje veio dalí e daqui do bairro também... Que às vezes a criança, o pai fala que a criança não gosta, ela chooora, e por isso não escova... Às vezes tem que mandra pro ceozinho, e de lá mandar pra (HIDDEN)... E não tem muito o que fazer às vezes, porque... Às vezes você amarrar uma criança, conter ela, muitas vezes o pai não gosta, a mãe não gosta, mesmo que seja pro bem dela, às vezes você... A maneira como o pai e a mãe vê isso, talvez não seja uma coisa... É melhor, eu acho melhor um especialista tomar frente disso...

P: Nesses casos de não resolução do problema da cárie, você conseguiu identificar alguma causa maior com relação à isso?

E: É difícil porque eu não converso- eu converso o essencial com o paciente, sabe? Não fico puxando muito assunto, então... Difícil saber, às vezes ficar pes... Perguntando coisas sobre a vida dele, ele não quer falar... Então é melhor... Se ele pedir ajuda você esclarece, mas... Se ele não quiser falar, deixa ele na paz...

P: No dia a dia você costuma paciente assim que é difícil de você conversar com ele, o cara quer ficar na dele lá...

E: É, acho que a maioria gosta mais de... Ficar tranquilo, né... A gente respeita, qualquer tipo de pessoa, qualquer tipo de comportamento, a gente tem que respeitar.

P: Pergunto sobre mais casos que marcaram

E: Ah às vezes dificuldades comuns que a profissão tem né, como todo mundo tem, a gente tem também mas assim...

P: Pergunto sobre algo a acrescentar ou que queira falar, mas encerra.
