# Supplementary material for: How Brazilian dentists work within a new community care context? A qualitative study
Source: PLoS One. 2019 May 8;14(5):e0216640. doi: 10.1371/journal.pone.0216640 (PMC6505932; doi:10.1371/journal.pone.0216640)
Supplement: S3 File — Script used to conduct the interviews. (DOCX) [file pone.0216640.s003.docx]

**INTERVIEW GUIDE**

Interview number:__________.

Address: ___________________________________________________________.

Date _____ / _____ / __________.

Start: ______:______. End: ______:______. Duration: ___________ min.

**A) Interviewee Personal Identification Data:**

1. Name: ____________________________________________________.

2. Contact Address: ______________________________________________.

3. Sex: ___________________.

4. Date of birth: ______ / ______ / ________.

5. Schooling: ______________________________________________________.

6. Specialist? Which area? _________________________________.

7. University and year of graduation: _______________________________.

**B) Trigger question**

1- How do you feel about caries and what is your experience regarding its treatment?

**C) Further questions**

2- Have you come across patients that always have new carious lesions at follow-up appointments? How do you feel about those?

3- When applying preventative measures, we use strategies such as oral hygiene or diet, what do you think about this? Tell us your experience on that here.

4- How do patients with caries deal with their diagnosis?

5- What are your experiences on caries counseling and patient counseling?

6- In our day-to-day clinical lives, we often go through experiences that mark us. Please tell us something that has affected you in caries patients.

7- Would you like to comment on something that you have not been asked or is there anything that you would like to add?

**C) Observation and self-observation data of the interviewer:**

1. Personal presentation of the subject, his / her overall behavior, body expressions, hand gestures, facial mime, looks, style and speech alterations (silences, impaired speech, language lapses and other acts, inhibited and uninhibited settings, changes in vocal tone and volume), laughter, smiles, cries and similar manifestations.

__________________________________________________________________

__________________________________________________________________

__________________________________________________________________

2. Counter reactions / manifestations by the interviewer.

___________________________________________________________________

___________________________________________________________________

___________________________________________________________________
